# Extensive variation in sperm morphology in a frog with no sperm competition

**DOI:** 10.1186/s12862-016-0601-8

**Published:** 2016-02-01

**Authors:** Kathryn A. Stewart, Rachel Wang, Robert Montgomerie

**Affiliations:** Department of Biology, Queen’s University, Kingston, ON K7L 3N6 Canada; College of Environmental Science and Engineering, Tongji University, Shanghai, 1239 Siping Rd, P R China

**Keywords:** Gamete evolution, Sperm morphology, Sperm abnormalities, Sperm competition, Genetic drift, Frogs

## Abstract

**Background:**

Recent comparative studies of several taxa have found that within-species variation in sperm size decreases with increasing levels of sperm competition, suggesting that male-male gamete competition selects for an optimal sperm phenotype. Previous studies of intraspecific sperm length variation have all involved internal fertilizers where some other factors—e.g., sperm storage and sperm movement along the walls of the female’s reproductive tract—probably also influence and reduce sperm size variation. Thus external fertilizers, where those factors are absent, might be expected to exhibit even more variation when there is little or no sperm competition. To test that idea, we studied the sperm morphology of a North American chorus frog, the spring peeper (*Pseudacris crucifer*), a species in which males encounter little or no sperm competition.

**Results:**

As expected, sperm size was highly variable in the spring peeper, largely due to variation in flagellum length within and among individual males, among populations and between mitochondrial lineages in southwestern Ontario. In addition, a large proportion of spermatozoa in all males was abnormal in such a way that the ability of abnormal spermatozoa to fertilize was probably compromised. There were no differences in the frequencies of abnormalities among populations or mitochondrial lineages.

**Conclusions:**

In the absence of sperm competition, we suggest that genetic drift has probably played a role in the generation of diversity in sperm morphology in this species, potentially resulting in the observed differences among populations. Such interpopulation difference in sperm morphology might be expected to increase the degree of reproductive isolation between populations even before other isolating mechanisms evolve.

**Electronic supplementary material:**

The online version of this article (doi:10.1186/s12862-016-0601-8) contains supplementary material, which is available to authorized users.

## Background

Recent studies of birds [1–5], mammals [6–8], and invertebrates [9] have found that variation in sperm morphology within species is influenced by sperm competition. Thus species that experience more intense sperm competition show less variation in sperm length, presumably due to stabilizing selection for an optimal sperm phenotype. Across species of passerine birds, for example, a four-fold difference in the coefficient of variation (CV) in total sperm length has been described [1, 2], and about 75 % of the variation among species can be explained by indices of the intensity of sperm competition (e.g., combined testes mass controlling for body mass or rates of extrapair paternity). The same pattern has also been found within one of those species (barn swallow, *Hirundo rustica*) where the CV of sperm length declines as the intensity of sperm competition increases across six populations in Europe, the Middle East and North America [4]. Within-male variation in sperm length showed the same pattern across passerine birds [2], with the CV of sperm length within males declining as the intensity of sperm competition increases.

In the present study, we quantified the variation in sperm morphology (including morphological abnormalities) within and among males, as well as among populations and mitochondrial lineages, of the spring peeper (*Pseudacris crucifer*), a small, semi-terrestrial chorus frog whose geographical range spans most of eastern North America south of the boreal forest [10, 11]. Our field observations indicated that there was little or no sperm competition in this species, giving rise to our prediction that there would be extensive variation in sperm morphology based on those previous studies of birds. This is the first study of sperm length variation in an external fertilizer, explicitly testing the prediction that variation will be high when the intensity of sperm competition is low. While studies of sperm length variation in internal fertilizers have been consistent and informative, it is likely that sperm size is constrained in those taxa by factors that influence sperm storage and movement along the walls of the female’s reproductive tract. External fertilizers have no such constraints and might be expected to exhibit even more variation when sperm competition is relaxed.

**Fig. 1:**
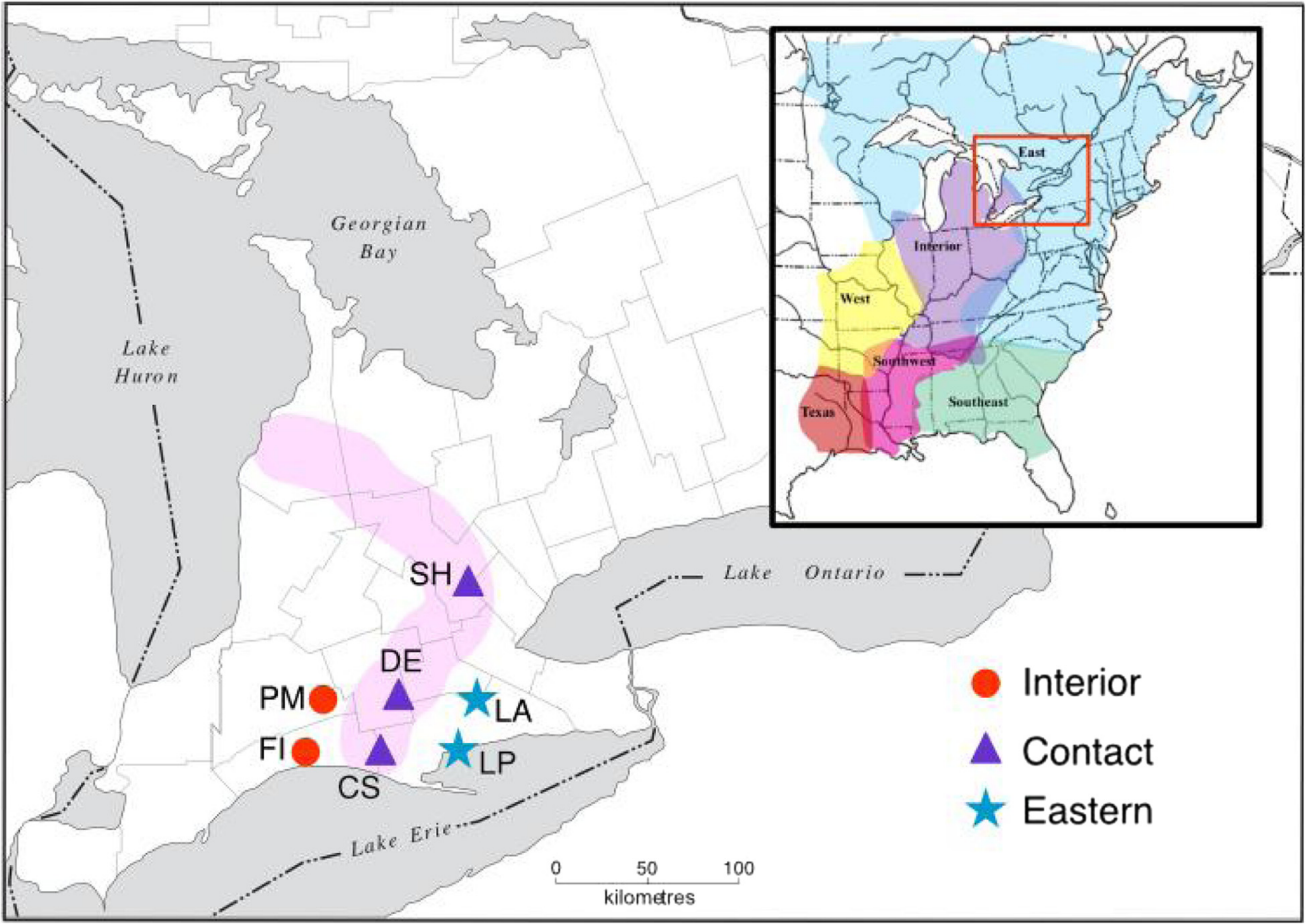
Map showing sampling localities for reproductively active male spring peepers (*P. crucifer*) in southwestern Ontario, Canada. The area of secondary contact between the Eastern and Interior mitochondrial DNA lineages is shaded in pink, following [11, 14]. Sampled populations are Pond Mills (PM), Fingal (FI), Starkey Hill (SH), Dereham (DE), Calton Swamp (CS), Lafortune (LA), and Long Point (LP) (see [14] for details). The red rectangle on the inset map shows the area of the main map, as well as the ranges of the six mitochondrial lineages of spring peepers in eastern North America [11]. Map modified with permission from: Southern Ontario-Regional Municipality Boundaries [pdf file]: Brock University Map, Data & GIS Library. Available: Brock University Map, Data & GIS Library at http://www.brocku.ca/maplibrary/maps/outline/Ontario/Sontbase.pdf (Accessed August 15, 2015)

To test that prediction, we studied the spermatozoa of several populations in southwestern Ontario (Fig. 1), where spring peepers begin breeding in early spring (late March and early April), often in large choruses of up to thousands of males in a single pond. Although adult males usually outnumber females by 9:1 or more in breeding aggregations [12], each male occupies a small territory (0.5 - 6 m^2^ depending on population density [13]) that he defends by calling, thereby minimizing the incidence of physical encounters with other males. Within these choruses, female spring peepers are attracted to individual males based on the attributes of their loud and repeated calls [14]. Once in the water, a female typically swims toward her chosen mate, touches him, and allows him to copulate [12]. With the male clasped to her armpits (axillary amplexus), the female swims while laying a clutch of 800–1000 eggs (each approx. 1-mm diameter; [15]) as the male ejaculates and fertilizes the clutch.

When choruses are most active (on warm, wet, moonless nights), there is sometimes a smaller, silent, satellite male near (within 20 cm) the territorial male who will occasionally intercept females approaching the calling male [16]. Despite this obvious male-male competition for access to females, males do not attempt to amplex females simultaneously. Importantly, females typically mate only once each year, and deposit eggs singly or in very small clusters (a unique mode of oviposition compared to congeners [17]) at the bottom of the pond among grasses or other aquatic plants [15], effectively eliminating clutch piracy or cryptic female choice (but see [18]). Indeed, during hundreds of hours of field collections and behavioural observations during 14 breeding seasons from 2000–2013, we have never observed more than one male at a time attempting to amplex a female. We conclude from this that each clutch of eggs is fertilized only by a single male. Spring peepers also have small testes (2.4 mg) relative to their snout-vent length (26.1 mm), which is approximately half the testes mass predicted from a comparison of 10 species (see Additional file 1) in the same family (Hylidae) where females are apparently known to mate multiply [19]. This analysis further suggests that there is little or no polyandry or sperm competition in this species.

## Materials

### Ethics and legal statement

All research completed during this study complied with Canadian laws and regulations of the Canadian Council on Animal Care and did not involve the study of endangered or protected species. Queen’s University’s Animal Care Committee approved the protocol (Lougheed-2008-059-Or) used to sample the animals. Collections were obtained under a Wildlife Scientific Collector’s Permit from the Ontario Ministry of Natural Resources (1044736) to conduct research on either private land with the permission of land-owners, on publicly-owned property, or on rights-of-way beside roads.

### Study populations and field methods

For this study we sampled male spring peepers from 11 April to 17 May 2011 during their breeding season in southwestern Ontario. We hand-captured 89 reproductively active territorial males that were vigorously calling in breeding assemblages situated in three contiguous geographic regions that had been previously defined by analysis of their mitochondrial-DNA (mtDNA) [11, 14]: 21 frogs from 2 populations of the Eastern mtDNA lineage, 22 from 2 populations of the Interior mtDNA lineage, and 46 from 3 populations in the Contact zone between those two lineages (Fig. 1).

To control for any variation in sperm morphology due to variation in the size and condition of the frogs, we measured the following traits on each male: snout-vent length (SVL), head width, radioulnar length, femur length, tibiotarsus length, and foot length using a digital caliper (±0.2 mm); and mass (±0.02 g) using a Pesola™ 10-g scale.

Males were sacrificed (by double-pithing) on the day of capture and their testes immediately removed. In many individuals, the right testis was much smaller than the left, a common pattern in frogs [20, 21], and 3 males had no right testis (2 from the Contact zone and 1 from the Interior lineage). To avoid possible biases due to the right testis not functioning properly [22], we studied spermatozoa only from the left testis, which we macerated, using a blunt probe, in a glass Petri dish with 5 mL of de-chlorinated water. A droplet of the sperm suspension was then placed on a microscope slide, allowed to air dry, and fixed in a 5 % formalin solution [23]. After formalin fixation, microscope slides were stained [24] with a 4:1 mixture of 0.25 g/L basic fuchsin and 0.5 g/L methylene blue to improve contrast when viewed under a Carl Zeiss Axio Observer microscope.

### Sperm abnormalities

Under 630X magnification, we inspected 10 haphazardly-chosen fields of view on each slide of spermatozoa (1 slide per male), where the observer was blind to the identity of the male. In each field of view, we counted the number of spermatozoa (approximately 100 for each male) and the number of spermatozoa that had any abnormal morphologies (two heads, two tails, no head, no tail, cytoplasmic droplets; [25]). Although spermatozoa with no tail and no head can occasionally result from normal sperm being damaged during slide preparation, we have no reason to expect that any such damage would be biased to any particular male or population.

### Sperm morphometry

For all but one male (*n* = 85), we also measured up to 10 morphologically normal sperm (ie., sperm without any abnormalities and not obviously damaged), one spermatozoon for each of the 10 fields of view examined (see also [2, 4] for justification of this sample size per male). We photographed each field of view digitally and used ImageJ (version 1.45; [26]) to measure the following morphological traits to the nearest 0.1 μm on each spermatozoon: head length, head perimeter, and flagellum length. We calculated total sperm length as the sum of head and flagellum length [27]. We could not distinguish midpiece from tail so ‘flagellum’ length is a composite of those two traits. For each individual male we calculated the mean of each trait for further analysis.

### Data analysis

We first checked the distribution of each morphological variable (both sperm and body) among frogs to ensure that it was unimodal and Gaussian, and then assessed collinearity between the variables. Two male spring peepers from the Eastern lineage were sampled on 11 April (17 days earlier in the breeding season than any other male that we studied) yielded low sperm numbers and we were able to measure only a small sample (*n* = 2 and 5) of normal sperm for each male. One of these individuals was also a consistent outlier on almost every metric of sperm morphology. A third male from the same lineage, but a different population, also yielded only 2 normal sperm that we could measure and was an outlier with respect to sperm head length, so we removed it from the dataset as well, leaving 86 males for which we had sperm and body size measurements, accompanied by an assessment for sperm abnormalities for all but one of those males. We thus removed all three of these males from further analysis as they may not have been reproductively mature.

To obtain a simple measure of overall body size, we performed a Principal Component Analysis (PCA; varimax rotation, 2 axes) on a correlation matrix of the seven body measurements for all individuals. As an estimate of ‘body condition’, we used the scaled mass index (SMI, where b_SMA_ = 1.539; [28]).

To evaluate the factors influencing each sperm trait, we selected the best-fitting model as the one with the lowest corrected Akaike Information Criterion (AICc), but we report all of the top models (AICc < 2) and their statistics in Additional file 1. We considered all top models (ΔAICc <2.0) to be statistically indistinguishable given the data [29], but our conclusions are not affected by choosing any of these top models.

To determine how much of the variation in sperm morphology was due to differences among populations, we compared both populations and mitochondrial lineages (and their contact zone) using a hierarchical approach. To compare populations, we fit generalized linear models (GLM) with population as a fixed effect. To compare lineages, we fit generalized linear mixed models (GLMM) with population as a random effect. When the effect of population was significant, we used Tukey HSD *post hoc* analyses (at alpha = 0.05) to identify which populations were significantly different. We used F and log-likelihood ratio chi-square (LR *χ*^2^) tests to compare models with and without predictors of interest, as appropriate to the model.

We calculated coefficients of variation either from raw data (CV = SD/mean x 100 %) or from the root mean squared error (RMSE) of linear models (CV = model RMSE/mean of the response variable x 100 %), using a simple function (see Additional file 1). When sample sizes were <25, we calculated CV_adj_ (CV × 1 + 1/n) to correct for the underestimate due to small sample sizes [30].

We performed these analyses in R (v 3.2.2; [31]) and provide the raw data (mean values per male) as well as the R code used for analysis in DRYAD (doi:10.5061/dryad.ss8t1). Both R code and the results of all analyses are provided in Additional file 1. Descriptive statistics are presented as means [95 % CL].

## Results

### Frog body size and condition

In the Principal Components Analysis of 6 body morphometric variables (see Additional file 1), PC1 accounted for 49 % of the total variation, largely capturing variation in overall body size (loadings on PC1 all >0.75 for SVL, head width, radioulnar length, and foot length). PC2 captured 26 % of the variation, with hindlimb morphology (femur length, tibiofibula length) having the highest loadings on that axis. Thus we used PC1 scores in all analyses as an overall measure of body size. Body condition (SMI) was not significantly correlated with body size PC1 (*r* = −0.15, *P* = 0.16, *n* = 86). In subsequent models, both body size (PC1) and condition (SMI) were tested to control for their potential influence on sperm morphologies.

### Sperm size

**Fig. 2:**
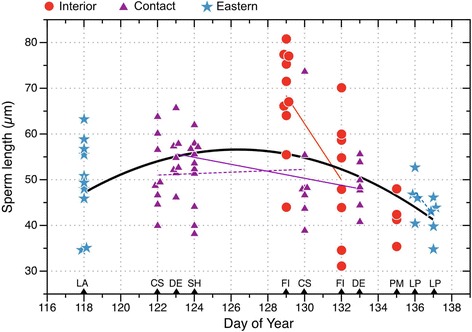
Seasonal variation in sperm length in spring peepers from 7 populations in 2 mitochondrial lineages (Interior, Eastern) and their Contact zone in southwestern Ontario (*n* = 86 males). Linear regression lines are shown for populations sampled on two different days (solid = significant (*P* ≤ 0.05), dashed = non-significant; see Additional File 1). The black curvilinear regression line is from a model (R^2^ = 0.17, F_2,83_ = 8.24, *P* = 0.0005) fitted to all of the data with date as a quadratic predictor, but not controlling for body condition. Overlapping data points are jittered horizontally for clarity. Sampling dates and localities are indicated by black triangles on the x-axis: Pond Mills (PM), Fingal (FI), Starkey Hill (SH), Dereham (DE), Calton Swamp (CS), Lafortune (LA), and Long Point (LP) (see [14] for details)

Total sperm length clearly varied with season in a nonlinear fashion (Fig. 2), so we tested date as a quadratic predictor (i.e., date + date^2^). The best-fitting model to predict sperm length (Table 1) included date and date^2^, as well as SMI, thus we tested date as a quadratic predictor in all subsequent models of sperm morphology. Since flagellum length and sperm length were highly correlated (*r* = 0.99, *P* <0.0001, *n* = 86 males), we use only sperm length in subsequent models. See Additional file 1 for analysis of the responses to variation in sperm flagellum length, showing the same patterns as for total sperm length.

**Table 1:** Best-fitting general linear models to predict sperm morphological traits. Predictors (but not the intercept) are listed for each model. Estimates with 95 % CLs that do not include zero are significant predictors (bold). See Additional file 2: Table S1 for all top models (ΔAICc < 2) predicting each response variable

Response	Predictors	Estimate [95 % CL]
total sperm length (R^2^ = 0.21)	**date** ^**2**^	−0.15 [−0.22, −0.08]
	**date**	36.7 [18.9, 54.6]
	**body condition (SMI)**	−9.74 [−19.1, −0.38]
sperm head length (R^2^ = 0.06)	**date**	−0.07 [−0.13, −0.008]
sperm head perimeter (R^2^ = 0.07)	**date** ^**2**^	−0.0005 [−0.0009, −0.0000023]
	body size (PC1)	0.51 [−0.17, 1.18]

**Fig. 3:**
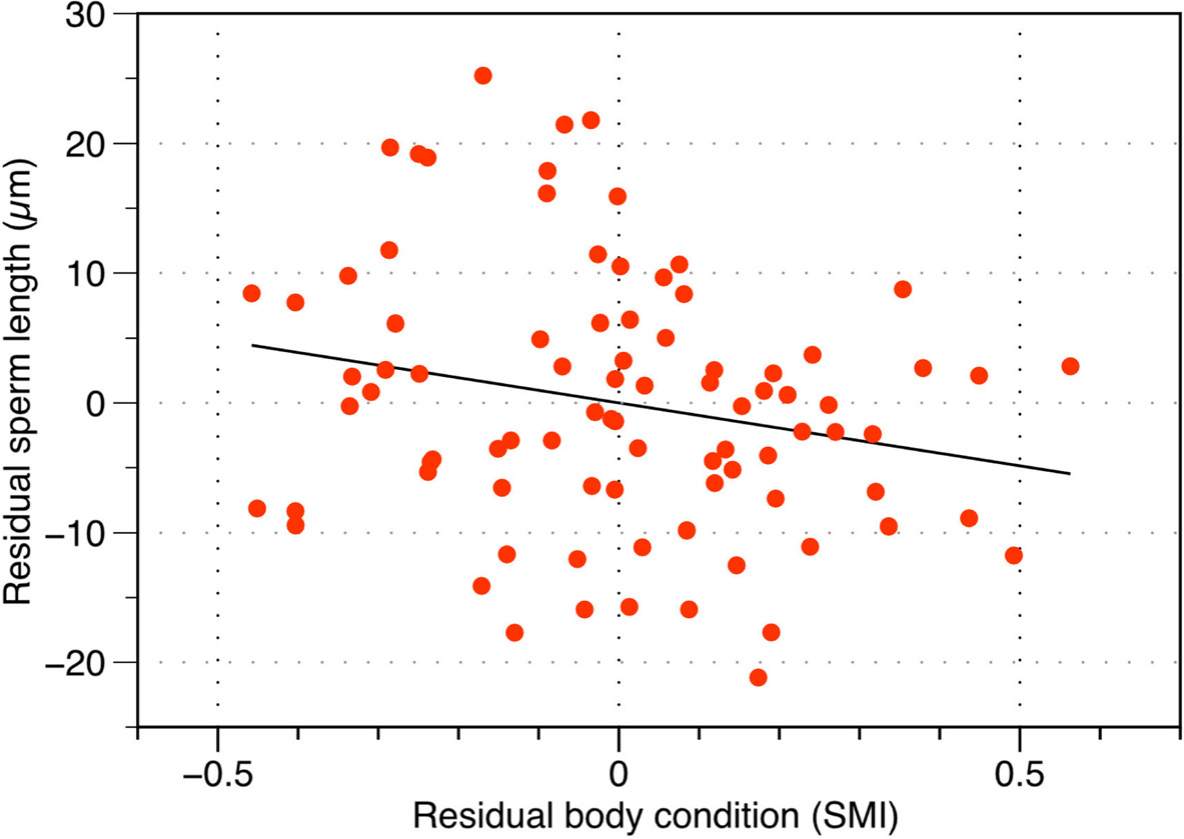
Partial regression plot showing the effect of body condition on total sperm length, controlling for date as a quadratic predictor. Each data point is the mean length of up to 10 sperm measured for each of 86 frogs

Total sperm length reached its peak in the middle of the breeding season (Fig. 2), and was significantly and negatively related to SMI, controlling for date, in the best-fitting model (Fig. 3, Table 1). Thus frogs in the best condition had sperm that averaged about 10 μm shorter than frogs in the worst condition (Fig. 3). Overall, body condition explained only 4 % of the variation in sperm length, date explained 28 % of the variation, and 68 % of the variation remained unexplained. Body size was also included as a predictor of sperm length in some top models (Additional file 2: Table S1), but was not statistically significant.

The among-male coefficient of variation (CV) for sperm length (controlling for date and date^2^) was relatively high, at 18.7 % (*n* = 86 males). The average within-male CV_adj_ for sperm length was even higher, at 36.0 %, in 52 males for which we had measured 10 spermatozoa.

Sperm head length and perimeter also changed slightly but significantly as the breeding season progressed in the best-fitting models (Table 1). Neither body size nor condition was significant in the best-fitting models, though both were represented in some of the top models (*Δ*AICc < 2; Additional file 2: Table S1). Sperm head length and perimeter were also not significantly related to flagellum length (*P* > 0.50), controlling for date, body size and body condition (Additional file 1).

The among-male coefficients of variation (*n* = 86 males) for both sperm head length (CV = 7.22 %) and sperm head perimeter (CV = 6.65 %), controlling for other predictors in the best-fitting models (Table 1), were less than half the CV for total sperm length (Additional file 1). The average within-male CVs (*n* = 10 spermatozoa from each of 52 males) for sperm head length (CV_adj_ = 11.9 %) and sperm head perimeter (CV_adj_ = 12.0 %) were also much less than that for total sperm length.

### Sperm abnormalities

A large proportion of the spermatozoa of most males was abnormal (range 27–100 % of ~100 sperm for each of 86 males; Table 2). All males had at least a few sperm with cytoplasmic drops (range 7.5–96.5 % of spermatozoa), and all but 5 males had some sperm without tails (Table 2). The remaining abnormalities were much rarer and occurred in only a small percentage of the males (Table 2).

**Table 2:** Proportion of males sampled that had at least one sperm in different categories of abnormality, and the mean proportion of sperm per male in each category. All abnormalities are included in the first category; some males had sperm with abnormalities in more than one category

Abnormality	Percent of males	Proportion of sperm per male
	(*n* = 85)	mean [95 % CL] *n* = 85 males
all abnormalities	100	0.567 [0.533, 0.601]
cytoplasmic drop	100	0.446 [0.410, 0.482]
no tail	94.1	0.113 [0.092, 0.134]
two tails	42.4	0.013 [0.007, 0.018]
no head	50.6	0.012 [0.009, 0.016]
two heads	2.4	0.0003 [−0.0001, 0.0007]
other	7.1	0.0009 [0.0002, 0.002]

The proportion of abnormal sperm in a male’s ejaculate was not related to his body condition (GLM with binomial error, corrected for over-dispersion: LR *χ*^2^ = 1.46, *P* = 0.23), nor was the proportion of sperm with any particular abnormality related to male body condition (see Additional file 1).

### Comparing populations

**Fig. 4:**
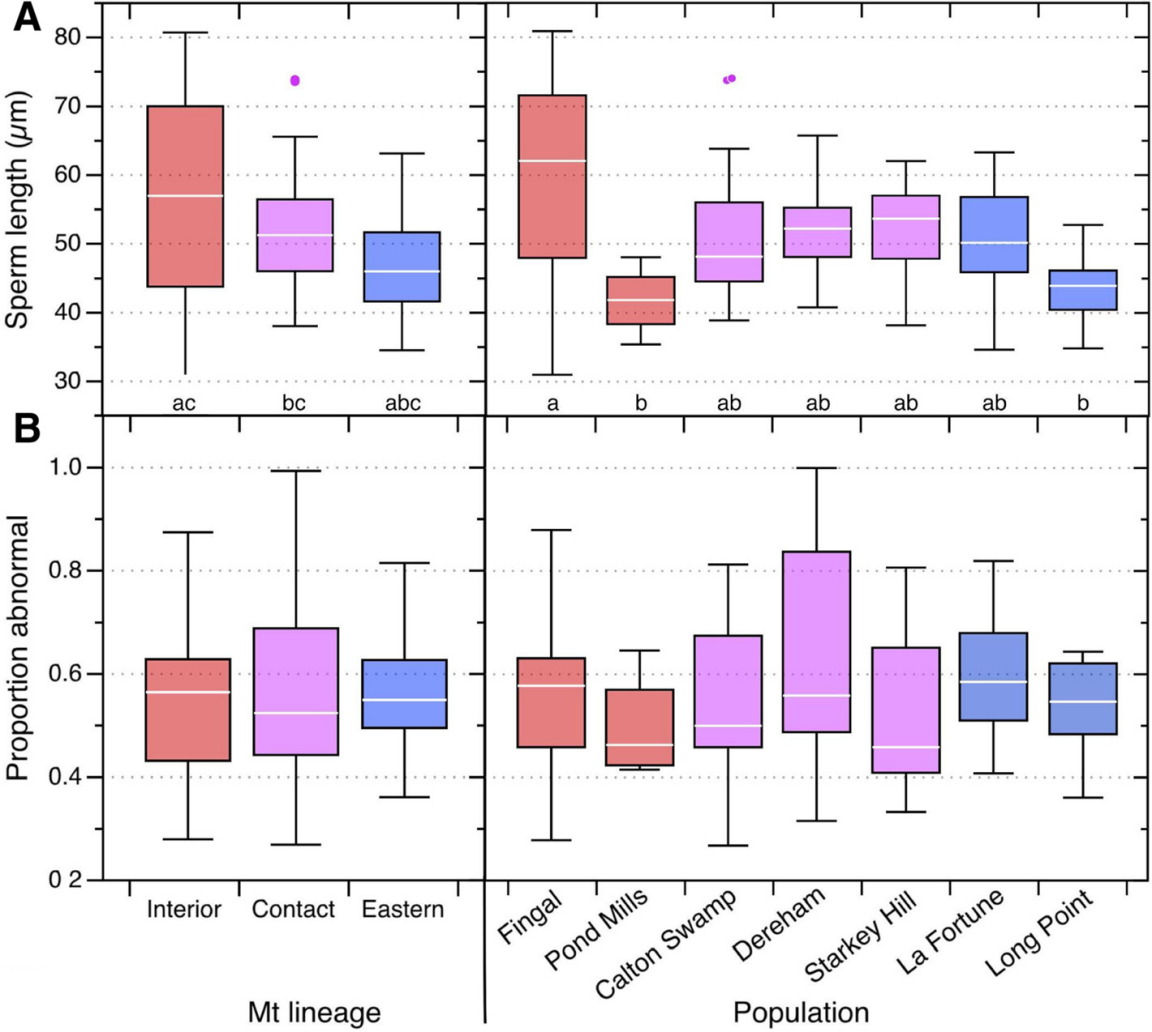
Variation in **a** sperm length (*n* = 86) and **b** the frequency of sperm with any abnormalities (*n* = 85) within and between mtDNA lineages and their contact zone (left), as well as within and among populations within those lineages (right). Lineages or populations (Tukey box plots) that do not share the same lower case letters within panels are significantly different (Tukey HSD *post hoc* tests) based on statistical models described in the text

Sperm length varied significantly among populations (F_6,76_ = 3.03, *P* = 0.01) in the best-fitting general linear model, controlling for body condition and season (Additional file 3: Table S2, Fig. 4). One population in the Interior lineage (Fingal) had the longest sperm, significantly longer than sperm from both the other population (Pond Mills) in that lineage and from the Long Point population in the Eastern lineage (Fig. 4a). No other populations differed significantly in sperm length. The Pond Mills and Long Point populations differed significantly in sperm head length in the best-fitting model (Additional file 3; Tukey HSD test, *P* < 0.05), but there were no other differences between populations in either the length or the perimeter of the sperm head (Additional file 1, Additional file 3: Table S2).

Controlling for differences among populations, the among-male coefficient of variation (CV) for sperm length (controlling for both date as a quadratic predictor and body condition) remained relatively high, at 16.8 % (*n* = 86 males).

The proportion of a male’s sperm that were abnormal (Table 2) did not differ significantly among populations (GLM with binomial error, corrected for over-dispersion: LR *χ*^2^ = 7.42, *P* = 0.28), nor did any of the different types of abnormality differ in frequency among populations (Additional file 3: Table S2). In general, the proportions of sperm that had any abnormality varied widely among males within populations, and the average levels of abnormality were very similar among populations (Fig. 4b).

### Comparing mitochondrial lineages

Sperm length was significantly different among mitochondrial lineages and their contact zone in the best-fitting model (GLM: LR χ^2^_1_ = 7.44, *P* = 0.0002), with date a significant linear predictor (Additional file 1). In that model the interaction between lineage and date was also significant (LR χ^2^_1_ = 24.02, *P* < 0.0001) making the significant main effect of lineage difficult to interpret. Tukey HSD *post hoc* contrasts showed that sperm length in the Interior lineage was significantly greater than in both the Contact zone and the Eastern lineage (*P* < 0.001), but not between the Eastern lineage and the Contact zone (*P* =0.18) in the best-fitting model, controlling for date. Sperm length was highly variable overall, with a CV of 17.0 %, controlling for date, lineage and the interaction between date and lineage.

The proportion of abnormal sperm per male did not differ between mitochondrial lineages and their contact zone, nor did the proportion of sperm that lacked tails, or had cytoplasmic drops, or any other abnormality (Additional File 1).

## Discussion

### Variation in sperm size

As predicted for species with low levels of sperm competition, the total length of sperm produced by male spring peepers is highly variable both among and within individuals. A small proportion of the sperm length variation among males is due to body condition, seasonal effects and population differences across the geographic region that we sampled, but most of that variability (>60 %) is due to unexplained variation in sperm flagellum length. We expect that this unexplained variation is the result of relaxed selection for an optimal sperm phenotype, a consequence of the absence of sexual selection in the form of sperm competition (see also [32, 33]).

Without selection from sperm competition, flagellum length is probably most susceptible to change among individuals because flagella are relatively simple structures designed to propel sperm, with flagellum size usually an important predictor of fertilization success when sperm compete [34] (but see [35]). In contrast, sperm head morphology may be constrained as the head packages the highly condensed DNA and is unlikely to vary without a high fitness cost. In spring peepers, sperm head length is much less variable than flagellum length (CV for sperm head length was less than half that of flagellum) as also seen in passerine birds [5].

Among-male sperm length variation in the spring peepers that we studied was almost ten times that in the quacking frog (*Crinia georgiana*; CV = 1.9 % calculated from data extracted from Fig. 2a in [36]), a species in which 50 % of matings are polyandrous. These two species, therefore, show the same pattern described for birds [1, 2] and insects [9], where sperm size variation declines with the intensity of sperm competition. We know of no other estimates of sperm length variability in frogs, so we cannot yet conclude that this is a general pattern in this taxon without further investigation.

The among-male CV in sperm length of spring peepers is more than double that in birds known to have low levels of sperm competition (8.0 % calculated from data in Fig. 1a in [1, 2]). The average within-male variation in the CV of sperm length was even higher (27.7 %), almost three times that reported for birds with low sperm competition risk (e.g. 11.2 % for the bullfinch, *Phyrrula phyrrula,* in [3]). We know of only one other study that has looked at sperm length variation in a species—the ant *Trachymyrmex* sp. 3—reported to have low levels of sperm competition [9], where the among-male CV for sperm length (6.57) was less than half that in spring peepers. This raises an intriguing quandary: why is the variability in sperm length so high in the spring peeper?

One possibility is that external fertilization imposes fewer constraints on changes in sperm morphology than does internal fertilization when selection due to sperm competition is relaxed. Evidence from an externally fertilizing fish (CV = 6.7 % in 120 parental male bluegill *Lepomis macrochirus*; R. Montgomerie unpublished data) and the quacking frog (CV = 1.9 % [36]), both of which experience high levels of sperm competition, suggests that external fertilizers might generally have high levels of variability in sperm length. In contrast, the CV of sperm length in birds experiencing the highest levels of sperm competition is much lower (e.g., 0.5 % for the common reed bunting, *Emberiza schoeniclus*; [2])

When fertilization is internal, sperm storage by the female appears to influence sperm morphology in ways that would not occur in external fertilizers [37, 38] and may further constrain variability when selection is relaxed in the absence of sperm competition. Thus in internal fertilizers, variation in sperm length away from some narrow optimum for female storage may well result in reduced fitness by preventing fertilization even when a female mates with only one male. Certainly a comparison of sperm size variation in external and internal fertilizers across species with a range of sperm competition levels would be informative in this regard. We would predict relatively high sperm length variation in external fertilizers across all levels of sperm competition.

### Sperm abnormalities

We also found an unexpectedly high incidence of sperm abnormalities in each male spring peeper’s semen, with no differences among populations or mitochondrial lineages (or their contact zone) in the frequencies of those abnormalities. The high proportions of sperm abnormalities further suggest that the selection of sperm quality during spermatogenesis production has been weak. Our findings thus raise the interesting possibility that it may be more efficient for males to produce large numbers of sperm with a high proportion of debilitating morphological abnormalities than it is to produce relatively few high-quality sperm (see also [33]).

Sperm abnormalities are relatively uncommon in most species, and selection might be expected to favour the production of normal spermatozoa because aberrant sperm are unlikely to be useful for fertilization. In the absence of selection for high numbers of sperm in an ejaculate (as is the case when there is no sperm competition), sperm abnormalities might accumulate as long as the reduction in the proportion of sperm that are viable does not hinder male fertilization success. Alternatively, Parker and Begon [32] have argued that an increase in variation might be due to relaxation of the conflict between haploid and diploid control of sperm production when the intensity of sperm competition is also low. As they point out, however, their models predict an increase in variation in sperm size or number when sperm competition is relaxed, but the relative influence of haploid and diploid gene expression on sperm morphology is as yet unknown.

Inbreeding may also result in the accumulation of sperm abnormalities as shown in some endangered mammal species [39], but our spring peeper population sizes were on the order of 10,000 frogs where the effects of inbreeding seem unlikely. In addition, both genetic erosion through sequential founder events during range expansion from glacial refugia and contemporary range fragmentation may reduce population genetic diversity, but previous work on the spring peeper did not reveal any evidence of inbreeding depression [14].

### Differences between populations

The variation in mean sperm size among populations and mitochondrial lineages is also intriguing and unexpected. Although only a few of those differences were statistically significant (Fig. 4a), our sample sizes were small enough that statistical power was low, and ultimately the magnitude of those differences can only be assessed with larger samples.

Sperm morphology was once argued to be relatively unaffected by natural selection or genetic drift, with interspecific divergence due to evolutionary forces (e.g., environmental factors or body condition [40]) predicted to arise slowly compared to differentiation due to sexual selection. The proximity of our spring peeper populations to one another—less than 75 km apart (Fig. 1), with no obvious differences in climatic or aquatic environments—supports our contention that natural selection is unlikely to have been the primary driver of gamete differentiation. Interestingly, intraspecific differences in sperm size between populations of a single species have rarely been documented, and where such variation occurs the differences are difficult to explain. For example, subspecific differences in sperm midpiece size has been observed in the bluethroat (*Luscinia svecica*) [41], and the authors likewise argue that these differences may be due to genetic drift but there is also evidence that the intensity of sperm competition may vary among those subspecies. In the quacking frog, there are significant differences in sperm length among populations <250 km apart in Western Australia [36], with similar assertions—but no supporting data—that sperm competition (or possibly fertilization environment) may differ enough between populations to exert some selective force.

In the spring peeper, it is possible that any between-population divergence in sperm size could be attributable to other mechanisms such as indirect natural selection via genetic hitchhiking, or as a pleiotropic by-product of selection on other traits [42]. For example, some studies have found that sperm length varies with body size among species (allometry; [43, 44]), while others have failed to find such a relationship [45, 46], including in frogs [47]. However, sperm size in spring peepers was not related to differences in body size between either mtDNA lineages or populations. Similarly, divergence in egg size may also cause sperm trait differentiation, although the within-clutch CV in spring peeper egg diameter averaged only 3.3 % in Florida [48], suggesting that this relatively invariable trait is unlikely to exert strong directional selection on sperm traits (although further research would indeed be required to completely discount this relationship).

In the absence of direct evidence for sexual or natural selection, genetic drift seems the most likely explanation for intraspecific variation in sperm size among our spring peeper populations. Drift might not normally be expected to influence sperm traits, primarily because such drift should be opposed by both natural and sexual selection, especially in large populations; thus some have argued that drift is probably an uncommon mechanism during speciation in general [42]. Still, evolutionary forces such as drift and selection often work in concert to produce divergent adaptive maxima [49]. Although evidence in nature is limited, genetic drift and founder effects should be common in populations that have undergone geographical isolation in, and subsequent expansion from, multiple glacial refugia and thus should facilitate rapid speciation [43, 50]. Indeed, Lüpold and colleagues [23] proposed that historical processes associated with postglacial population expansion and sequential founder events may underlie some of the geographical patterns evident in the sperm morphology of red-winged blackbirds (*Agelaius phoeniceus*). However, strong evidence for sperm competition, the lack of high-resolution molecular markers, and a tight association between sperm length and body size complicate assertions that genetic drift was the primary driver of sperm divergence in that species.

Differentiation in sperm morphology between populations of spring peepers therefore suggests that sperm traits may sometimes evolve even without sperm competition and potentially before other isolation mechanisms. Barriers to fertilization presumably arise late in the speciation process, with some authors positing that selection against gamete wastage should favour the evolution of earlier-acting reproductive barriers [51]. Premating isolation through both acoustic differences and population-specific female preference have indeed been shown in spring peepers [14], while postzygotic reproductive barriers, as demonstrated through experimental evidence on spring peeper tadpoles, remains incomplete [52]. Divergence in spring peeper external morphology is relatively cryptic and no one has yet suggested that these lineages be recognized as incipient species. However, we now have evidence that there has been some sperm differentiation between lineages that could further act as postcopulatory, prezygotic barriers to gene flow.

Sperm cells are exceptionally diverse among even closely-related species, and are subject to a host of evolutionary forces. While research into gamete diversification provides unique insights into the evolution of species, there remain gaps in our understanding, including information about which evolutionary pressures are most important in shaping sperm morphological diversity and their role in the evolution of reproductive isolation. Certainly, studies of sperm morphological diversity have been mainly descriptive, and understanding the (ultimate) significance of this variation remains a challenge. Given the detailed intraspecific phylogeny of the spring peeper in particular [14], and North American chorus frogs in general [53], there is a unique opportunity for future studies to compare variance in sperm divergence among populations, directly disentangling whether such divergence is shaped primarily via genetic drift or natural selection (see [54, 55]).

## Conclusions

The spermatozoa of the spring peeper are uncommonly variable in size, with coefficients of variation in sperm length the highest yet reported in any animal, largely due to within- and among-male variation in flagellum length. Similarly, a high proportion of spermatozoa in this species have abnormalities that would prevent or hinder fertilization. Since there is little or no sperm competition in this species, we conclude that this variation is the result of relaxed sexual selection for an optimal sperm phenotype. As a further consequence of that relaxed selection, some populations in southwestern Ontario have diverged in mean sperm size, likely due to genetic drift.

### Data availability

The data analyzed in this paper have been deposited at DRYAD (doi:10.5061/dryad.ss8t1).

## Additional Files

Additional file 1:
**Statistical details, R code and output for all of the analyses reported in this paper.** (PDF 790 kb) Additional file 2: Table S1. Top models (ΔAICc <2.0) for each of the analyses summarized in Table 1. (DOCX 13 kb) Additional file 3: Table S2. Top models (AICc ≤2) testing for differences among populations in sperm size traits. (DOCX 14 kb)
