# Supplementary material for: Extensive variation in sperm morphology in a frog with no sperm competition
Source: BMC Evol Biol. 2016 Feb 1;16:29. doi: 10.1186/s12862-016-0601-8 (PMC4735968; doi:10.1186/s12862-016-0601-8)
Supplement: Additional file 1: — Statistical details, R code and output for all of the analyses reported in this paper. (PDF 790 kb) [file 12862_2016_601_MOESM1_ESM.pdf]

Additional File 1

Table of contents

1. Details of datasets and R packages used 2
1.1 Datasets 2
1.2 R details 4
1.2.1 R and package versions used 4
1.2.2 Statistical options and functions 4
2. Background 5
2.0.1 Relative testes size analysis 5
3. Materials 5
3.1 Data analysis 5
3.1.1 Examining distributions of morphological variables. 5
4. Results 13
4.1 Frog body size and condition 13
4.2 Sperm size 16
4.2.1 Analyses of the seasonal variation in sperm size traits 16
4.3 Sperm abnormalities 29
4.4 Comparing populations 50
4.4.1 Sperm size compared across populations 50
4.4.2 Sperm abnormalities compared across populations 66
4.5 Comparing mitochondrial lineages 94
4.5.1 Compare lineages with respect to sperm abnormalities 103

R code and analyses to accompany: *Stewart, Wang, and Montgomerie. 2016. Extensive variation in sperm morphology in a frog with no sperm competition. BMC Evolutionary Biology*

This file contains all of the R code and output for statistics included in and referred to in this paper. The analyses are divided into numbered sections with the titles of sections 2-4 and their next level subsections paralleling the same sections in the paper. R code is annotated to provide additional information. There is a lot of material here that is not reported in the paper, including plots to assess whether model assumptions have been met, and analyses to test for interactions etc.

1. Details of datasets and R packages used

1.1 Datasets

PEEPERS.csv has been uploaded to DRYAD with a README file that includes details on the variables (columns), as follows:

- Individual = each individual male is uniquely numbered
- Population = pond from which male was captured (all in southern Ontario, Canada)
- Lineage = based on genetic analyses, either Interior, Eastern or Contact zone where those two meet in southwestern Ontario
- Nsperm = number of spermatozoa measured
- SpHeadLen = length of sperm head (microns, μm)
- SpTailLen = length of sperm flagellum (microns, μm)
- SpHeadPerim = length of sperm head perimeter (microns, μm)
- TOTspermlen = total sperm length (SpHeadLen + SpTailLen)
- BodyMass = frog body body bass (g)
- SVL = frog snout-vent length (mm)
- HeadWidth = frog head width (mm)
- radioulna = length of radioulna (mm)
- femur = length of femur (mm)
- tibia = length of tibia (mm)
- foot = length of foot (mm)
- SMI = Scaled Mass Index (body condition)
- PC1 = Principal Component 1 (body size from PCA)
- DOY = day of the year
- DOY2 = DOY^2
- latitude = latitude (°N)
- TotalSperm = total number of sperm observed to look for abnormalities
- Abnormal = number of sperm with abnormalities of any type
- noAb = no. of sperm with no abnormalities (TotalSperm-Abnormal)
- CytoDrop = no. of sperm with ≥ cytoplasmic drops
- noCD = no. of sperm with no cytoplasmic drop (TotalSperm-CytoDrop)

- NoTail = no. of sperm with no flagellum
- no0t = no. of sperm with a flagellum (TotalSperm-NoTail)
- NoHead = no. of sperm with no head
- no0H = no. of sperm with ≥1 head (TotalSperm-NoHead)
- TwoTails = no. of sperm with 2 flagella
- no2t = no. of sperm with one flagellum (TotalSperm-TwoTails)
- TwoHeads = TwoHeads = no. of sperm with 2 heads
- no2H = no. of sperm with ≤1 head (TotalSperm-TwoHeads)
- Other = no. of sperm with some other abnormality
- noOTH = no. of sperm with no abnormalities nor any one of the other abnormalities listed above (TotalSperm-Other)
- Pabnormal = proportion of sperm that had some abnormality (Abnormal/TotalSperm)
- Pcd = proportion of sperm that had a cytoplasmic drop (CytDrop/TotalSperm)
- P0tails = proportion of sperm that had no flagellum (NoTail/TotalSperm)
- P0heads = proportion of sperm that had no head (NoHead/TotalSperm)
- P2tails = proportion of sperm that had 2tails (TwoTails/TotalSperm)
- P2heads = proportion of sperm that had 2 heads (TwoHeads/TotalSperm)
- Pother = proportion of sperm that had some other abnormality (Other/TotalSperm)

dat2: This dataset (see below) was extracted from Appendix 1 in Emerson, S.B. 1997. Testis size variation in frogs: Testing the alternatives. *Behavioral Ecology and Sociobiology* 41:227-235.

- Species = scientific name
- Testes = combined testes mass (g)
- SVL = snout-vent length (mm)

Either store these datasets in your working directory or use the following code example:
dat1 <- read.csv(file.choose())

```
dat1 <- read.csv("PEEPERS.csv", header=TRUE) #note that the 3 outliers are not in this dataset
#create data frame of Hylidae data from Emerson (1997)
Species  <- c(‘Pseudacris_cucifer','Agalychnis_callidryas','A_saltator','A_moreletii', 'Pachymedusa_dacnicolor','Phyllomedusa_savaugii','Anotheca_spinosa','Hyla_gratiosa','Osteopilus_septentrionalis','Litoria_infrafrenata','L_alboguttata')
Testes  <- c(0.0024,0.0110,0.0110,0.0450,0.1012,0.0425,0.0264,0.0216,0.0194,0.0345,0.0148)
SVL <- c(25.2,53.0,39.3,62.0,76.0,57.4,56.2,56.0,46.5,87.7,54.2)
dat2  <- data.frame(Species,Testes,SVL)
```

1.2 R details

1.2.1 R and package versions used

- File creation date: 2015-11-08
- R version 3.2.2 (2015-08-14)
- `agricolae` package version: 1.2.3
- `MuMIn` package version: 1.15.1
- `psych` package version: 1.5.8
- `car` package version: 2.1.0
- `pastecs` package version: 1.3.18
- `lattice` package version: 0.20.33
- `lmodel2` package version: 1.7.2
- `lsmeans` package version: 2.20.23
- `lme4` package version: 1.1.9
- `dispmod` package version: 1.1
- `RVAideMemoire` package version: 0.9.51
- `multcomp` package version: 1.4.1
- `dispmod` package version: 1.1

```
library(psych)
library(agricolae)
library(car)
library(pastecs)
library(lattice)
library(lmodel2)
library(lsmeans)
library(lme4)
library(dispmod)
library(RVAideMemoire)
library(multcomp)
library(MuMIn)
```

1.2.2 Statistical options and functions

These options and functions are used in some of the analyses that follow

```
#setup for multimodel selection
options(na.action = "na.fail")   #prevent dredge from fitting models to different
datasets

# Function to calculate Root Mean Squared Error from GLMs and GLMMs for calculation of
Coefficients of Variation (CV)
rmse <- function(error) {sqrt(mean(error^2))}
```

2. Background

2.0.1 Relative testes size analysis

Comparing relative testes size (controlling for body size) of spring peepers to ten other frogs in the family Hylidae where females are know to mate multiply (according to Emerson 1997). Data from Appendix 1 in Emerson (1997).

```
dat3 <- dat2[2:11,] #delete spring peeper from the dataset
mod0 <- lm(log10(Testes*1000)~log10(SVL), data=dat3)
newdata = data.frame(SVL=25.2)
Pcrucifer <- predict(mod0, newdata) #predicted value for spring peeper
(predict.g <- 10^Pcrucifer/1000) #convert predicted value to g

##           1
## 0.004082107

predict.g/0.0024 #ratio of predicted to actual value from Emerson (1997)

##        1
## 1.700878
```

3. Materials

3.1 Data analysis

3.1.1 Examining distributions of morphological variables.

```
stat.desc(dat1) #requires pastecs package; descriptive stats for continuous variables

##            Individual Population Lineage      Nsperm    SpHeadLen
## nbr.val            NA         NA      NA  86.0000000 8.600000e+01
## nbr.null           NA         NA      NA   0.0000000 0.000000e+00
## nbr.na             NA         NA      NA   0.0000000 0.000000e+00
## min                NA         NA      NA   3.0000000 1.804000e+01
## max                NA         NA      NA  10.0000000 2.724000e+01
## range              NA         NA      NA   7.0000000 9.200000e+00
## sum                NA         NA      NA 727.0000000 1.908990e+03
## median             NA         NA      NA  10.0000000 2.210000e+01
## mean               NA         NA      NA   8.4534884 2.219756e+01
## SE.mean            NA         NA      NA   0.2571319 1.791646e-01
## CI.mean            NA         NA      NA   0.5112471 3.562272e-01
## var                NA         NA      NA   5.6860465 2.760595e+00
## std.dev            NA         NA      NA   2.3845433 1.661504e+00
## coef.var           NA         NA      NA   0.2820780 7.485075e-02
##               SpTailLen   SpHeadPerim   TOTspermlen     BodyMass          SVL
## nbr.val    86.0000000 8.600000e+01    86.0000000   86.00000000 8.600000e+01
```

```
## nbr.null    0.0000000 0.000000e+00    0.0000000   0.00000000 0.000000e+00
## nbr.na      0.0000000 0.000000e+00    0.0000000   0.00000000 0.000000e+00
## min        11.3500000 3.786000e+01   31.1295000   1.80000000 2.130000e+01
## max        58.0900000 5.585000e+01   80.8864000   3.45000000 3.040000e+01
## range      46.7400000 1.799000e+01   49.7569000   1.65000000 9.100000e+00
## sum      2551.0100000 3.969780e+03 4459.9735192 217.05000000 2.243500e+03
## median     28.0550000 4.597500e+01   49.7071500   2.45000000 2.605000e+01
## mean       29.6629070 4.616023e+01   51.8601572   2.52383721 2.608721e+01
## SE.mean     1.1602353 3.458079e-01    1.1838694   0.03745245 2.450916e-01
## CI.mean     2.3068587 6.875588e-01    2.3538495   0.07446550 4.873078e-01
## var       115.7685573 1.028415e+01  120.5330210   0.12063098 5.166011e+00
## std.dev    10.7595798 3.206891e+00   10.9787532   0.34731972 2.272886e+00
## coef.var    0.3627284 6.947302e-02    0.2116992   0.13761574 8.712645e-02
##             HeadWidth    radioulna        femur        tibia         foot
## nbr.val   86.00000000  86.00000000  86.0000000 8.600000e+01 8.600000e+01
## nbr.null   0.00000000   0.00000000   0.0000000 0.000000e+00 0.000000e+00
## nbr.na     0.00000000   0.00000000   0.0000000 0.000000e+00 0.000000e+00
## min        6.40000000   3.60000000   8.5000000 9.300000e+00 1.240000e+01
## max        9.50000000   7.00000000  13.6000000 1.490000e+01 2.090000e+01
## range      3.10000000   3.40000000   5.1000000 5.600000e+00 8.500000e+00
## sum      691.00000000 473.10000000 926.5000000 1.068350e+03 1.507400e+03
## median     8.05000000   5.50000000  10.5000000 1.235000e+01 1.760000e+01
## mean       8.03488372   5.50116279  10.7732558 1.242267e+01 1.752791e+01
## SE.mean    0.06859480   0.05755493   0.1311156 1.267765e-01 1.717090e-01
## CI.mean    0.13638483   0.11443461   0.2606930 2.520656e-01 3.414036e-01
## var        0.40465116   0.28488098   1.4784528 1.382215e+00 2.535624e+00
## std.dev    0.63612197   0.53374243   1.2159164 1.175676e+00 1.592364e+00
## coef.var   0.07917003   0.09702357   0.1128643 9.463956e-02 9.084737e-02
##                   SMI           PC1          DOY         DOY2     latitude
## nbr.val   86.00000000  8.600000e+01 8.600000e+01 8.600000e+01 8.600000e+01
## nbr.null   0.00000000  0.000000e+00 0.000000e+00 0.000000e+00 0.000000e+00
## nbr.na     0.00000000  0.000000e+00 0.000000e+00 0.000000e+00 0.000000e+00
## min        2.08811533 -3.304874e+00 1.180000e+02 1.392400e+04 4.267162e+01
## max        3.13770665  1.773412e+00 1.370000e+02 1.876900e+04 4.354562e+01
## range      1.04959132  5.078286e+00 1.900000e+01 4.845000e+03 8.740070e-01
## sum      217.47250616 -1.000000e-08 1.097200e+04 1.402718e+06 3.689676e+03
## median     2.53218089  4.588783e-02 1.290000e+02 1.664100e+04 4.272510e+01
## mean       2.52875007 -1.162791e-10 1.275814e+02 1.631067e+04 4.290321e+01
## SE.mean    0.02535679  1.078328e-01 6.293038e-01 1.602913e+02 3.046090e-02
## CI.mean    0.05041608  2.144004e-01 1.251225e+00 3.187020e+02 6.056444e-02
## var        0.05529512  1.000000e+00 3.405800e+01 2.209623e+06 7.979654e-02
## std.dev    0.23514915  1.000000e+00 5.835923e+00 1.486480e+03 2.824828e-01
## coef.var   0.09299027 -8.600000e+09 4.574275e-02 9.113542e-02 6.584188e-03
```

```
##             TotalSperm     Abnormal         noAb     CytoDrop         noCD
## nbr.val      85.000000   85.0000000   85.0000000   85.0000000   85.000000
## nbr.null      0.000000    0.0000000    1.0000000    0.0000000    0.000000
## nbr.na        1.000000    1.0000000    1.0000000    1.0000000    1.000000
## min          27.000000   11.0000000    0.0000000    6.0000000    3.000000
## max         187.000000   97.0000000  100.0000000   91.0000000  116.000000
## range       160.000000   86.0000000  100.0000000   85.0000000  113.000000
## sum        6804.000000 3822.0000000 2982.0000000 3025.0000000 3779.000000
## median       79.000000   44.0000000   32.0000000   33.0000000   42.000000
## mean         80.047059   44.9647059   35.0823529   35.5882353   44.458824
## SE.mean       2.561780    1.8108332    1.9561876    1.8088867    2.157623
## CI.mean       5.094381    3.6010404    3.8900936    3.5971696    4.290670
## var         557.831092  278.7249300  325.2669468  278.1260504  395.703641
## std.dev      23.618448   16.6950571   18.0351586   16.6771116   19.892301
## coef.var      0.295057    0.3712925    0.5140806    0.4686131    0.447432
##                 NoTail         no0t     NoHead         no0H   TwoTails
## nbr.val     85.0000000   85.0000000 85.0000000   85.0000000 85.0000000
## nbr.null     5.0000000    0.0000000 42.0000000    0.0000000 49.0000000
## nbr.na       1.0000000    1.0000000  1.0000000    1.0000000  1.0000000
## min          0.0000000   24.0000000  0.0000000   27.0000000  0.0000000
## max         45.0000000  171.0000000  5.0000000  185.0000000  9.0000000
## range       45.0000000  147.0000000  5.0000000  158.0000000  9.0000000
## sum        746.0000000 6058.0000000 79.0000000 6725.0000000 79.0000000
## median       6.0000000   71.0000000  1.0000000   78.0000000  0.0000000
## mean         8.7764706   71.2705882  0.9294118   79.1176471  0.9294118
## SE.mean      0.8138122    2.5456828  0.1304895    2.5521130  0.1928616
## CI.mean      1.6183548    5.0623694  0.2594927    5.0751565  0.3835264
## var         56.2946779  550.8425770  1.4473389  553.6288515  3.1616246
## std.dev      7.5029779   23.4700357  1.2030540   23.5293190  1.7780958
## coef.var     0.8548969    0.3293089  1.2944252    0.2973966  1.9131410
##                   no2t    TwoHeads         no2H       Other         noOTH
## nbr.val     85.0000000 85.00000000   85.0000000 85.00000000   85.0000000
## nbr.null     0.0000000 83.00000000    0.0000000 79.00000000    0.0000000
## nbr.na       1.0000000  1.00000000    1.0000000  1.00000000    1.0000000
## min         27.0000000  0.00000000   27.0000000  0.00000000   27.0000000
## max        187.0000000  1.00000000  187.0000000  2.00000000  187.0000000
## range      160.0000000  1.00000000  160.0000000  2.00000000  160.0000000
## sum       6725.0000000  2.00000000 6802.0000000  7.00000000 6797.0000000
## median      78.0000000  0.00000000   79.0000000  0.00000000   79.0000000
## mean        79.1176471  0.02352941   80.0235294  0.08235294   79.9647059
## SE.mean      2.5768530  0.01653848    2.5619483  0.03434772    2.5566658
## CI.mean      5.1243548  0.03288857    5.0947151  0.06830421    5.0842102
## var        564.4145658  0.02324930  557.9042017  0.10028011  555.6058824
```

```
## std.dev    23.7574108  0.15247721   23.6199958  0.31667035   23.5712936
## coef.var    0.3002795  6.48028145    0.2951631  3.84528285    0.2947712
##             Pabnormal          Pcd      P0tails      P0heads      P2tails
## nbr.val  85.00000000 85.00000000 85.000000000 85.000000000 8.500000e+01
## nbr.null  0.00000000  0.00000000  5.000000000 42.000000000 4.900000e+01
## nbr.na    1.00000000  1.00000000  1.000000000  1.000000000 1.000000e+00
## min       0.26785714  0.07500000  0.000000000  0.000000000 0.000000e+00
## max       1.00000000  0.96511628  0.569620250  0.074626870 1.428571e-01
## range     0.73214286  0.89011628  0.569620250  0.074626870 1.428571e-01
## sum      48.16085638 37.87726710  9.627206470  1.032945160 1.085882e+00
## median    0.55000000  0.42857143  0.085561500  0.008695650 0.000000e+00
## mean      0.56659831  0.44561491  0.113261253  0.012152296 1.277508e-02
## SE.mean   0.01708451  0.01812698  0.010584756  0.001817056 2.737904e-03
## CI.mean   0.03397441  0.03604748  0.021048949  0.003613416 5.444622e-03
## var       0.02480983  0.02792992  0.009523151  0.000280644 6.371701e-04
## std.dev   0.15751135  0.16712248  0.097586631  0.016752432 2.524223e-02
## coef.var  0.27799475  0.37503791  0.861606500  1.378540466 1.975895e+00
##                P2heads       Pother
## nbr.val  8.500000e+01 8.500000e+01
## nbr.null 8.300000e+01 7.900000e+01
## nbr.na   1.000000e+00 1.000000e+00
## min      0.000000e+00 0.000000e+00
## max      1.298701e-02 1.869159e-02
## range    1.298701e-02 1.869159e-02
## sum      2.533269e-02 7.734177e-02
## median   0.000000e+00 0.000000e+00
## mean     2.980316e-04 9.099032e-04
## SE.mean  2.095508e-04 3.738564e-04
## CI.mean  4.167147e-04 7.434544e-04
## var      3.732480e-06 1.188033e-05
## std.dev  1.931963e-03 3.446786e-03
## coef.var 6.482408e+00 3.788080e+00

#sperm traits
plot(density(dat1$SpTailLen))
```

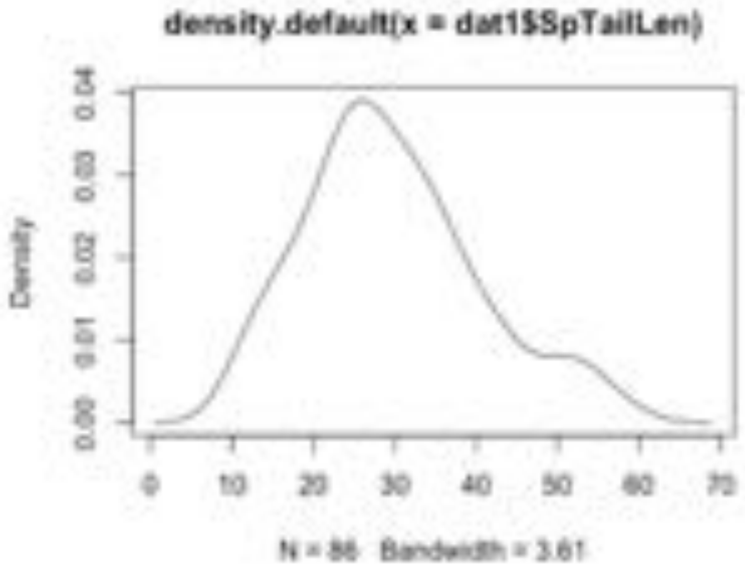


```
plot(density(dat1$SpHeadLen))
```

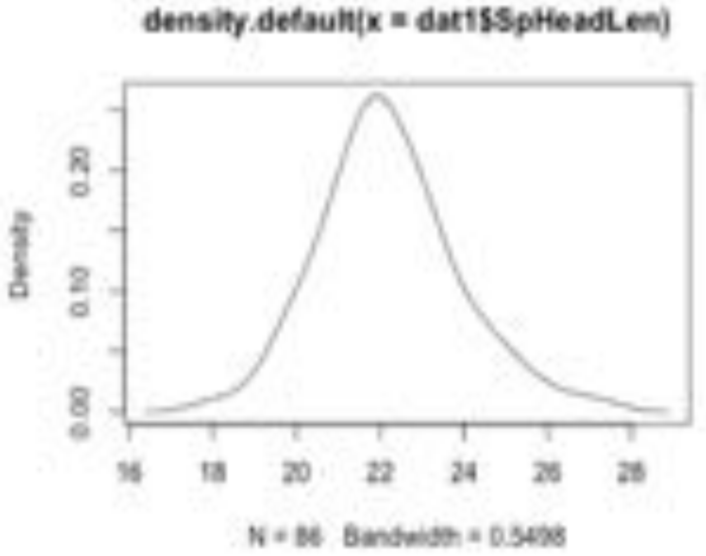


```
#body traits
plot(density(dat1$SVL))
```

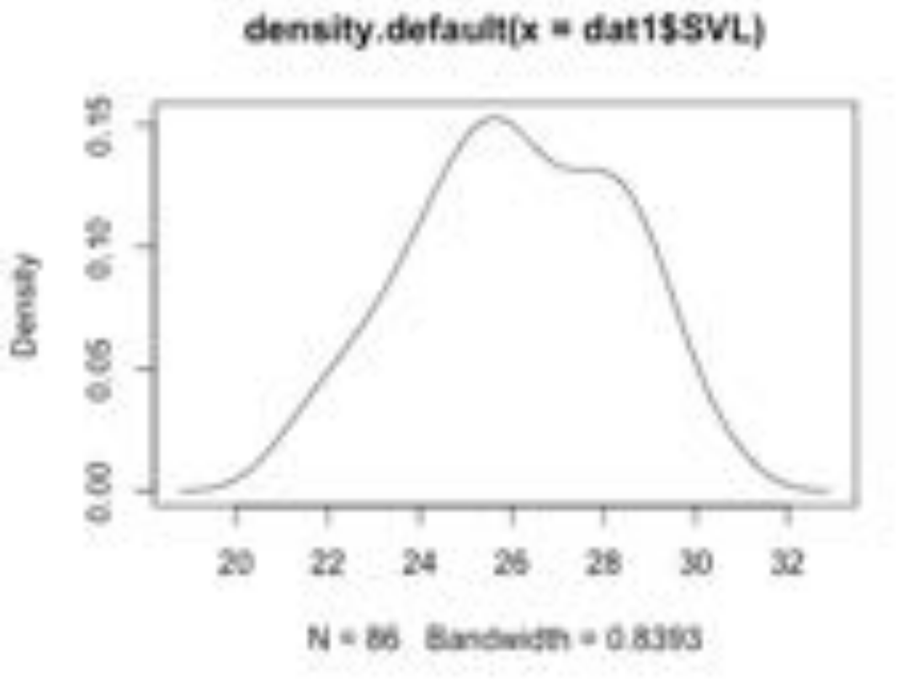


```
plot(density(dat1$BodyMass))
```

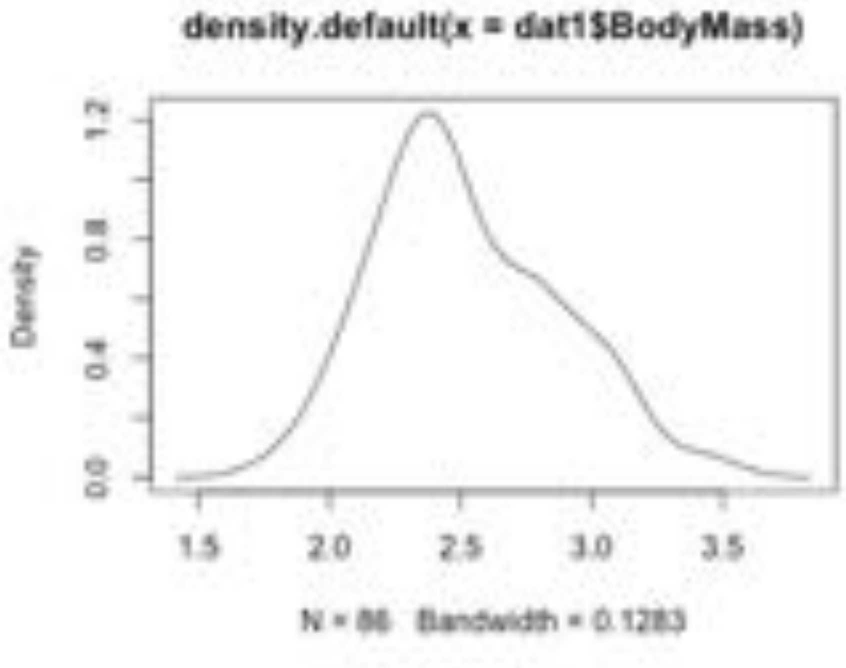

```
#scatterplot matrices
splom(dat1[,5:8]) #sperm traits
```

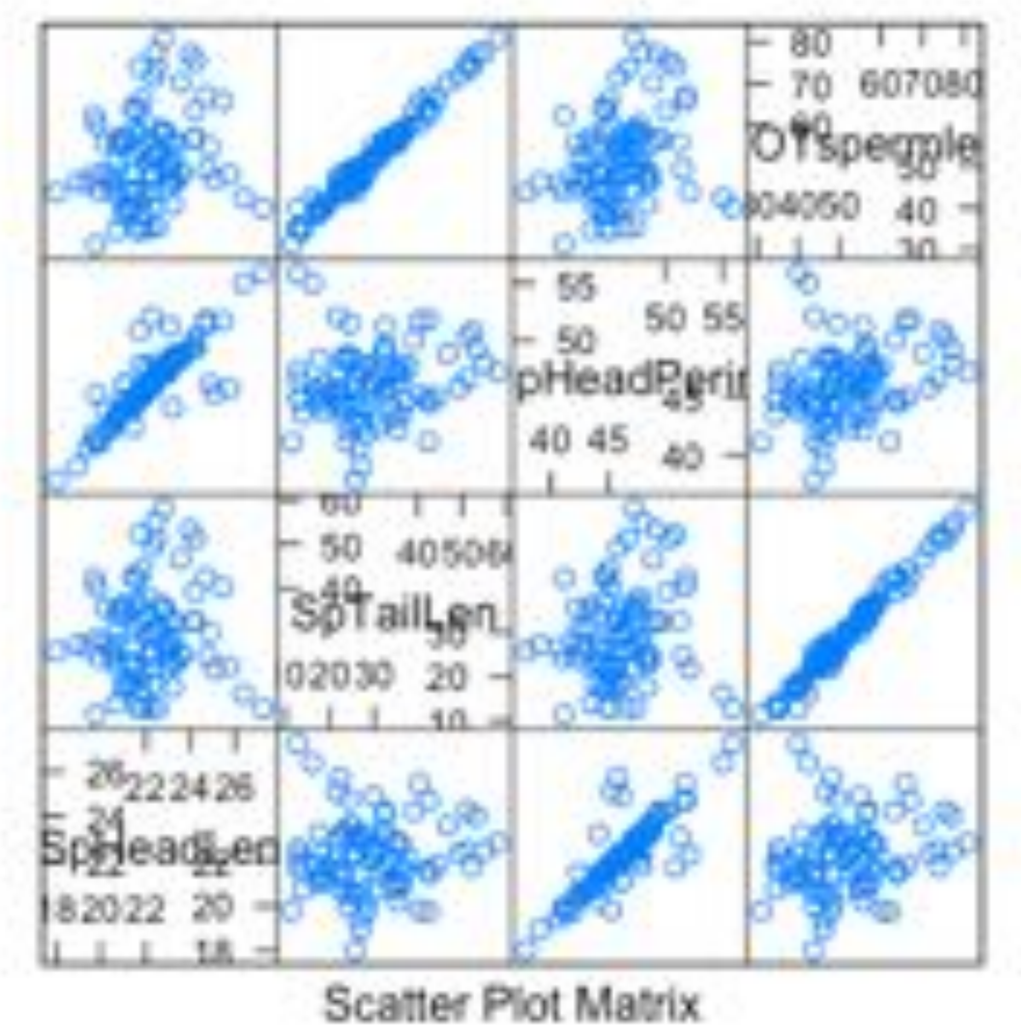

```
splom(dat1[,9:16]) #body size traits
```

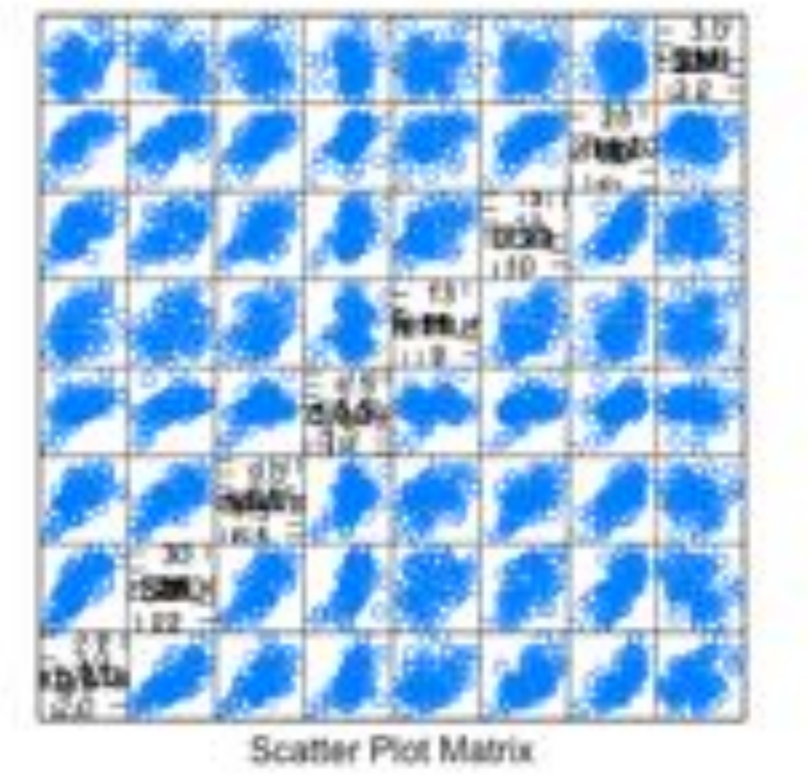

```
splom(dat1[,22:29]) #number of abnormalities
```

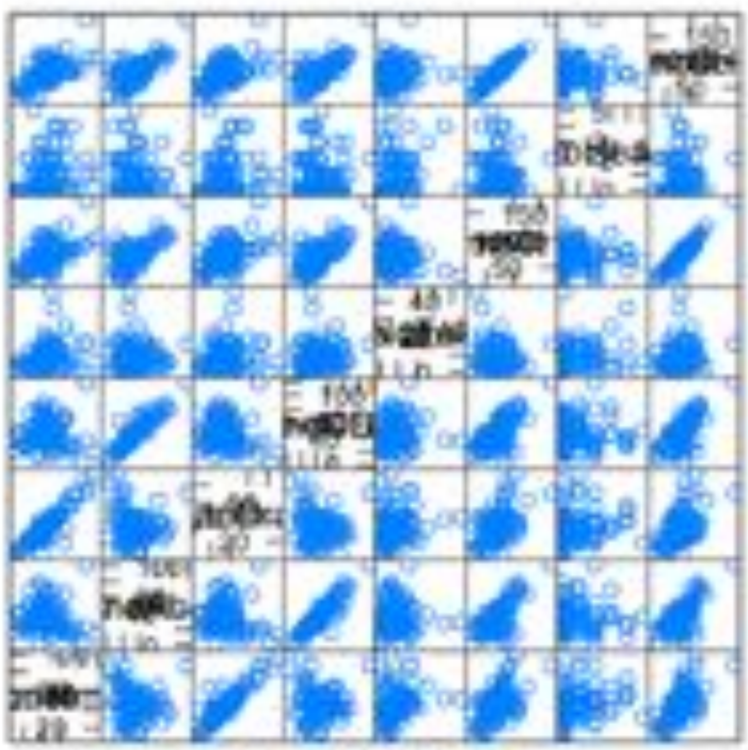

4. Results

4.1 Frog body size and condition

Calculate PCA for body size, extracting PC scores for two axes using the varimax rotation; and SMI based on Peig and Green (2009). This is the code used to calculate PC1 and SMI which are already in the *PEEPERS.csv* dataset. PCA details and the correlation between SMI and PC1 are reported in the main text of the paper.

```
mod1 <- principal(dat1[,10:15], nfactors=2, rotate="varimax")
mod1

## Principal Components Analysis
## Call: principal(r = dat1[, 10:15], nfactors = 2, rotate = "varimax")
## Standardized loadings (pattern matrix) based upon correlation matrix
##            PC1  PC2   h2    u2 com
## SVL       0.84 0.18 0.75 0.253 1.1
## HeadWidth 0.75 0.38 0.71 0.290 1.5
## radioulna 0.79 0.03 0.62 0.376 1.0
## femur     0.12 0.94 0.90 0.096 1.0
## tibia     0.60 0.60 0.73 0.271 2.0
## foot      0.79 0.36 0.76 0.239 1.4
##
```

```
##                        PC1  PC2
## SS loadings           2.91 1.56
## Proportion Var        0.49 0.26
## Cumulative Var        0.49 0.75
## Proportion Explained  0.65 0.35
## Cumulative Proportion 0.65 1.00
##
## Mean item complexity =  1.3
## Test of the hypothesis that 2 components are sufficient.
##
## The root mean square of the residuals (RMSR) is  0.08
##  with the empirical chi square  18  with prob <  0.0012
##
## Fit based upon off diagonal values = 0.98
```

```
dat1$PC2 <- mod1$scores[,1]
```

```
(mod2 <- lmodel2(log(BodyMass)~log(SVL), "relative", "relative", data=dat1)) #bSMA is
slope of SMA model 2 regression
```

```
## No permutation test will be performed
```

```
##
## Model II regression
##
## Call: lmodel2(formula = log(BodyMass) ~ log(SVL), data = dat1,
## range.y = "relative", range.x = "relative")
##
## n = 86   r = 0.7669807   r-square = 0.5882595
## Parametric P-values:   2-tailed = 7.318944e-18    1-tailed = 3.659472e-18
## Angle between the two OLS regression lines = 13.77913 degrees
##
## Regression results
##   Method Intercept    Slope Angle (degrees) P-perm (1-tailed)
## 1    OLS -2.929790 1.180731        49.73764                NA
## 2     MA -4.739297 1.736198        60.05930                NA
## 3    SMA -4.098374 1.539453        56.99300                NA
## 4    RMA -5.477388 1.962772        63.00190                NA
##
## Confidence intervals
##   Method 2.5%-Intercept 97.5%-Intercept 2.5%-Slope 97.5%-Slope
## 1    OLS      -3.628260       -2.231321  0.9663984    1.395064
## 2     MA      -5.932278       -3.833306  1.4580847    2.102410
## 3    SMA      -4.844962       -3.448530  1.3399695    1.768635
## 4    RMA      -6.886067       -4.489589  1.6595452    2.395196
```

```
##
## Eigenvalues: 0.02377666 0.002494092
##
## H statistic used for computing C.I. of MA: 0.006163611

dat1$SMI <- dat1$BodyMass * (mean(dat1$SVL)/dat1$SVL)^mod2$regression.result$Slope[3]

#correlation between size and condition
cor.test(~SMI+PC1, data=dat1)

##
##  Pearson's product-moment correlation
##
## data:  SMI and PC1
## t = -1.3937, df = 84, p-value = 0.1671
## alternative hypothesis: true correlation is not equal to 0
## 95 percent confidence interval:
##  -0.35103347  0.06356021
## sample estimates:
##        cor
## -0.1503397

mod3 <- lm(SMI~PC1+DOY+DOY2, data=dat1)

m3 <- dredge(mod3)

## Fixed term is "(Intercept)"

subset (m3,delta<2.5)

## Global model call: lm(formula = SMI ~ PC1 + DOY + DOY2, data = dat1)
## ---
## Model selection table
##   (Intrc)       DOY       DOY2      PC1 df logLik AICc delta weight
## 8 -17.110  0.316100 -1.269e-03 -0.04412  5  6.660 -2.6  0.00  0.148
## 7   2.982           -2.797e-05 -0.03710  4  5.472 -2.4  0.12  0.139
## 3   2.965           -2.689e-05           3  4.354 -2.4  0.15  0.137
## 6   3.418 -0.006989            -0.03691  4  5.419 -2.3  0.23  0.132
## 2   3.386 -0.006740                      3  4.313 -2.3  0.24  0.131
## 1   2.526                                2  3.090 -2.0  0.54  0.113
## 5   2.526                      -0.03530  3  4.073 -1.9  0.72  0.103
## 4 -12.900  0.249600 -1.007e-03           4  5.097 -1.7  0.87  0.096
## Models ranked by AICc(x)

Anova(mod3)

## Anova Table (Type II tests)
##
```

```
## Response: SMI
##           Sum Sq Df F value  Pr(>F)
## PC1       0.1596  1  3.0353 0.08522 .
## DOY       0.1208  1  2.2974 0.13343
## DOY2      0.1263  1  2.4011 0.12510
## Residuals 4.3128 82
## ---
## Signif. codes:  0 '***' 0.001 '**' 0.01 '*' 0.05 '.' 0.1 ' ' 1
```

```
summary(mod3)
```

```
##
## Call:
## lm(formula = SMI ~ PC1 + DOY + DOY2, data = dat1)
##
## Residuals:
##      Min       1Q   Median       3Q      Max
## -0.48404 -0.13130 -0.00167  0.15476  0.55584
##
## Coefficients:
##               Estimate Std. Error t value Pr(>|t|)
## (Intercept) -1.711e+01  1.326e+01  -1.290   0.2005
## PC1         -4.412e-02  2.533e-02  -1.742   0.0852 .
## DOY          3.161e-01  2.085e-01   1.516   0.1334
## DOY2        -1.269e-03  8.189e-04  -1.550   0.1251
## ---
## Signif. codes:  0 '***' 0.001 '**' 0.01 '*' 0.05 '.' 0.1 ' ' 1
##
## Residual standard error: 0.2293 on 82 degrees of freedom
## Multiple R-squared:  0.07968,    Adjusted R-squared:  0.046
## F-statistic: 2.366 on 3 and 82 DF,  p-value: 0.07686
```

4.2 Sperm size

4.2.1 Analyses of the seasonal variation in sperm size traits

Some of these models and coefficients of variation (CV) are reported in the text; mod5a, mod7a, and mod8a are summarized in Table 1; other models here are to check for significant effects.

```
#total sperm length
mod4<- lm(TOTspermlen~DOY, data=dat1) #fit linear model without controlling for body
size and condition
plot(mod4)
```

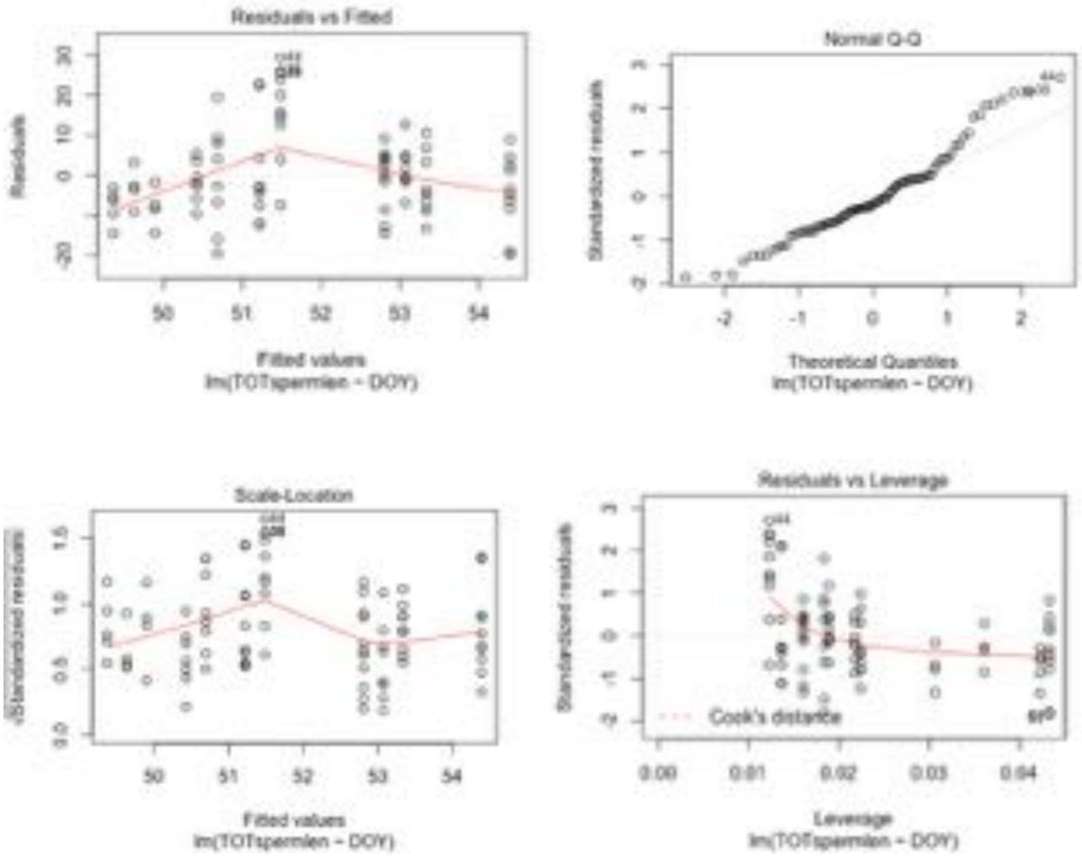


```
plot(TOTspermlen~DOY, data=dat1)
abline(mod4) #data are clearly nonlinear
```

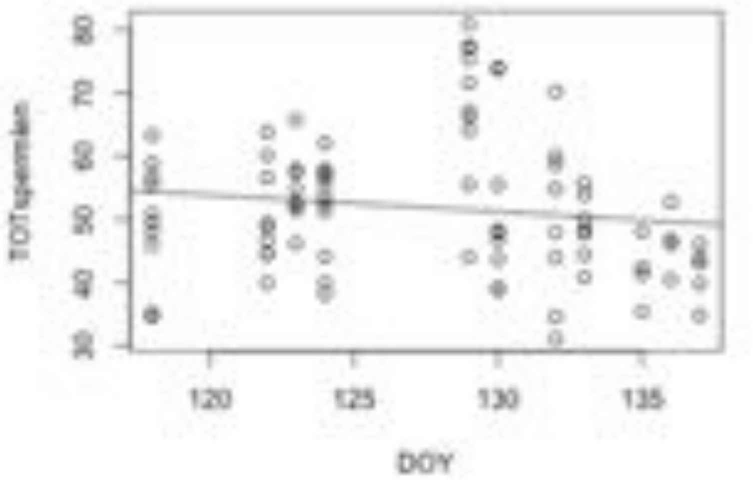


```
mod5<- lm(TOTspermlen~DOY2+DOY+PC1+SMI, data=dat1) #test quadratic model
m5 <- dredge(mod5)

## Fixed term is "(Intercept)"

subset (m5,delta<2.5)

## Global model call: lm(formula = TOTspermlen ~ DOY2 + DOY + PC1 + SMI, data = dat1)
## ---
## Model selection table
##    (Intrc)   DOY    DOY2   PC1    SMI df   logLik  AICc delta weight
## 12   -2235 36.72 -0.1455       -9.737  5 -317.604 646.0  0.00  0.364
## 16   -2071 34.07 -0.1350 1.550 -8.483  6 -316.586 646.2  0.28  0.317
## 8    -1926 31.39 -0.1242 1.925         5 -318.236 647.2  1.26  0.193
## 4    -2110 34.29 -0.1357               4 -319.792 648.1  2.12  0.126
## Models ranked by AICc(x)

mod5a<- lm(TOTspermlen~DOY2+DOY+SMI, data=dat1) #fit best model
(m5a <- Anova(mod5a))

## Anova Table (Type II tests)
##
## Response: TOTspermlen
##           Sum Sq Df F value    Pr(>F)
## DOY2      1687.4  1 17.0308 8.783e-05 ***
## DOY       1658.3  1 16.7373 9.983e-05 ***
## SMI        424.0  1  4.2796   0.04172 *
## Residuals 8124.3 82
## ---
## Signif. codes:  0 '***' 0.001 '**' 0.01 '*' 0.05 '.' 0.1 ' ' 1
```

```
summary(mod5a)

##
## Call:
## lm(formula = TOTspermlen ~ DOY2 + DOY + SMI, data = dat1)
##
## Residuals:
##      Min       1Q   Median       3Q      Max
## -19.4864  -6.7173  -0.2266   6.0266  23.5602
##
## Coefficients:
##               Estimate Std. Error t value Pr(>|t|)
## (Intercept) -2.235e+03  5.690e+02  -3.929 0.000177 ***
## DOY2        -1.455e-01  3.526e-02  -4.127 8.78e-05 ***
## DOY          3.672e+01  8.976e+00   4.091 9.98e-05 ***
## SMI         -9.737e+00  4.707e+00  -2.069 0.041722 *
## ---
## Signif. codes:  0 '***' 0.001 '**' 0.01 '*' 0.05 '.' 0.1 ' ' 1
##
## Residual standard error: 9.954 on 82 degrees of freedom
## Multiple R-squared:  0.207,  Adjusted R-squared:  0.178
## F-statistic: 7.136 on 3 and 82 DF,  p-value: 0.0002565

confint(mod5a)

##                     2.5 %        97.5 %
## (Intercept) -3367.171050 -1.103503e+03
## DOY2            -0.215626 -7.535845e-02
## DOY             18.865161  5.457611e+01
## SMI            -19.099832 -3.737304e-01
plot(mod5a)
```

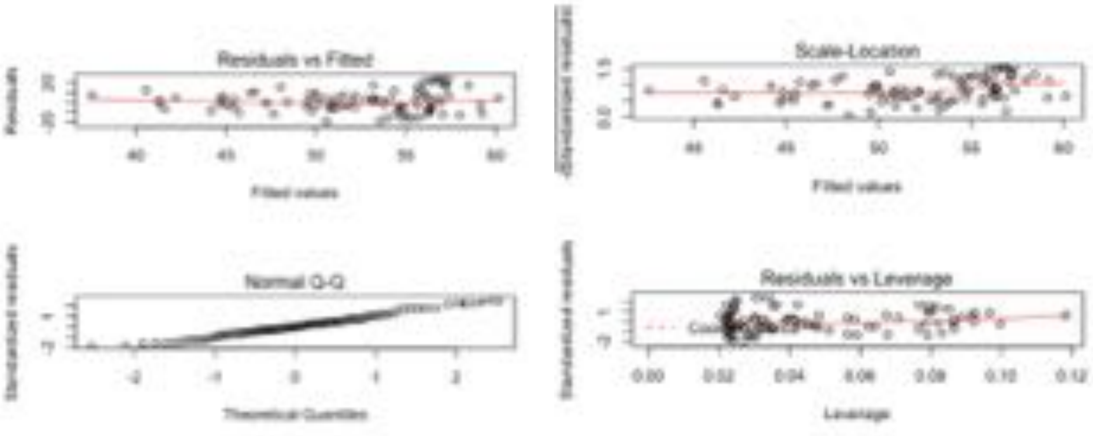

```
#proportions of variance explained
m5a[3,1]/sum(m5a[,1]) # by SMI

## [1] 0.03564929

sum(m5a[1:2,1])/sum(m5a[,1]) #by date and dat^2

## [1] 0.2812889

m5a[4,1]/sum(m5a[,1]) #variance remaining unexplained

## [1] 0.6830618

(cv5a <-(rmse(mod5a$residuals)/mean(dat1$TOTspermlen)) *100) #coeff of variation,
controlling for date as a quadratic predictor, and body condition

## [1] 18.74175

#fit model to plot in Fig 2
mod5b<- lm(TOTspermlen~DOY2+DOY, data=dat1)
summary(mod5b)

##
## Call:
## lm(formula = TOTspermlen ~ DOY2 + DOY, data = dat1)
##
## Residuals:
##      Min       1Q   Median       3Q      Max
## -21.1777  -6.6644  -0.2522   5.8423  25.2040
##
## Coefficients:
##               Estimate Std. Error t value Pr(>|t|)
## (Intercept) -2.110e+03  5.768e+02  -3.658 0.000445 ***
## DOY2        -1.357e-01  3.562e-02  -3.810 0.000266 ***
## DOY          3.429e+01  9.073e+00   3.780 0.000295 ***
## ---
## Signif. codes:  0 '***' 0.001 '**' 0.01 '*' 0.05 '.' 0.1 ' ' 1
##
## Residual standard error: 10.15 on 83 degrees of freedom
## Multiple R-squared:  0.1656, Adjusted R-squared:  0.1455
## F-statistic: 8.238 on 2 and 83 DF,  p-value: 0.0005448

#partial regression plot in Fig 3
resY  <- residuals(lm(TOTspermlen~DOY2+DOY, data=dat1))
resX <- residuals(lm(SMI~DOY2+DOY, data=dat1))
xyplot(resY~resX)
```

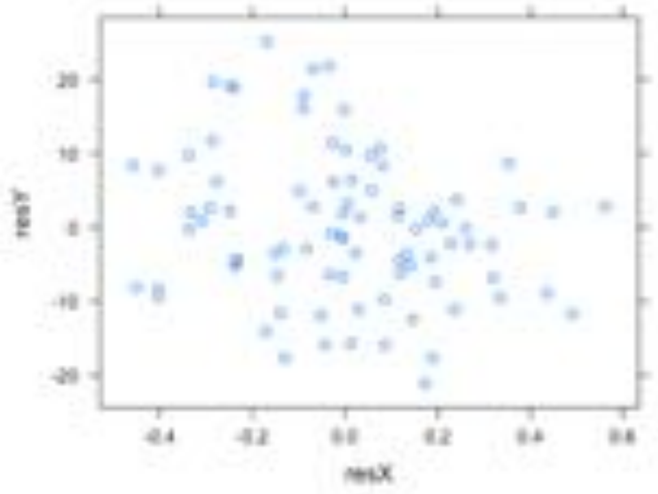


```
#flagellum length
mod6<- lm(SpTailLen~DOY2+DOY+PC1+SMI, data=dat1) #test quadratic model
m6 <- dredge(mod6)

## Fixed term is "(Intercept)"

subset (m6,delta<2.5)

## Global model call: lm(formula = SpTailLen ~ DOY2 + DOY + PC1 + SMI, data = dat1)
## ---
## Model selection table
##     (Intrc)   DOY    DOY2   PC1    SMI df   logLik  AICc delta weight
## 12    -2288 37.13 -0.1468        -9.563  5 -315.810 642.4  0.00  0.414
## 16    -2148 34.86 -0.1378 1.327 -8.489  6 -315.035 643.1  0.76  0.283
## 8     -2003 32.17 -0.1271 1.702         5 -316.746 644.2  1.87  0.162
## 4     -2165 34.74 -0.1372                4 -318.010 644.5  2.14  0.142
## Models ranked by AICc(x)

mod6a<- lm(SpTailLen~DOY2+DOY+SMI, data=dat1) #best-fit model
plot(mod6a)
```

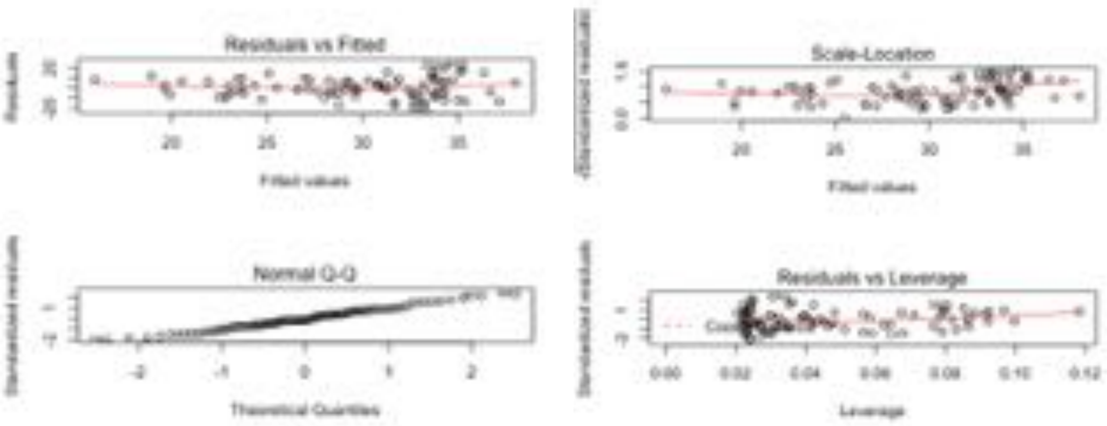

```
summary(mod6a)

##
## Call:
## lm(formula = SpTailLen ~ DOY2 + DOY + SMI, data = dat1)
##
## Residuals:
##     Min      1Q  Median      3Q     Max
## -19.919  -6.736  -1.312   6.976  22.847
##
## Coefficients:
##               Estimate Std. Error t value Pr(>|t|)
## (Intercept) -2.288e+03  5.572e+02  -4.107 9.44e-05 ***
## DOY2        -1.468e-01  3.453e-02  -4.252 5.58e-05 ***
## DOY          3.713e+01  8.790e+00   4.224 6.19e-05 ***
## SMI         -9.563e+00  4.609e+00  -2.075   0.0412 *
## ---
## Signif. codes:  0 '***' 0.001 '**' 0.01 '*' 0.05 '.' 0.1 ' ' 1
##
## Residual standard error: 9.748 on 82 degrees of freedom
## Multiple R-squared:  0.2081, Adjusted R-squared:  0.1792
## F-statistic: 7.184 on 3 and 82 DF,  p-value: 0.0002428

confint(mod6a)

##                     2.5 %        97.5 %
## (Intercept) -3396.7131381 -1.179785e+03
## DOY2           -0.2155008 -7.812957e-02
## DOY            19.6402832  5.461387e+01
## SMI           -18.7323892 -3.929475e-01

Anova(mod6a)

## Anova Table (Type II tests)
##
## Response: SpTailLen
##           Sum Sq Df F value    Pr(>F)
## DOY2      1718.2  1 18.0809 5.576e-05 ***
## DOY       1695.2  1 17.8390 6.188e-05 ***
## SMI        409.0  1  4.3038   0.04116 *
## Residuals 7792.3 82
## ---
## Signif. codes:  0 '***' 0.001 '**' 0.01 '*' 0.05 '.' 0.1 ' ' 1
```

```
(cv6a <-rmse(mod6a$residuals)/mean(dat1$SpTailLen) *100) #coeff of variation,
controlling for date, body size and body condition

## [1] 32.08995

#sperm head length
mod7<- lm(SpHeadLen~DOY2+DOY+PC1+SMI, data=dat1) #test quadratic model
m7 <- dredge(mod7)

## Fixed term is "(Intercept)"

subset (m7,delta<2.5)

## Global model call: lm(formula = SpHeadLen ~ DOY2 + DOY + PC1 + SMI, data = dat1)
## ---
## Model selection table
##    (Intrc)      DOY       DOY2    PC1     SMI df   logLik  AICc delta
## 2    30.93 -0.06841                            3 -162.633 331.6  0.00
## 3    26.57          -0.0002679                 3 -162.647 331.6  0.03
## 6    30.75 -0.06701            0.2067          4 -161.922 332.3  0.78
## 7    26.47          -0.0002619 0.2053          4 -161.945 332.4  0.83
## 4    54.95 -0.44660  0.0014850                 4 -162.598 333.7  2.13
## 10   31.59 -0.06974                   -0.1975  4 -162.598 333.7  2.13
## 11   27.16          -0.0002733        -0.2016  4 -162.611 333.7  2.16
##    weight
## 2   0.230
## 3   0.227
## 6   0.156
## 7   0.152
## 4   0.079
## 10  0.079
## 11  0.078
## Models ranked by AICc(x)

mod7a<- lm(SpHeadLen~DOY, data=dat1) #fit best model
summary(mod7a)

##
## Call:
## lm(formula = SpHeadLen ~ DOY, data = dat1)
##
## Residuals:
##     Min      1Q  Median      3Q     Max
## -3.9921 -1.0171 -0.1031  0.7889  4.7974
##
## Coefficients:
##             Estimate Std. Error t value Pr(>|t|)
```

```
## (Intercept) 30.92521    3.85101   8.030 5.27e-12 ***
## DOY         -0.06841    0.03015  -2.269   0.0259 *
## ---
## Signif. codes:  0 '***' 0.001 '**' 0.01 '*' 0.05 '.' 0.1 ' ' 1
##
## Residual standard error: 1.622 on 84 degrees of freedom
## Multiple R-squared:  0.05773,    Adjusted R-squared:  0.04652
## F-statistic: 5.147 on 1 and 84 DF,  p-value: 0.02585
```

```
confint(mod7a)
```

```
##                  2.5 %       97.5 %
## (Intercept) 23.2670568 38.583369639
## DOY         -0.1283722 -0.008444845
```

```
plot(mod7a)
```

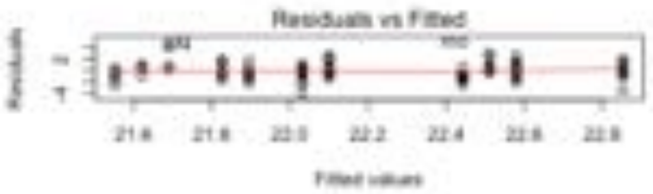

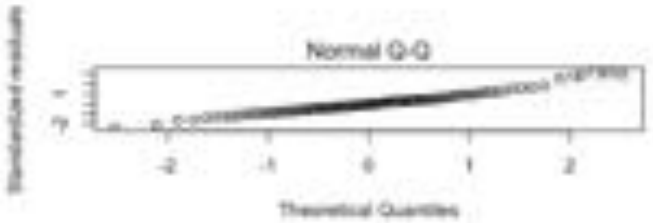

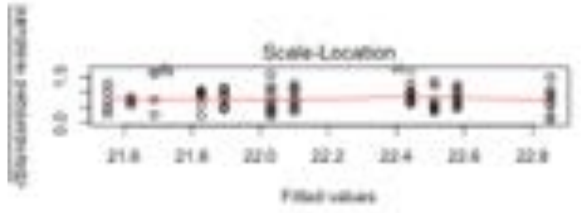

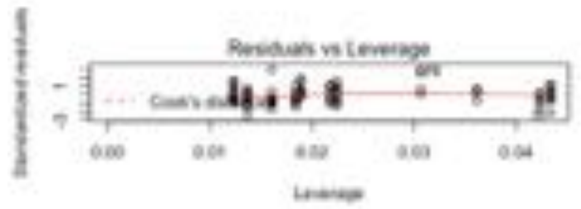


```
(cv7a <-(rmse(mod7a$residuals)/mean(dat1$SpHeadLen)) *100) #coeff of variation,
controlling for date
```

```
## [1] 7.223422
```

```
#sperm head perimeter
mod8<- lm(SpHeadPerim~DOY2+DOY+PC1+SMI, data=dat1) #test quadratic model
m8 <- dredge(mod8)

## Fixed term is "(Intercept)"

subset (m8,delta<2.5)

## Global model call: lm(formula = SpHeadPerim ~ DOY2 + DOY + PC1 + SMI, data = dat1)
## ---
## Model selection table
##    (Intrc)     DOY       DOY2    PC1    SMI df   logLik  AICc delta weight
## 7    53.60          -0.0004562 0.5077         4 -218.497 445.5  0.00  0.119
## 6    60.96 -0.1160             0.5104         4 -218.504 445.5  0.02  0.118
## 3    53.84          -0.0004710                3 -219.642 445.6  0.09  0.114
## 2    61.40 -0.1194                            3 -219.661 445.6  0.13  0.112
## 11   59.64          -0.0005235        -1.954  4 -218.735 446.0  0.48  0.094
## 10   67.97 -0.1325                    -1.942  4 -218.765 446.0  0.54  0.091
## 15   58.51          -0.0005023 0.4467 -1.645  5 -217.855 446.5  0.97  0.073
## 14   66.54 -0.1274             0.4501 -1.632  5 -217.873 446.5  1.01  0.072
## 5    46.16                     0.5370         3 -220.519 447.3  1.84  0.047
## 4   -22.29  1.1980 -0.0051730                 4 -219.549 447.6  2.10  0.042
## 1    46.16                                    2 -221.742 447.6  2.14  0.041
## 8    25.17  0.4473 -0.0022120 0.4978          5 -218.484 447.7  2.23  0.039
## 12  -49.02  1.7150 -0.0072580         -2.072  5 -218.543 447.8  2.35  0.037
## Models ranked by AICc(x)

mod8a<- lm(SpHeadPerim~DOY2+PC1, data=dat1) #fit best model
summary(mod8a)

##
## Call:
## lm(formula = SpHeadPerim ~ DOY2 + PC1, data = dat1)
##
## Residuals:
##     Min      1Q  Median      3Q     Max
## -7.7238 -1.9264 -0.2018  1.5343  9.9474
##
## Coefficients:
##               Estimate Std. Error t value Pr(>|t|)
## (Intercept) 53.6017720  3.7380859  14.339   <2e-16 ***
## DOY2        -0.0004562  0.0002282  -1.999   0.0489 *
## PC1          0.5077483  0.3392848   1.497   0.1383
## ---
## Signif. codes:  0 '***' 0.001 '**' 0.01 '*' 0.05 '.' 0.1 ' ' 1
##
```

```
## Residual standard error: 3.125 on 83 degrees of freedom
## Multiple R-squared:  0.07268,    Adjusted R-squared:  0.05034
## F-statistic: 3.253 on 2 and 83 DF,  p-value: 0.04365

confint(mod8a)

##                     2.5 %        97.5 %
## (Intercept) 46.1668693816  6.103667e+01
## DOY2        -0.0009102118 -2.262988e-06
## PC1         -0.1670755923  1.182572e+00

plot(mod8a)
```

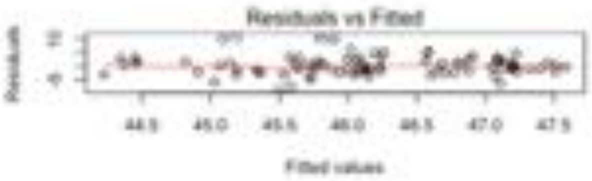

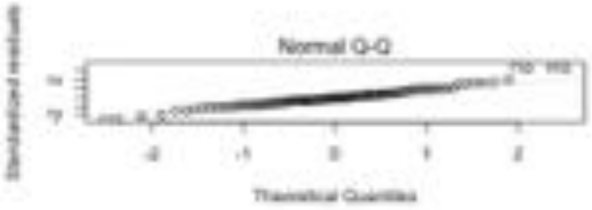

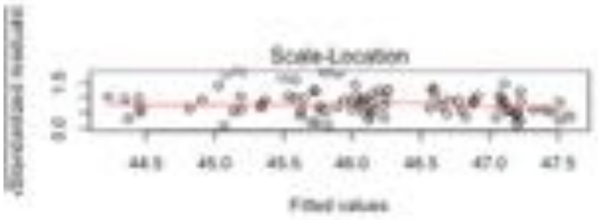

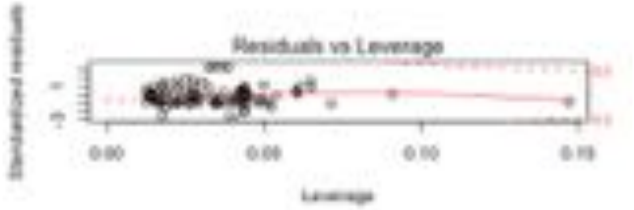


```
(cv8a <-(rmse(mod8a$residuals)/mean(dat1$SpHeadPerim) *100)) #coeff of variation, controlling for date^2, body size

## [1] 6.651052

#is sperm head length corr with sperm tail?
mod9<- lm(SpHeadLen~SpTailLen+DOY2+DOY+PC1+SMI, data=dat1) #test quadratic model
m9 <- dredge(mod9)
```

```
## Fixed term is "(Intercept)"
subset (m9,delta<2.5)

## Global model call: lm(formula = SpHeadLen ~ SpTailLen + DOY2 + DOY + PC1 + SMI,
##     data = dat1)
## ---
## Model selection table
##    (Intrc)      DOY       DOY2    PC1      SMI    SpTlL df   logLik  AICc
## 2    30.93 -0.06841                                   3 -162.633 331.6
## 3    26.57          -0.0002679                         3 -162.647 331.6
## 6    30.75 -0.06701            0.2067                  4 -161.922 332.3
## 7    26.47          -0.0002619 0.2053                  4 -161.945 332.4
## 18   30.66 -0.06747                          0.004799  4 -162.589 333.7
## 4    54.95 -0.44660  0.0014850                         4 -162.598 333.7
## 10   31.59 -0.06974                   -0.1975          4 -162.598 333.7
## 19   26.37          -0.0002641                0.004512  4 -162.608 333.7
## 11   27.16          -0.0002733        -0.2016          4 -162.611 333.7
##    delta weight
## 2   0.00  0.198
## 3   0.03  0.196
## 6   0.78  0.134
## 7   0.83  0.131
## 18  2.11  0.069
## 4   2.13  0.068
## 10  2.13  0.068
## 19  2.15  0.068
## 11  2.16  0.067
## Models ranked by AICc(x)
Anova (mod9)

## Anova Table (Type II tests)
##
## Response: SpHeadLen
##            Sum Sq Df F value Pr(>F)
## SpTailLen   0.137  1  0.0506 0.8226
## DOY2        0.741  1  0.2736 0.6024
## DOY         0.861  1  0.3178 0.5745
## PC1         3.670  1  1.3547 0.2479
## SMI         0.007  1  0.0027 0.9584
## Residuals 216.719 80

#is sperm head perimeter corr with sperm tail?
mod10<- lm(SpHeadPerim~SpTailLen+DOY2+DOY+PC1+SMI, data=dat1) #test quadratic model
m10 <- dredge(mod10)
```

```
## Fixed term is "(Intercept)"
```

```
subset (m10,delta<2.5)
```

```
## Global model call: lm(formula = SpHeadPerim ~ SpTailLen + DOY2 + DOY + PC1 + SMI,
##     data = dat1)
## ---
## Model selection table
##    (Intrc)     DOY       DOY2    PC1    SMI   SpTlL df   logLik  AICc
## 7    53.60          -0.0004562 0.5077                4 -218.497 445.5
## 6    60.96 -0.1160             0.5104                4 -218.504 445.5
## 3    53.84          -0.0004710                       3 -219.642 445.6
## 2    61.40 -0.1194                                   3 -219.661 445.6
## 11   59.64          -0.0005235        -1.954         4 -218.735 446.0
## 10   67.97 -0.1325                    -1.942         4 -218.765 446.0
## 18   59.35 -0.1121                           0.03737  4 -218.954 446.4
## 19   52.25          -0.0004404               0.03687  4 -218.955 446.4
## 15   58.51          -0.0005023 0.4467 -1.645         5 -217.855 446.5
## 14   66.54 -0.1274             0.4501 -1.632         5 -217.873 446.5
## 22   59.48 -0.1109             0.4425        0.02801  5 -218.119 447.0
## 23   52.44          -0.0004353 0.4412        0.02755  5 -218.124 447.0
## 27   57.64          -0.0004918        -1.736 0.03116  5 -218.246 447.2
## 26   65.49 -0.1248                    -1.722 0.03179  5 -218.256 447.3
## 5    46.16                     0.5370                3 -220.519 447.3
## 4   -22.29  1.1980 -0.0051730                        4 -219.549 447.6
## 1    46.16                                           2 -221.742 447.6
## 8    25.17  0.4473 -0.0022120 0.4978                 5 -218.484 447.7
## 12  -49.02  1.7150 -0.0072580        -2.072          5 -218.543 447.8
## 17   44.86                                   0.04383  3 -220.802 447.9
##    delta weight
## 7   0.00  0.085
## 6   0.02  0.085
## 3   0.09  0.082
## 2   0.13  0.080
## 11  0.48  0.067
## 10  0.54  0.065
## 18  0.91  0.054
## 19  0.92  0.054
## 15  0.97  0.052
## 14  1.01  0.052
## 22  1.50  0.040
## 23  1.51  0.040
## 27  1.75  0.035
## 26  1.77  0.035
```

```
## 5   1.84  0.034
## 4   2.10  0.030
## 1   2.14  0.029
## 8   2.23  0.028
## 12  2.35  0.026
## 17  2.41  0.026
## Models ranked by AICc(x)
```

```
Anova(mod10)
```

```
## Anova Table (Type II tests)
##
## Response: SpHeadPerim
##            Sum Sq Df F value Pr(>F)
## SpTailLen   3.92  1  0.3948 0.5316
## DOY2        0.11  1  0.0108 0.9176
## DOY         0.04  1  0.0042 0.9482
## PC1        11.90  1  1.1993 0.2767
## SMI         9.82  1  0.9897 0.3228
## Residuals 793.53 80
```

```
#correlation between sperm tail and total length
cor.test(dat1$TOTspermlen,dat1$SpTailLen)
```

```
##
##  Pearson's product-moment correlation
##
## data:  dat1$TOTspermlen and dat1$SpTailLen
## t = 59.976, df = 84, p-value < 2.2e-16
## alternative hypothesis: true correlation is not equal to 0
## 95 percent confidence interval:
##  0.9824089 0.9925222
## sample estimates:
##       cor
## 0.9885246
```

4.3 Sperm abnormalities

Analyses for Table 2, with a test of the effect of SMI on the proportion of sperm that had abnormalities reported in the text

```
dat3<-subset(dat1, Individual != "11-123") #create new dataset, removing individuals where abnormalities not scored
```

```
stat.desc(dat3) #descriptive statistics of the reduced dataset
```

```
##          Individual Population Lineage      Nsperm    SpHeadLen
## nbr.val          NA         NA      NA  85.0000000 8.500000e+01
```

```
## nbr.null         NA         NA      NA   0.0000000 0.000000e+00
## nbr.na           NA         NA      NA   0.0000000 0.000000e+00
## min              NA         NA      NA   3.0000000 1.804000e+01
## max              NA         NA      NA  10.0000000 2.724000e+01
## range            NA         NA      NA   7.0000000 9.200000e+00
## sum              NA         NA      NA 722.0000000 1.885910e+03
## median           NA         NA      NA  10.0000000 2.210000e+01
## mean             NA         NA      NA   8.4941176 2.218718e+01
## SE.mean          NA         NA      NA   0.2569066 1.809803e-01
## CI.mean          NA         NA      NA   0.5108869 3.598992e-01
## var              NA         NA      NA   5.6100840 2.784080e+00
## std.dev          NA         NA      NA   2.3685616 1.668556e+00
## coef.var         NA         NA      NA   0.2788473 7.520363e-02
##             SpTailLen  SpHeadPerim  TOTspermlen     BodyMass          SVL
## nbr.val    85.0000000 8.500000e+01   85.0000000  85.00000000   85.0000000
## nbr.null    0.0000000 0.000000e+00    0.0000000   0.00000000    0.0000000
## nbr.na      0.0000000 0.000000e+00    0.0000000   0.00000000    0.0000000
## min        11.3500000 3.786000e+01   31.1295000   1.80000000   21.3000000
## max        58.0900000 5.585000e+01   80.8864000   3.45000000   30.4000000
## range      46.7400000 1.799000e+01   49.7569000   1.65000000    9.1000000
## sum      2524.1700000 3.922420e+03 4410.0487192 214.35000000 2215.8000000
## median     28.1000000 4.588000e+01   49.4895000   2.45000000   26.0000000
## mean       29.6961176 4.614612e+01   51.8829261   2.52176471   26.0682353
## SE.mean     1.1734854 3.496089e-01    1.1976586   0.03783762    0.2472479
## CI.mean     2.3336043 6.952356e-01    2.3816755   0.07524426    0.4916797
## var       117.0507717 1.038924e+01  121.9228227   0.12169328    5.1961814
## std.dev    10.8190005 3.223235e+00   11.0418668   0.34884564    2.2795134
## coef.var    0.3643237 6.984844e-02    0.2128227   0.13833394    0.0874441
##             HeadWidth    radioulna        femur        tibia         foot
## nbr.val   85.00000000  85.00000000   85.0000000 8.500000e+01 8.500000e+01
## nbr.null   0.00000000   0.00000000    0.0000000 0.000000e+00 0.000000e+00
## nbr.na     0.00000000   0.00000000    0.0000000 0.000000e+00 0.000000e+00
## min        6.40000000   3.60000000    8.5000000 9.300000e+00 1.240000e+01
## max        9.50000000   7.00000000   13.6000000 1.490000e+01 2.090000e+01
## range      3.10000000   3.40000000    5.1000000 5.600000e+00 8.500000e+00
## sum      682.20000000 467.60000000  913.8000000 1.055050e+03 1.487500e+03
## median     8.00000000   5.50000000   10.5000000 1.230000e+01 1.760000e+01
## mean       8.02588235   5.50117647   10.7505882 1.241235e+01 1.750000e+01
## SE.mean    0.06880641   0.05823607    0.1306697 1.278510e-01 1.714312e-01
## CI.mean    0.13682909   0.11580882    0.2598511 2.542457e-01 3.409097e-01
## var        0.40241737   0.28827241    1.4513389 1.389399e+00 2.498036e+00
## std.dev    0.63436375   0.53691006    1.2047153 1.178728e+00 1.580518e+00
## coef.var   0.07903975   0.09759913    0.1120604 9.496409e-02 9.031529e-02
```

```
##                    SMI           PC1           DOY          DOY2      latitude
## nbr.val    85.00000000  8.500000e+01 8.500000e+01 8.500000e+01 8.500000e+01
## nbr.null    0.00000000  0.000000e+00 0.000000e+00 0.000000e+00 0.000000e+00
## nbr.na      0.00000000  0.000000e+00 0.000000e+00 0.000000e+00 0.000000e+00
## min         2.08620435 -3.304874e+00 1.180000e+02 1.392400e+04 4.267162e+01
## max         3.13456216  1.773412e+00 1.370000e+02 1.876900e+04 4.354562e+01
## range       1.04835780  5.078286e+00 1.900000e+01 4.845000e+03 8.740070e-01
## sum       214.79295758 -4.697127e-01 1.083900e+04 1.385029e+06 3.646767e+03
## median      2.53130649  3.753201e-02 1.290000e+02 1.664100e+04 4.272510e+01
## mean        2.52697597 -5.526032e-03 1.275176e+02 1.629446e+04 4.290314e+01
## SE.mean     0.02560745  1.089656e-01 6.334760e-01 1.613562e+02 3.082133e-02
## CI.mean     0.05092323  2.166900e-01 1.259736e+00 3.208745e+02 6.129159e-02
## var         0.05573804  1.009247e+00 3.410980e+01 2.213046e+06 8.074611e-02
## std.dev     0.23608904  1.004613e+00 5.840360e+00 1.487631e+03 2.841586e-01
## coef.var    0.09342750 -1.817965e+02 4.580041e-02 9.129673e-02 6.623258e-03
##             TotalSperm     Abnormal         noAb     CytoDrop         noCD
## nbr.val     85.000000   85.0000000   85.0000000   85.0000000   85.000000
## nbr.null     0.000000    0.0000000    1.0000000    0.0000000    0.000000
## nbr.na       0.000000    0.0000000    0.0000000    0.0000000    0.000000
## min         27.000000   11.0000000    0.0000000    6.0000000    3.000000
## max        187.000000   97.0000000  100.0000000   91.0000000  116.000000
## range      160.000000   86.0000000  100.0000000   85.0000000  113.000000
## sum       6804.000000 3822.0000000 2982.0000000 3025.0000000 3779.000000
## median      79.000000   44.0000000   32.0000000   33.0000000   42.000000
## mean        80.047059   44.9647059   35.0823529   35.5882353   44.458824
## SE.mean      2.561780    1.8108332    1.9561876    1.8088867    2.157623
## CI.mean      5.094381    3.6010404    3.8900936    3.5971696    4.290670
## var        557.831092  278.7249300  325.2669468  278.1260504  395.703641
## std.dev     23.618448   16.6950571   18.0351586   16.6771116   19.892301
## coef.var     0.295057    0.3712925    0.5140806    0.4686131    0.447432
##                 NoTail         no0t      NoHead          no0H   TwoTails
## nbr.val    85.0000000   85.0000000 85.0000000   85.0000000 85.0000000
## nbr.null    5.0000000    0.0000000 42.0000000    0.0000000 49.0000000
## nbr.na      0.0000000    0.0000000  0.0000000    0.0000000  0.0000000
## min         0.0000000   24.0000000  0.0000000   27.0000000  0.0000000
## max        45.0000000  171.0000000  5.0000000  185.0000000  9.0000000
## range      45.0000000  147.0000000  5.0000000  158.0000000  9.0000000
## sum       746.0000000 6058.0000000 79.0000000 6725.0000000 79.0000000
## median      6.0000000   71.0000000  1.0000000   78.0000000  0.0000000
## mean        8.7764706   71.2705882  0.9294118   79.1176471  0.9294118
## SE.mean     0.8138122    2.5456828  0.1304895    2.5521130  0.1928616
## CI.mean     1.6183548    5.0623694  0.2594927    5.0751565  0.3835264
## var        56.2946779  550.8425770  1.4473389  553.6288515  3.1616246
```

```
## std.dev    7.5029779   23.4700357  1.2030540   23.5293190  1.7780958
## coef.var   0.8548969    0.3293089  1.2944252    0.2973966  1.9131410
##                 no2t    TwoHeads         no2H       Other        noOTH
## nbr.val    85.0000000 85.00000000   85.0000000 85.00000000   85.0000000
## nbr.null    0.0000000 83.00000000    0.0000000 79.00000000    0.0000000
## nbr.na      0.0000000  0.00000000    0.0000000  0.00000000    0.0000000
## min        27.0000000  0.00000000   27.0000000  0.00000000   27.0000000
## max       187.0000000  1.00000000  187.0000000  2.00000000  187.0000000
## range     160.0000000  1.00000000  160.0000000  2.00000000  160.0000000
## sum      6725.0000000  2.00000000 6802.0000000  7.00000000 6797.0000000
## median     78.0000000  0.00000000   79.0000000  0.00000000   79.0000000
## mean       79.1176471  0.02352941   80.0235294  0.08235294   79.9647059
## SE.mean     2.5768530  0.01653848    2.5619483  0.03434772    2.5566658
## CI.mean     5.1243548  0.03288857    5.0947151  0.06830421    5.0842102
## var       564.4145658  0.02324930  557.9042017  0.10028011  555.6058824
## std.dev    23.7574108  0.15247721   23.6199958  0.31667035   23.5712936
## coef.var    0.3002795  6.48028145    0.2951631  3.84528285    0.2947712
##             Pabnormal         Pcd      P0tails      P0heads      P2tails
## nbr.val   85.00000000 85.00000000 85.000000000 85.000000000 8.500000e+01
## nbr.null   0.00000000  0.00000000  5.000000000 42.000000000 4.900000e+01
## nbr.na     0.00000000  0.00000000  0.000000000  0.000000000 0.000000e+00
## min        0.26785714  0.07500000  0.000000000  0.000000000 0.000000e+00
## max        1.00000000  0.96511628  0.569620250  0.074626870 1.428571e-01
## range      0.73214286  0.89011628  0.569620250  0.074626870 1.428571e-01
## sum       48.16085638 37.87726710  9.627206470  1.032945160 1.085882e+00
## median     0.55000000  0.42857143  0.085561500  0.008695650 0.000000e+00
## mean       0.56659831  0.44561491  0.113261253  0.012152296 1.277508e-02
## SE.mean    0.01708451  0.01812698  0.010584756  0.001817056 2.737904e-03
## CI.mean    0.03397441  0.03604748  0.021048949  0.003613416 5.444622e-03
## var        0.02480983  0.02792992  0.009523151  0.000280644 6.371701e-04
## std.dev    0.15751135  0.16712248  0.097586631  0.016752432 2.524223e-02
## coef.var   0.27799475  0.37503791  0.861606500  1.378540466 1.975895e+00
##                P2heads       Pother           PC2
## nbr.val   8.500000e+01 8.500000e+01  8.500000e+01
## nbr.null  8.300000e+01 7.900000e+01  0.000000e+00
## nbr.na    0.000000e+00 0.000000e+00  0.000000e+00
## min       0.000000e+00 0.000000e+00 -3.304874e+00
## max       1.298701e-02 1.869159e-02  1.773412e+00
## range     1.298701e-02 1.869159e-02  5.078286e+00
## sum       2.533269e-02 7.734177e-02 -4.697127e-01
## median    0.000000e+00 0.000000e+00  3.753201e-02
## mean      2.980316e-04 9.099032e-04 -5.526032e-03
## SE.mean   2.095508e-04 3.738564e-04  1.089656e-01
```

```
## CI.mean  4.167147e-04 7.434544e-04  2.166900e-01
## var      3.732480e-06 1.188033e-05  1.009247e+00
## std.dev  1.931963e-03 3.446786e-03  1.004613e+00
## coef.var 6.482408e+00 3.788080e+00 -1.817965e+02
```

```
#test effect of body condition on proportion of sperm that had any abnormality
mod11 <- glm(cbind(Abnormal,noAb)~SMI,family=binomial, data=dat3)
summary(mod11) #serious overdispersion indicated
```

```
##
## Call:
## glm(formula = cbind(Abnormal, noAb) ~ SMI, family = binomial,
##     data = dat3)
##
## Deviance Residuals:
##     Min       1Q   Median       3Q      Max
## -5.7155  -1.9801  -0.1894   1.4382   8.5646
##
## Coefficients:
##             Estimate Std. Error z value Pr(>|z|)
## (Intercept)  -0.3037     0.2553  -1.190   0.2342
## SMI           0.2187     0.1007   2.171   0.0299 *
## ---
## Signif. codes:  0 '***' 0.001 '**' 0.01 '*' 0.05 '.' 0.1 ' ' 1
##
## (Dispersion parameter for binomial family taken to be 1)
##
##     Null deviance: 725.89  on 84  degrees of freedom
## Residual deviance: 721.17  on 83  degrees of freedom
## AIC: 1117.5
##
## Number of Fisher Scoring iterations: 4
```

```
mod11a <- glm.binomial.disp(mod11) #correct mod11 for overdispersion
```

```
##
## Binomial overdispersed logit model fitting...
## Iter.  1  phi: 0.08849251
## Iter.  2  phi: 0.08825316
## Iter.  3  phi: 0.08825263
## Iter.  4  phi: 0.08825263
## Converged after 4 iterations.
## Estimated dispersion parameter: 0.08825263
##
## Call:
```

```
## glm(formula = cbind(Abnormal, noAb) ~ SMI, family = binomial,
##     data = dat3, weights = disp.weights)
##
## Deviance Residuals:
##     Min       1Q   Median       3Q      Max
## -1.8838  -0.7326  -0.1041   0.5417   2.9154
##
## Coefficients:
##             Estimate Std. Error z value Pr(>|z|)
## (Intercept)  -0.6033     0.7508  -0.804    0.422
## SMI           0.3448     0.2963   1.164    0.245
##
## (Dispersion parameter for binomial family taken to be 1)
##
##     Null deviance: 92.657  on 84  degrees of freedom
## Residual deviance: 91.299  on 83  degrees of freedom
## AIC: 146.68
##
## Number of Fisher Scoring iterations: 4
```

```
plot(mod11)
```

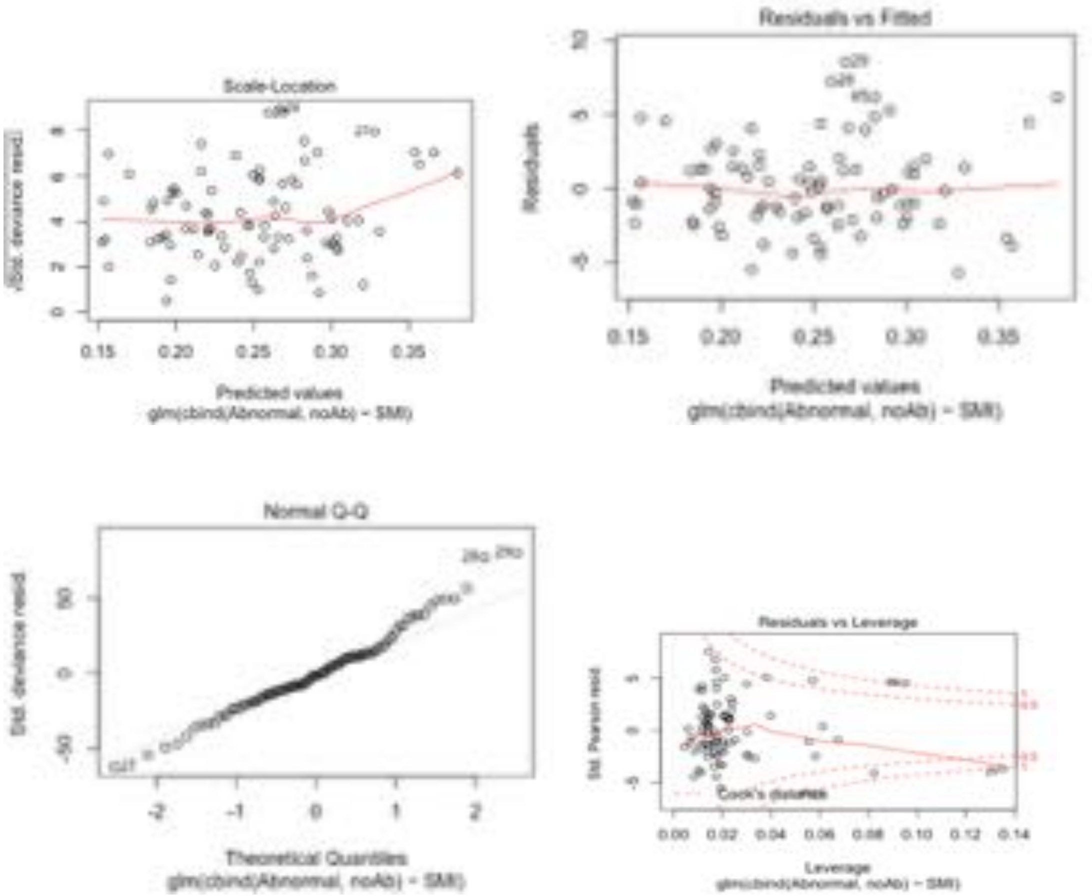


```
mod11b <- glm(cbind(Abnormal,noAb)~1,family=binomial, data=dat3) #remove SMI to test
for significance
mod11c <- glm.binomial.disp(mod11b) #correct mod11b for overdispersion

##
## Binomial overdispersed logit model fitting...
## Iter.  1  phi: 0.08751271
## Iter.  2  phi: 0.08813602
## Iter.  3  phi: 0.0881383
## Iter.  4  phi: 0.08813831
## Converged after 4 iterations.
## Estimated dispersion parameter: 0.08813831
##
## Call:
## glm(formula = cbind(Abnormal, noAb) ~ 1, family = binomial, data = dat3,
##     weights = disp.weights)
##
## Deviance Residuals:
##     Min       1Q   Median       3Q      Max
```

```
## -1.8717  -0.6843  -0.1026   0.5085   3.1655
##
## Coefficients:
##             Estimate Std. Error z value Pr(>|z|)
## (Intercept)  0.26705    0.06937    3.85 0.000118 ***
## ---
## Signif. codes:  0 '***' 0.001 '**' 0.01 '*' 0.05 '.' 0.1 ' ' 1
##
## (Dispersion parameter for binomial family taken to be 1)
##
##     Null deviance: 92.76  on 84  degrees of freedom
## Residual deviance: 92.76  on 84  degrees of freedom
## AIC: 146.2
##
## Number of Fisher Scoring iterations: 4
```

```
anova(mod11a, mod11c, test="Chisq") #compare models with and without SMI to test for significance of SMI
```

```
## Analysis of Deviance Table
##
## Model 1: cbind(Abnormal, noAb) ~ SMI
## Model 2: cbind(Abnormal, noAb) ~ 1
##   Resid. Df Resid. Dev Df Deviance Pr(>Chi)
## 1        83     91.299
## 2        84     92.760 -1  -1.4613   0.2267
```

```
##the models mod12-mo17) below follow these same steps to assess the effect of SMI on the proportions of abnormalities#test effect of body condition on proportion of sperm that had cytoplasmic drops
mod12 <- glm(cbind(CytoDrop,noCD)~SMI,family=binomial, data=dat3)
summary(mod12) #serious overdispersion indicated
```

```
##
## Call:
## glm(formula = cbind(CytoDrop, noCD) ~ SMI, family = binomial,
##     data = dat3)
##
## Deviance Residuals:
##    Min      1Q  Median      3Q     Max
## -7.216  -2.323  -0.411   1.266  10.442
##
## Coefficients:
##             Estimate Std. Error z value Pr(>|z|)
## (Intercept)  -1.2365     0.2553  -4.842 1.28e-06 ***
## SMI           0.4014     0.1006   3.991 6.58e-05 ***
```

```
## ---
## Signif. codes:  0 '***' 0.001 '**' 0.01 '*' 0.05 '.' 0.1 ' ' 1
##
## (Dispersion parameter for binomial family taken to be 1)
##
##     Null deviance: 814.73  on 84  degrees of freedom
## Residual deviance: 798.77  on 83  degrees of freedom
## AIC: 1197.4
##
## Number of Fisher Scoring iterations: 3
```

```
mod12a <- glm.binomial.disp(mod12) #correct for overdispersion
```

```
##
## Binomial overdispersed logit model fitting...
## Iter.  1  phi: 0.1022556
## Iter.  2  phi: 0.09977373
## Iter.  3  phi: 0.09978421
## Iter.  4  phi: 0.09978416
## Converged after 4 iterations.
## Estimated dispersion parameter: 0.09978416
##
## Call:
## glm(formula = cbind(CytoDrop, noCD) ~ SMI, family = binomial,
##     data = dat3, weights = disp.weights)
##
## Deviance Residuals:
##     Min       1Q   Median       3Q      Max
## -2.4197  -0.7595  -0.1325   0.4143   3.3777
##
## Coefficients:
##             Estimate Std. Error z value Pr(>|z|)
## (Intercept)  -1.3914     0.7911  -1.759   0.0786 .
## SMI           0.4645     0.3115   1.491   0.1360
## ---
## Signif. codes:  0 '***' 0.001 '**' 0.01 '*' 0.05 '.' 0.1 ' ' 1
##
## (Dispersion parameter for binomial family taken to be 1)
##
##     Null deviance: 90.405  on 84  degrees of freedom
## Residual deviance: 88.175  on 83  degrees of freedom
## AIC: 139.02
##
## Number of Fisher Scoring iterations: 3
```

```r
plot(mod12)
```

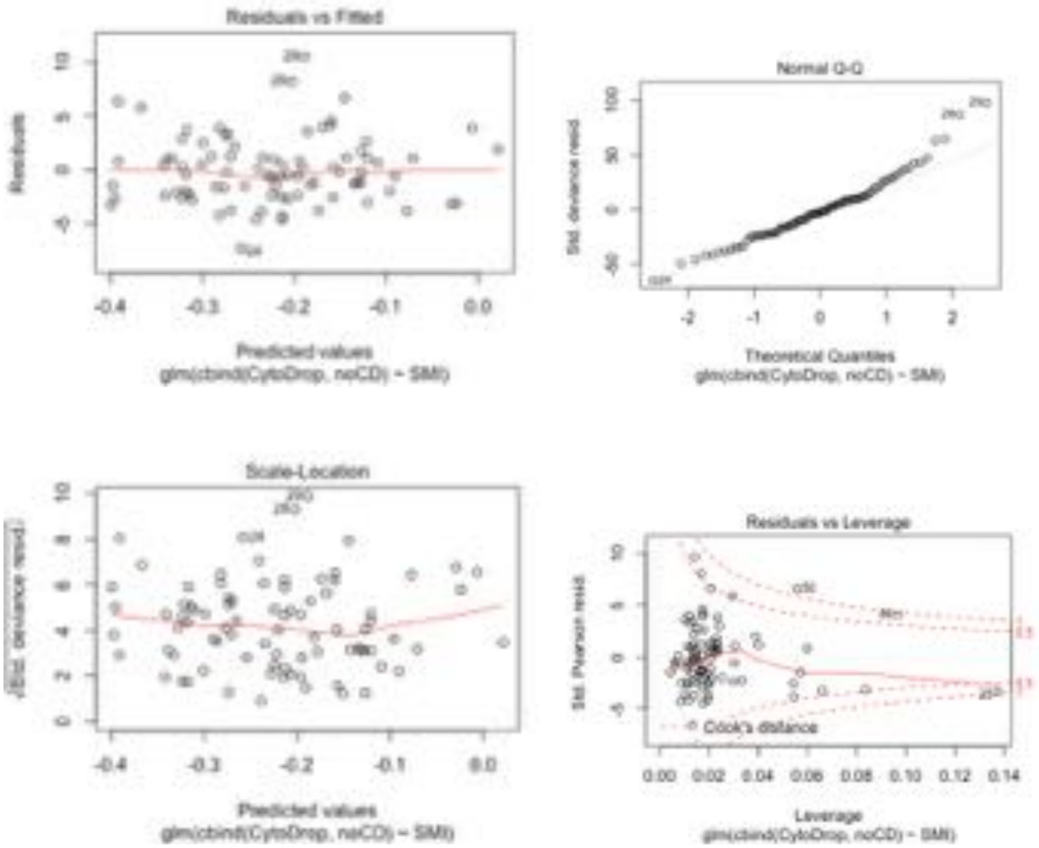


```r
shapiro.test(residuals(mod12))

##
##  Shapiro-Wilk normality test
##
## data:  residuals(mod12)
## W = 0.96573, p-value = 0.02327

mod12b <- glm(cbind(CytoDrop,noCD)~1,family=binomial, data=dat3)
mod12c <- glm.binomial.disp(mod12b) #correct for overdispersion

##
## Binomial overdispersed logit model fitting...
## Iter.  1  phi: 0.1027672
## Iter.  2  phi: 0.1011135
## Iter.  3  phi: 0.1011193
## Iter.  4  phi: 0.1011193
```

```
## Converged after 4 iterations.
## Estimated dispersion parameter: 0.1011193
##
## Call:
## glm(formula = cbind(CytoDrop, noCD) ~ 1, family = binomial, data = dat3,
##     weights = disp.weights)
##
## Deviance Residuals:
##     Min       1Q   Median       3Q      Max
## -2.4609  -0.7654  -0.1050   0.5418   3.4106
##
## Coefficients:
##             Estimate Std. Error z value Pr(>|z|)
## (Intercept) -0.21754    0.07345  -2.962  0.00306 **
## ---
## Signif. codes:  0 '***' 0.001 '**' 0.01 '*' 0.05 '.' 0.1 ' ' 1
##
## (Dispersion parameter for binomial family taken to be 1)
##
##     Null deviance: 89.35  on 84  degrees of freedom
## Residual deviance: 89.35  on 84  degrees of freedom
## AIC: 137.65
##
## Number of Fisher Scoring iterations: 3
```

```
anova(mod12a, mod12c, test="Chisq")
```

```
## Analysis of Deviance Table
##
## Model 1: cbind(CytoDrop, noCD) ~ SMI
## Model 2: cbind(CytoDrop, noCD) ~ 1
##   Resid. Df Resid. Dev Df Deviance Pr(>Chi)
## 1        83     88.175
## 2        84     89.350 -1  -1.1749   0.2784
```

```
#test effect of body condition on proportion of sperm that had no tail
mod13 <- glm(cbind(NoTail,no0t)~SMI,family=binomial, data=dat3)
summary(mod13) #serious overdispersion indicated
```

```
##
## Call:
## glm(formula = cbind(NoTail, no0t) ~ SMI, family = binomial, data = dat3)
##
## Deviance Residuals:
##     Min       1Q   Median       3Q      Max
```

```
## -4.5363  -2.0899  -0.5037   1.0700   9.5925
##
## Coefficients:
##             Estimate Std. Error z value Pr(>|z|)
## (Intercept)  -3.1423     0.4049   -7.76 8.49e-15 ***
## SMI           0.4135     0.1584    2.61  0.00906 **
## ---
## Signif. codes:  0 '***' 0.001 '**' 0.01 '*' 0.05 '.' 0.1 ' ' 1
##
## (Dispersion parameter for binomial family taken to be 1)
##
##     Null deviance: 532.71  on 84  degrees of freedom
## Residual deviance: 525.93  on 83  degrees of freedom
## AIC: 826.25
##
## Number of Fisher Scoring iterations: 5
```

```
mod13a <- glm.binomial.disp(mod13) #correct for overdispersion
```

```
##
## Binomial overdispersed logit model fitting...
## Iter.  1  phi: 0.07713806
## Iter.  2  phi: 0.07741014
## Iter.  3  phi: 0.07740949
## Iter.  4  phi: 0.07740949
## Converged after 4 iterations.
## Estimated dispersion parameter: 0.07740949
##
## Call:
## glm(formula = cbind(NoTail, no0t) ~ SMI, family = binomial, data = dat3,
##     weights = disp.weights)
##
## Deviance Residuals:
##     Min       1Q   Median       3Q      Max
## -1.7107  -0.7994  -0.2360   0.4160   3.5191
##
## Coefficients:
##             Estimate Std. Error z value Pr(>|z|)
## (Intercept)  -3.5345     1.1107  -3.182  0.00146 **
## SMI           0.5802     0.4336   1.338  0.18088
## ---
## Signif. codes:  0 '***' 0.001 '**' 0.01 '*' 0.05 '.' 0.1 ' ' 1
##
## (Dispersion parameter for binomial family taken to be 1)
```

```
##
##     Null deviance: 78.142  on 84  degrees of freedom
## Residual deviance: 76.359  on 83  degrees of freedom
## AIC: 123.89
##
## Number of Fisher Scoring iterations: 5
plot(mod13)
```

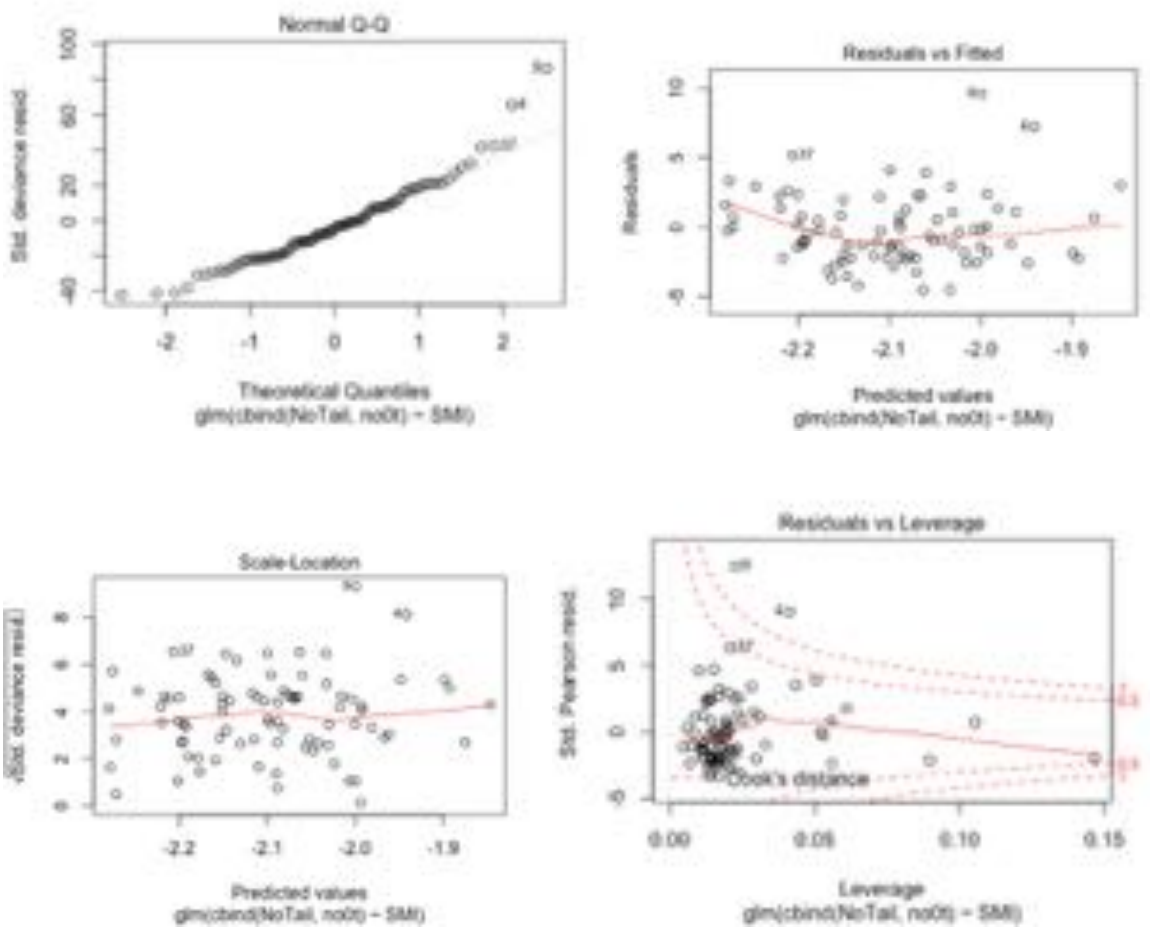


```
shapiro.test(residuals(mod13))

##
##  Shapiro-Wilk normality test
##
## data:  residuals(mod13)
## W = 0.93819, p-value = 0.0005062

mod13b <- glm(cbind(NoTail,no0t)~1,family=binomial, data=dat3)
mod13c <- glm.binomial.disp(mod13b) #correct for overdispersion
```

```
##
## Binomial overdispersed logit model fitting...
## Iter.  1  phi: 0.07975372
## Iter.  2  phi: 0.08211482
## Iter.  3  phi: 0.08211891
## Iter.  4  phi: 0.08211892
## Converged after 4 iterations.
## Estimated dispersion parameter: 0.08211892
##
## Call:
## glm(formula = cbind(NoTail, no0t) ~ 1, family = binomial, data = dat3,
##     weights = disp.weights)
##
## Deviance Residuals:
##     Min       1Q   Median       3Q      Max
## -1.6067  -0.6999  -0.3011   0.4175   3.6099
##
## Coefficients:
##             Estimate Std. Error z value Pr(>|z|)
## (Intercept)  -2.0617     0.1054  -19.57   <2e-16 ***
## ---
## Signif. codes:  0 '***' 0.001 '**' 0.01 '*' 0.05 '.' 0.1 ' ' 1
##
## (Dispersion parameter for binomial family taken to be 1)
##
##     Null deviance: 74.318  on 84  degrees of freedom
## Residual deviance: 74.318  on 84  degrees of freedom
## AIC: 117.72
##
## Number of Fisher Scoring iterations: 5
```

```
anova(mod13a, mod13c, test="Chisq")
```

```
## Analysis of Deviance Table
##
## Model 1: cbind(NoTail, no0t) ~ SMI
## Model 2: cbind(NoTail, no0t) ~ 1
##   Resid. Df Resid. Dev Df Deviance Pr(>Chi)
## 1        83     76.359
## 2        84     74.318 -1   2.0402
```

```
#test effect of body condition on proportion of sperm that had two tails
mod14 <- glm(cbind(TwoTails,no2t)~SMI,family=binomial, data=dat3)
summary(mod14) #some overdispersion indicated
```

```
##
## Call:
## glm(formula = cbind(TwoTails, no2t) ~ SMI, family = binomial,
##     data = dat3)
##
## Deviance Residuals:
##     Min       1Q   Median       3Q      Max
## -2.0232  -1.3492  -1.1694   0.0293   5.4273
##
## Coefficients:
##             Estimate Std. Error z value Pr(>|z|)
## (Intercept)  -4.1066     1.1825  -3.473 0.000515 ***
## SMI          -0.1339     0.4676  -0.286 0.774613
## ---
## Signif. codes:  0 '***' 0.001 '**' 0.01 '*' 0.05 '.' 0.1 ' ' 1
##
## (Dispersion parameter for binomial family taken to be 1)
##
##     Null deviance: 203.42  on 84  degrees of freedom
## Residual deviance: 203.34  on 83  degrees of freedom
## AIC: 294.2
##
## Number of Fisher Scoring iterations: 5
```

```
mod14a <- glm.binomial.disp(mod14) #correct for overdispersion
```

```
##
## Binomial overdispersed logit model fitting...
## Iter.  1  phi: 0.03676018
## Iter.  2  phi: 0.03777227
## Iter.  3  phi: 0.03777623
## Iter.  4  phi: 0.03777625
## Converged after 4 iterations.
## Estimated dispersion parameter: 0.03777625
##
## Call:
## glm(formula = cbind(TwoTails, no2t) ~ SMI, family = binomial,
##     data = dat3, weights = disp.weights)
##
## Deviance Residuals:
##      Min        1Q    Median        3Q       Max
## -0.75910  -0.70821  -0.68140  -0.01535   2.91955
##
## Coefficients:
```

```
##             Estimate Std. Error z value Pr(>|z|)
## (Intercept) -4.28514    2.36471  -1.812    0.070 .
## SMI         -0.03371    0.93328  -0.036    0.971
## ---
## Signif. codes:  0 '***' 0.001 '**' 0.01 '*' 0.05 '.' 0.1 ' ' 1
##
## (Dispersion parameter for binomial family taken to be 1)
##
##     Null deviance: 53.945  on 84  degrees of freedom
## Residual deviance: 53.944  on 83  degrees of freedom
## AIC: 80.589
##
## Number of Fisher Scoring iterations: 5
```

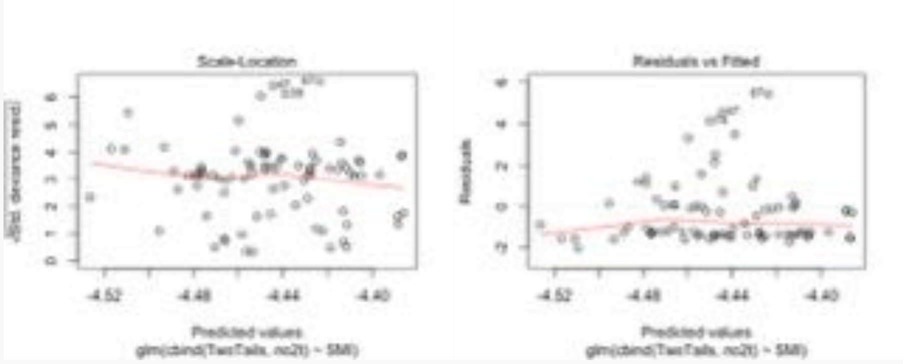


```
plot(mod14)
```

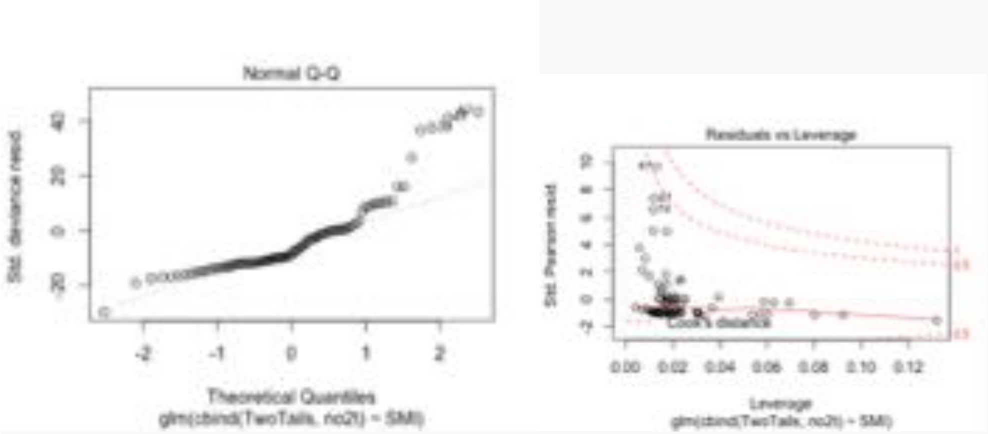


```
mod14b <- glm(cbind(TwoTails,no2t)~1,family=binomial, data=dat3)
mod14c <- glm.binomial.disp(mod14b) #correct for overdispersion
```

```
##
## Binomial overdispersed logit model fitting...
## Iter.  1  phi: 0.03622566
## Iter.  2  phi: 0.03720586
```

```
## Iter.  3  phi: 0.03721006
## Iter.  4  phi: 0.03721008
## Converged after 4 iterations.
## Estimated dispersion parameter: 0.03721008
##
## Call:
## glm(formula = cbind(TwoTails, no2t) ~ 1, family = binomial, data = dat3,
##     weights = disp.weights)
##
## Deviance Residuals:
##      Min        1Q    Median        3Q       Max
## -0.77026  -0.71327  -0.68694  -0.01799   2.93964
##
## Coefficients:
##             Estimate Std. Error z value Pr(>|z|)
## (Intercept)  -4.3705     0.2185     -20   <2e-16 ***
## ---
## Signif. codes:  0 '***' 0.001 '**' 0.01 '*' 0.05 '.' 0.1 ' ' 1
##
## (Dispersion parameter for binomial family taken to be 1)
##
##     Null deviance: 54.537  on 84  degrees of freedom
## Residual deviance: 54.537  on 84  degrees of freedom
## AIC: 79.432
##
## Number of Fisher Scoring iterations: 5
```

```
anova(mod14a, mod14c, test="Chisq")
```

```
## Analysis of Deviance Table
##
## Model 1: cbind(TwoTails, no2t) ~ SMI
## Model 2: cbind(TwoTails, no2t) ~ 1
##   Resid. Df Resid. Dev Df Deviance Pr(>Chi)
## 1        83     53.944
## 2        84     54.537 -1 -0.59266   0.4414
```

```
#test effect of body condition on proportion of sperm that had no head
mod15 <- glm(cbind(NoHead,no0H)~SMI,family=binomial, data=dat3)
summary(mod15) #no serious overdispersion indicated
```

```
##
## Call:
## glm(formula = cbind(NoHead, no0H) ~ SMI, family = binomial, data = dat3)
##
```

```
## Deviance Residuals:
##     Min       1Q   Median       3Q      Max
## -1.7356  -1.3056  -0.3053   0.3132   3.2313
##
## Coefficients:
##              Estimate Std. Error z value Pr(>|z|)
## (Intercept) -4.449854   1.181249  -3.767 0.000165 ***
## SMI          0.002263   0.465655   0.005 0.996122
## ---
## Signif. codes:  0 '***' 0.001 '**' 0.01 '*' 0.05 '.' 0.1 ' ' 1
##
## (Dispersion parameter for binomial family taken to be 1)
##
##     Null deviance: 133.93  on 84  degrees of freedom
## Residual deviance: 133.93  on 83  degrees of freedom
## AIC: 240.76
##
## Number of Fisher Scoring iterations: 5
```

```
plot(mod15)
```

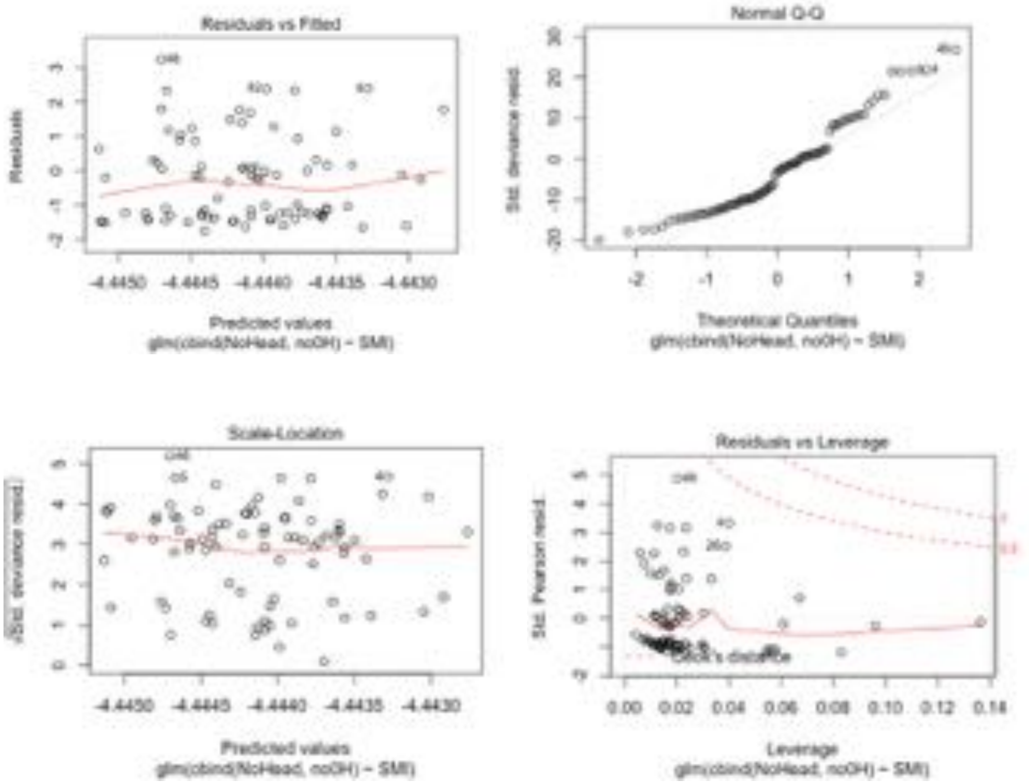

```
mod15a <- glm(cbind(NoHead,no0H)~1,family=binomial, data=dat3)

anova(mod15, mod15a, test="Chisq")

## Analysis of Deviance Table
##
## Model 1: cbind(NoHead, no0H) ~ SMI
## Model 2: cbind(NoHead, no0H) ~ 1
##   Resid. Df Resid. Dev Df    Deviance Pr(>Chi)
## 1        83     133.93
## 2        84     133.93 -1 -2.3621e-05   0.9961

#test effect of body condition on proportion of sperm that had two heads
mod16 <- glm(cbind(TwoHeads,no2H)~SMI,family=binomial, data=dat3)
summary(mod16) #some underdispersion indicated

##
## Call:
## glm(formula = cbind(TwoHeads, no2H) ~ SMI, family = binomial,
##     data = dat3)
##
## Deviance Residuals:
##     Min       1Q   Median       3Q      Max
## -0.3564  -0.2300  -0.2137  -0.1915   2.3699
##
## Coefficients:
##             Estimate Std. Error z value Pr(>|z|)
## (Intercept)  -8.9016     7.3714  -1.208    0.227
## SMI           0.3038     2.8853   0.105    0.916
##
## (Dispersion parameter for binomial family taken to be 1)
##
##     Null deviance: 15.077  on 84  degrees of freedom
## Residual deviance: 15.066  on 83  degrees of freedom
## AIC: 23.04
##
## Number of Fisher Scoring iterations: 7

plot(mod16)
```

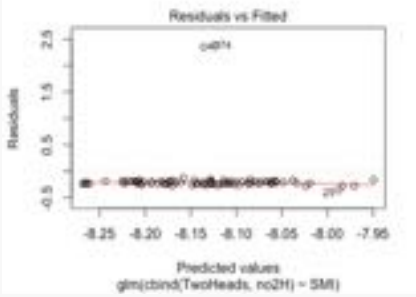

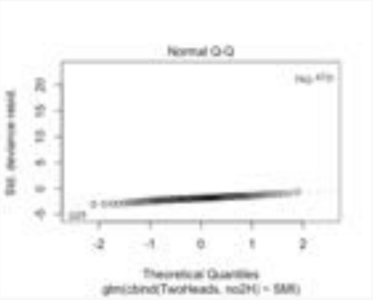

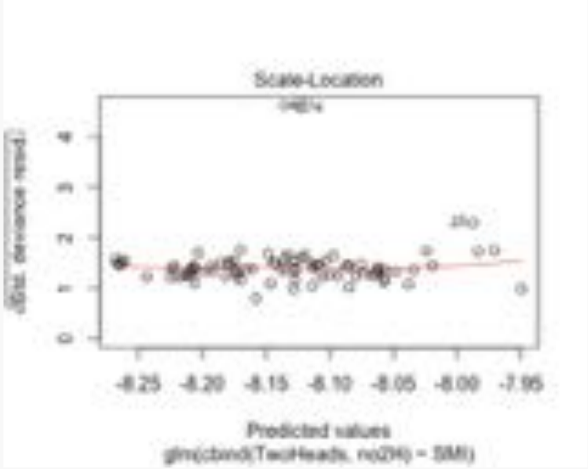

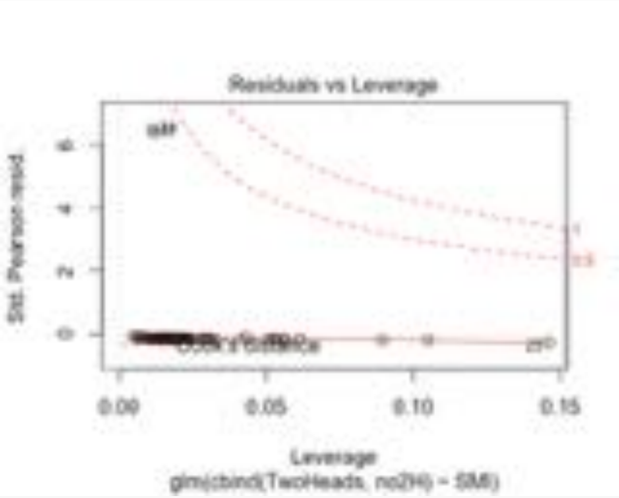


```
mod16a <- glm(cbind(TwoHeads,no2H)~1,family=binomial, data=dat3)

anova(mod16, mod16a, test="Chisq")

## Analysis of Deviance Table
##
## Model 1: cbind(TwoHeads, no2H) ~ SMI
## Model 2: cbind(TwoHeads, no2H) ~ 1
##   Resid. Df Resid. Dev Df  Deviance Pr(>Chi)
## 1        83     15.066
## 2        84     15.077 -1 -0.011024   0.9164

#test effect of body condition on proportion of sperm that had any 'other'
abnormalities
mod17 <- glm(cbind(Other,noOTH)~SMI,family=binomial, data=dat3)
summary(mod17) #some underdispersion indicated

##
## Call:
## glm(formula = cbind(Other, noOTH) ~ SMI, family = binomial, data = dat3)
##
## Deviance Residuals:
##     Min       1Q   Median       3Q      Max
## -0.6291  -0.4434  -0.3802  -0.2996   2.3374
```

```
##
## Coefficients:
##             Estimate Std. Error z value Pr(>|z|)
## (Intercept)   -2.785      4.067  -0.685    0.493
## SMI           -1.652      1.664  -0.993    0.321
##
## (Dispersion parameter for binomial family taken to be 1)
##
##     Null deviance: 35.815  on 84  degrees of freedom
## Residual deviance: 34.778  on 83  degrees of freedom
## AIC: 51.314
##
## Number of Fisher Scoring iterations: 6
```

```
plot(mod17)
```

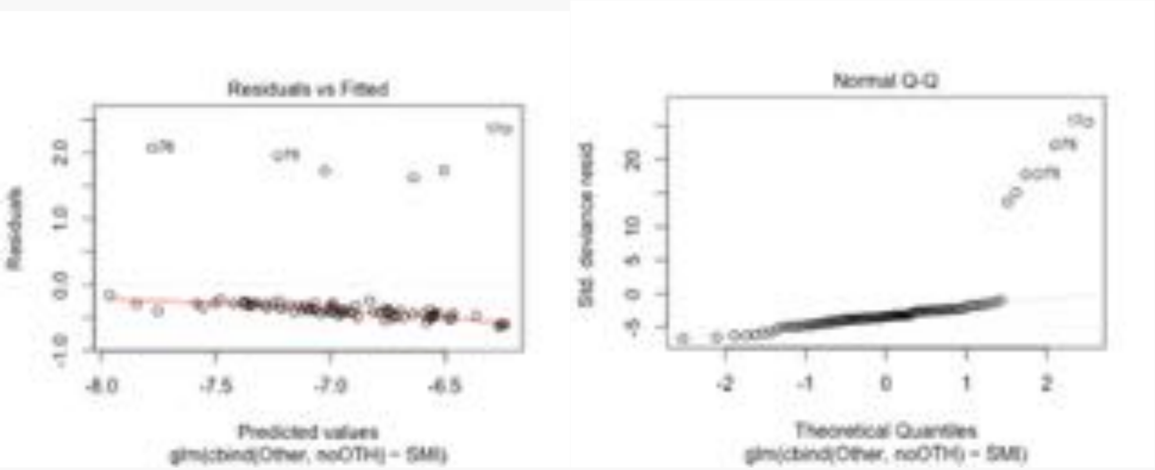

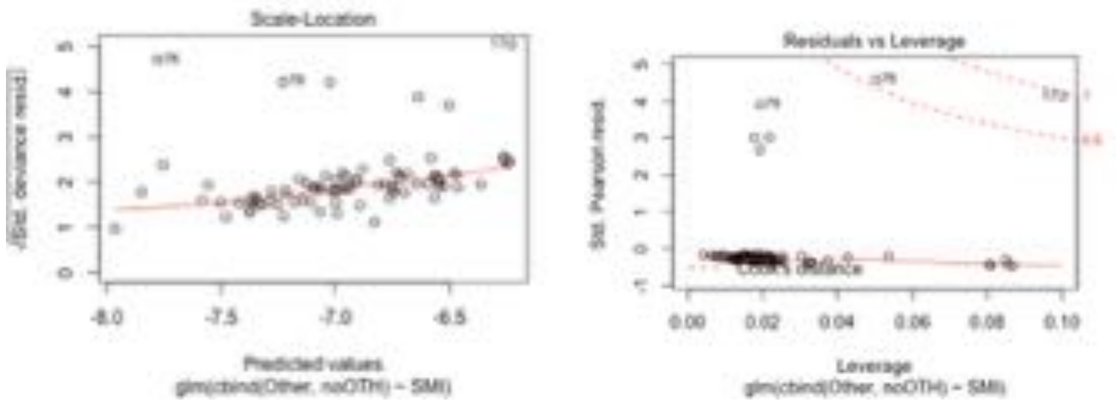


```
mod17a <- glm(cbind(Other,noOTH)~1,family=binomial, data=dat3)

anova(mod17, mod17a, test="Chisq")

## Analysis of Deviance Table
##
```

```
## Model 1: cbind(Other, noOTH) ~ SMI
## Model 2: cbind(Other, noOTH) ~ 1
##   Resid. Df Resid. Dev Df Deviance Pr(>Chi)
## 1        83     34.778
## 2        84     35.815 -1  -1.0363   0.3087
```

4.4 Comparing populations

4.4.1 Sperm size compared across populations

Analyses for Fig 4 (left) with some results reported in the text

```
#compare populations wrt sperm length accounting for seasonal variation, controlling
for condition
mod21<- lm(TOTspermlen~Population+DOY+DOY2+SMI+PC1, data=dat1)
m21 <- dredge(mod21)

## Fixed term is "(Intercept)"

subset (m21,delta<2.5)

## Global model call: lm(formula = TOTspermlen ~ Population + DOY + DOY2 + SMI + PC1,
##     data = dat1)
## ---
## Model selection table
##    (Intrc)   DOY    DOY2    PC1 Ppltn    SMI df   logLik  AICc delta
## 28   -7146 114.2 -0.4514            + -9.475 11 -308.364 642.3  0.00
## 12   -6531 104.1 -0.4112            +         10 -310.307 643.5  1.25
## 32   -7143 114.1 -0.4506 0.7975     + -8.864 12 -308.161 644.6  2.30
##    weight
## 28  0.540
## 12  0.289
## 32  0.171
## Models ranked by AICc(x)

mod21a <- lm(TOTspermlen~Population+DOY+DOY2+SMI, data=dat1) #best-fitting model
summary(mod21a)

##
## Call:
## lm(formula = TOTspermlen ~ Population + DOY + DOY2 + SMI, data = dat1)
##
## Residuals:
##      Min       1Q   Median       3Q      Max
## -20.9529  -5.5974  -0.1397   5.9250  19.5477
##
## Coefficients:
##                           Estimate Std. Error t value Pr(>|t|)
```

```
## (Intercept)           -7146.4128  2117.2824  -3.375 0.001164 **
## PopulationDereham         7.5861     3.6746   2.064 0.042385 *
## PopulationFingal          8.9858     3.4567   2.600 0.011210 *
## PopulationLaFortune      24.8872    10.1957   2.441 0.016975 *
## PopulationLong Point     30.4865    10.4003   2.931 0.004457 **
## PopulationPond Mills     17.3605     8.3270   2.085 0.040442 *
## PopulationStarky Hill    -1.1873     3.8393  -0.309 0.757984
## DOY                     114.2341    33.3275   3.428 0.000986 ***
## DOY2                     -0.4514     0.1309  -3.448 0.000924 ***
## SMI                      -9.4750     5.0549  -1.874 0.064714 .
## ---
## Signif. codes:  0 '***' 0.001 '**' 0.01 '*' 0.05 '.' 0.1 ' ' 1
##
## Residual standard error: 9.286 on 76 degrees of freedom
## Multiple R-squared:  0.3604, Adjusted R-squared:  0.2846
## F-statistic: 4.758 on 9 and 76 DF,  p-value: 4.963e-05
```

```
(m21a <- Anova(mod21a))
```

```
## Anova Table (Type II tests)
##
## Response: TOTspermlen
##            Sum Sq Df F value   Pr(>F)
## Population 1571.1  6  3.0367 0.010244 *
## DOY        1013.0  1 11.7486 0.000986 ***
## DOY2       1025.1  1 11.8885 0.000924 ***
## SMI         303.0  1  3.5135 0.064714 .
## Residuals  6553.2 76
## ---
## Signif. codes:  0 '***' 0.001 '**' 0.01 '*' 0.05 '.' 0.1 ' ' 1
```

```
m21a[5,1]/sum(m21a[,1]) #unexplained variation from this model
```

```
## [1] 0.6261803
```

```
plot(mod21a) #no departure from normality
```

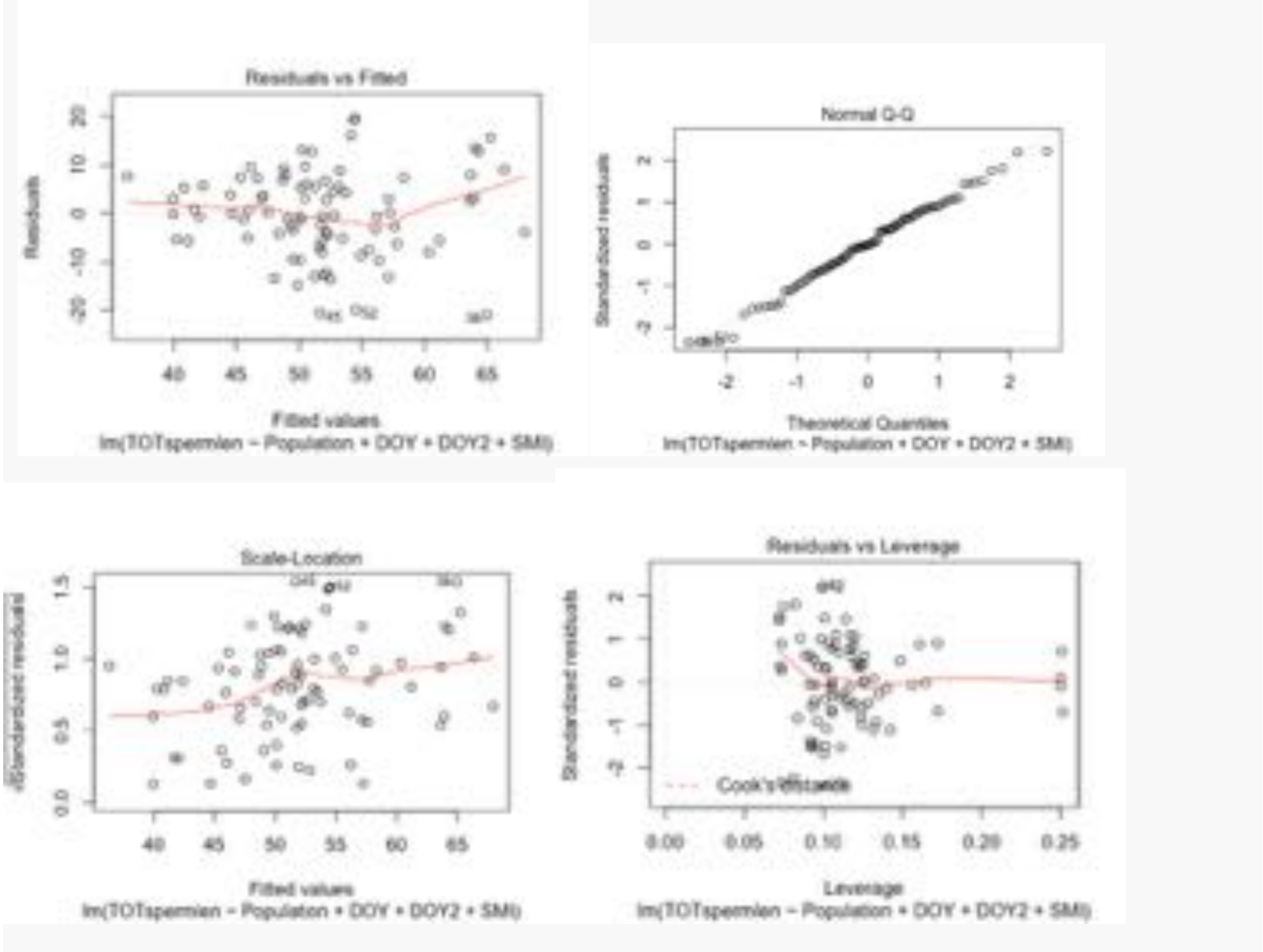


```
shapiro.test(residuals(mod21a)) #no sig departure from normality

##
##  Shapiro-Wilk normality test
##
## data:  residuals(mod21a)
## W = 0.98994, p-value = 0.7529

(cv21a <-rmse(mod21a$residuals)/mean(dat1$TOTspermlen) *100) #coeff of variation,
controlling for date and body condition

## [1] 16.83236

#test for interactions
mod21b<- lm(TOTspermlen~Population*DOY+SMI, data=dat1)
Anova(mod21b) #no sig interaction with DOY

## Anova Table (Type II tests)
##
## Response: TOTspermlen
```

```
##                 Sum Sq Df F value    Pr(>F)    
## Population     2233.3  6  4.4906 0.0006248 ***
## DOY             317.8  1  3.8335 0.0540074 .  
## SMI             257.2  1  3.1030 0.0822784 .  
## Population:DOY 1444.6  3  5.8093 0.0012741 ** 
## Residuals      6133.8 74                      
## ---
## Signif. codes:  0 '***' 0.001 '**' 0.01 '*' 0.05 '.' 0.1 ' ' 1

#regressions by population
summary((mod21cs <- lm(TOTspermlen~DOY, subset=(Population =="Calton Swamp"), data =
dat1)))

## 
## Call:
## lm(formula = TOTspermlen ~ DOY, data = dat1, subset = (Population == 
##     "Calton Swamp"))
## 
## Residuals:
##     Min      1Q  Median      3Q     Max 
## -12.689  -5.932  -3.469   4.975  22.419 
## 
## Coefficients:
##             Estimate Std. Error t value Pr(>|t|)
## (Intercept)  33.9874    77.7934   0.437    0.668
## DOY           0.1355     0.6161   0.220    0.829
## 
## Residual standard error: 10.73 on 17 degrees of freedom
## Multiple R-squared:  0.002836,   Adjusted R-squared:  -0.05582 
## F-statistic: 0.04835 on 1 and 17 DF,  p-value: 0.8286

summary((mod21de <- lm(TOTspermlen~DOY, subset=(Population =="Dereham"), data =
dat1)))

## 
## Call:
## lm(formula = TOTspermlen ~ DOY, data = dat1, subset = (Population == 
##     "Dereham"))
## 
## Residuals:
##     Min      1Q  Median      3Q     Max 
## -8.6222 -2.9639 -0.2824  2.8463 10.9416 
## 
## Coefficients:
##             Estimate Std. Error t value Pr(>|t|)   
## (Intercept) 130.0746    36.2687   3.586  0.00332 **
```

```
## DOY          -0.6119     0.2839  -2.155  0.05046 .
## ---
## Signif. codes:  0 '***' 0.001 '**' 0.01 '*' 0.05 '.' 0.1 ' ' 1
##
## Residual standard error: 5.485 on 13 degrees of freedom
## Multiple R-squared:  0.2633, Adjusted R-squared:  0.2066
## F-statistic: 4.646 on 1 and 13 DF,  p-value: 0.05046
```

```
summary((mod21fi <- lm(TOTspermlen~DOY, subset=(Population =="Fingal"), data = dat1)))
```

```
##
## Call:
## lm(formula = TOTspermlen ~ DOY, data = dat1, subset = (Population ==
##     "Fingal"))
##
## Residuals:
##     Min      1Q  Median      3Q     Max
## -23.922  -5.633   1.412   9.034  20.020
##
## Coefficients:
##             Estimate Std. Error t value Pr(>|t|)
## (Intercept)  832.052    252.326   3.298  0.00454 **
## DOY           -5.923      1.936  -3.060  0.00748 **
## ---
## Signif. codes:  0 '***' 0.001 '**' 0.01 '*' 0.05 '.' 0.1 ' ' 1
##
## Residual standard error: 12.24 on 16 degrees of freedom
## Multiple R-squared:  0.3691, Adjusted R-squared:  0.3297
## F-statistic: 9.362 on 1 and 16 DF,  p-value: 0.007484
```

```
summary((mod21lp <- lm(TOTspermlen~DOY, subset=(Population =="Long Point"), data =
dat1)))
```

```
##
## Call:
## lm(formula = TOTspermlen ~ DOY, data = dat1, subset = (Population ==
##     "Long Point"))
##
## Residuals:
##     Min      1Q  Median      3Q     Max
## -6.7439 -1.7439  0.3057  2.3713  6.2277
##
## Coefficients:
##             Estimate Std. Error t value Pr(>|t|)
## (Intercept)  718.702    428.802   1.676    0.138
```

```
## DOY           -4.943      3.140  -1.574    0.159
##
## Residual standard error: 4.681 on 7 degrees of freedom
## Multiple R-squared:  0.2614, Adjusted R-squared:  0.1559
## F-statistic: 2.477 on 1 and 7 DF,  p-value: 0.1595
```

```
boxplot(TOTspermlen~Population, data=dat1)
```

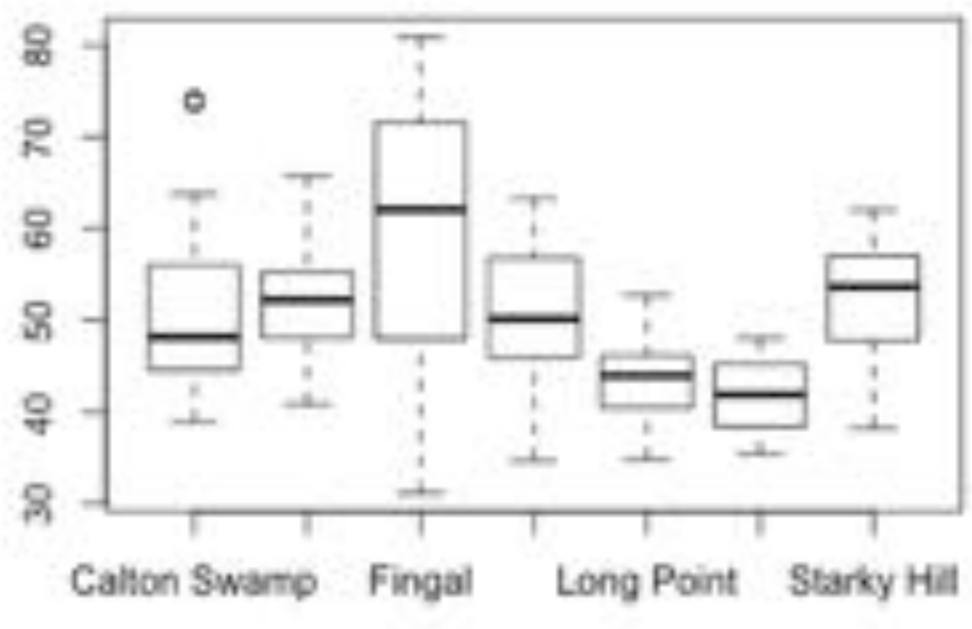


```
(HSD.test(mod21a, "Population")) #requires agricolae package
```

```
## $statistics
##       Mean       CV  MSerror     HSD r.harmonic
##   51.86016 17.90554 86.22685 12.8167    9.63028
##
## $parameters
##   Df ntr StudentizedRange alpha  test     name.t
##   76   7         4.283258  0.05 Tukey Population
##
## $means
##               TOTspermlen       std  r     Min      Max
## Calton Swamp    51.08408 10.439246 19 38.9083 74.01650
## Dereham         51.96161  6.157805 15 40.8106 65.75850
## Fingal          60.04645 14.954653 18 31.1295 80.88640
```

```
## LaFortune       49.85751  9.495520 10 34.6573 63.30900
## Long Point      43.77220  5.094951  9 34.8316 52.74580
## Pond Mills      41.80456  5.176343  4 35.4170 48.04724
## Starky Hill     51.76115  7.746727 11 38.1922 62.03620
##
## $comparison
## NULL
##
## $groups
##             trt    means  M
## 1 Fingal       60.04645  a
## 2 Dereham      51.96161 ab
## 3 Starky Hill  51.76115 ab
## 4 Calton Swamp 51.08408 ab
## 5 LaFortune    49.85751 ab
## 6 Long Point   43.77220  b
## 7 Pond Mills   41.80456  b
```

```
mod22<- lm(SpHeadLen~Population+DOY+DOY2+SMI+PC1, data=dat1)
m22 <- dredge(mod22)
```

```
## Fixed term is "(Intercept)"
```

```
subset (m22,delta<2.5)
```

```
## Global model call: lm(formula = SpHeadLen ~ Population + DOY + DOY2 + SMI + PC1,
##     data = dat1)
## ---
## Model selection table
##    (Intrc)     DOY       DOY2    PC1 Ppltn     SMI df   logLik  AICc delta
## 11   33.00          -0.0006928            +          9 -153.165 326.7  0.00
## 10   44.15 -0.1759                        +          9 -153.196 326.8  0.06
## 27   36.18          -0.0007339            + -1.0340 10 -152.257 327.4  0.75
## 26   47.97 -0.1862                        + -1.0290 10 -152.298 327.5  0.83
## 15   32.01          -0.0006313 0.2381     +         10 -152.467 327.9  1.17
## 14   42.16 -0.1602             0.2384     +         10 -152.497 327.9  1.23
## 12 -248.80  4.4310 -0.0180900             +         10 -152.784 328.5  1.80
## 28 -325.10  5.6870 -0.0230700             + -1.1740 11 -151.631 328.8  2.13
## 31   34.94          -0.0006786 0.1915     + -0.8884 11 -151.815 329.2  2.50
##    weight
## 11  0.183
## 10  0.178
## 27  0.126
## 26  0.121
## 15  0.102
```

```
## 14  0.099
## 12  0.074
## 28  0.063
## 31  0.053
## Models ranked by AICc(x)

mod22a<- lm(SpHeadLen~Population+DOY2, data=dat1)
summary(mod22a)

##
## Call:
## lm(formula = SpHeadLen ~ Population + DOY2, data = dat1)
##
## Residuals:
##     Min      1Q  Median      3Q     Max
## -3.3870 -0.9706 -0.0969  1.0378  5.3836
##
## Coefficients:
##                           Estimate Std. Error t value Pr(>|t|)
## (Intercept)             32.9958591  3.5392909   9.323 2.56e-14 ***
## PopulationDereham        1.0345662  0.5275750   1.961 0.053450 .
## PopulationFingal         0.7637608  0.5470380   1.396 0.166623
## PopulationLaFortune     -0.5616957  0.7392445  -0.760 0.449650
## PopulationLong Point     1.1029584  0.8537335   1.292 0.200198
## PopulationPond Mills     3.7462274  0.9705620   3.860 0.000232 ***
## PopulationStarky Hill -0.4863223  0.5850477  -0.831 0.408369
## DOY2                    -0.0006928  0.0002209  -3.136 0.002413 **
## ---
## Signif. codes:  0 '***' 0.001 '**' 0.01 '*' 0.05 '.' 0.1 ' ' 1
##
## Residual standard error: 1.508 on 78 degrees of freedom
## Multiple R-squared:  0.2439, Adjusted R-squared:  0.1761
## F-statistic: 3.595 on 7 and 78 DF,  p-value: 0.002092

Anova(mod22a)

## Anova Table (Type II tests)
##
## Response: SpHeadLen
##             Sum Sq Df F value   Pr(>F)
## Population  43.765  6   3.207 0.007233 **
## DOY2        22.374  1   9.837 0.002413 **
## Residuals  177.409 78
## ---
## Signif. codes:  0 '***' 0.001 '**' 0.01 '*' 0.05 '.' 0.1 ' ' 1
```

```
plot(mod22a) #no departure from normality
```

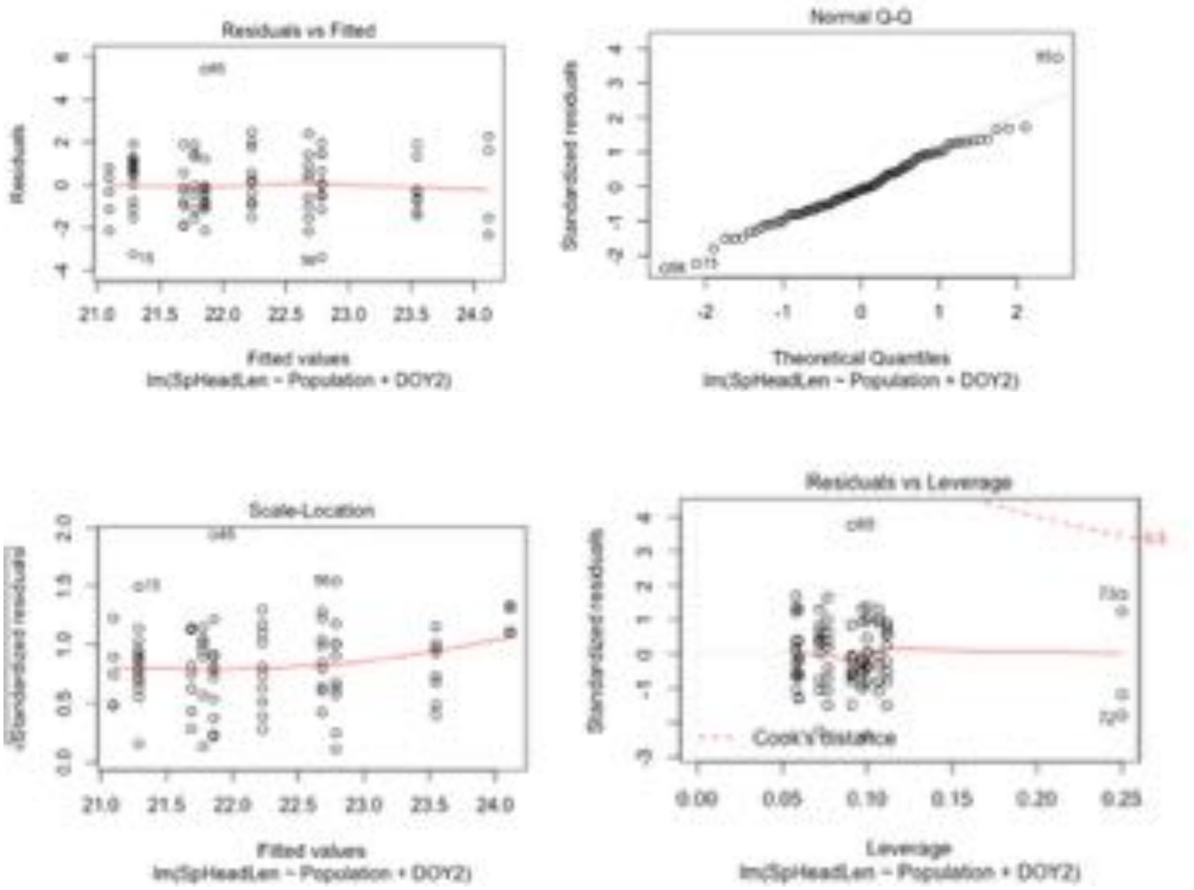


```
shapiro.test(residuals(mod22a)) #no sig departure from normality

##
##  Shapiro-Wilk normality test
##
## data:  residuals(mod22a)
## W = 0.97601, p-value = 0.1096

boxplot(SpHeadLen~Population, data=dat1)
```

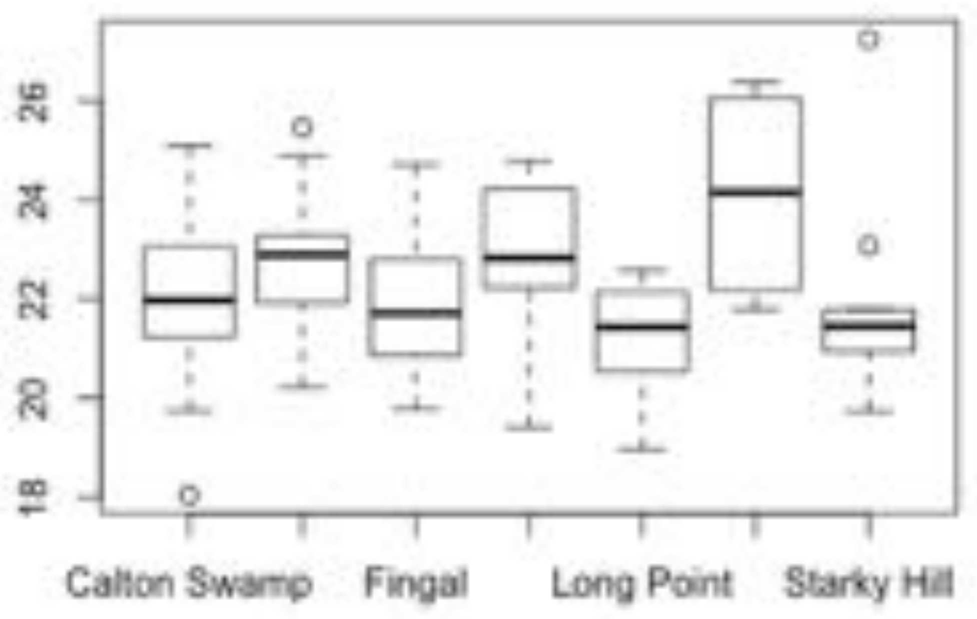


```
(HSD.test(mod22a, "Population")) #requires agricolae package

## $statistics
##       Mean       CV  MSerror      HSD r.harmonic
##   22.19756 6.794144 2.274468 2.080151    9.63028
##
## $parameters
##   Df ntr StudentizedRange alpha  test     name.t
##   78   7         4.280301  0.05 Tukey Population
##
## $means
##                SpHeadLen      std  r   Min   Max
## Calton Swamp  21.94842 1.636348 19 18.04 25.08
## Dereham       22.72067 1.419258 15 20.21 25.45
## Fingal        21.98889 1.444103 18 19.78 24.71
## LaFortune     22.78700 1.558126 10 19.40 24.77
## Long Point    21.17889 1.213656  9 18.96 22.57
## Pond Mills    24.11500 2.276642  4 21.79 26.37
## Starky Hill   21.85636 1.968493 11 19.73 27.24
##
## $comparison
```

```
## NULL
##
## $groups
##              trt    means  M
## 1 Pond Mills   24.11500  a
## 2 LaFortune    22.78700 ab
## 3 Dereham      22.72067 ab
## 4 Fingal       21.98889 ab
## 5 Calton Swamp 21.94842 ab
## 6 Starky Hill  21.85636 ab
## 7 Long Point   21.17889  b
```

```
mod23<- lm(SpHeadPerim~Population+DOY+DOY2+SMI+PC1, data=dat1)
m23 <- dredge(mod23)
```

```
## Fixed term is "(Intercept)"
```

```
subset (m23,delta<2.5)
```

```
## Global model call: lm(formula = SpHeadPerim ~ Population + DOY + DOY2 + SMI + PC1,
##     data = dat1)
## ---
## Model selection table
##    (Intrc)     DOY       DOY2    PC1    SMI df   logLik  AICc delta weight
## 7    53.60          -0.0004562 0.5077        4 -218.497 445.5  0.00  0.119
## 6    60.96 -0.1160             0.5104        4 -218.504 445.5  0.02  0.118
## 3    53.84          -0.0004710               3 -219.642 445.6  0.09  0.114
## 2    61.40 -0.1194                           3 -219.661 445.6  0.13  0.112
## 19   59.64          -0.0005235        -1.954 4 -218.735 446.0  0.48  0.094
## 18   67.97 -0.1325                    -1.942 4 -218.765 446.0  0.54  0.091
## 23   58.51          -0.0005023 0.4467 -1.645 5 -217.855 446.5  0.97  0.073
## 22   66.54 -0.1274             0.4501 -1.632 5 -217.873 446.5  1.01  0.072
## 5    46.16                     0.5370        3 -220.519 447.3  1.84  0.047
## 4   -22.29  1.1980 -0.0051730               4 -219.549 447.6  2.10  0.042
## 1    46.16                                   2 -221.742 447.6  2.14  0.041
## 8    25.17  0.4473 -0.0022120 0.4978        5 -218.484 447.7  2.23  0.039
## 20  -49.02  1.7150 -0.0072580        -2.072 5 -218.543 447.8  2.35  0.037
## Models ranked by AICc(x)
```

```
mod23a<- lm(SpHeadPerim~Population+DOY2+PC1, data=dat1)
summary(mod23a)
```

```
##
## Call:
## lm(formula = SpHeadPerim ~ Population + DOY2 + PC1, data = dat1)
##
## Residuals:
```

```
##      Min      1Q  Median      3Q     Max
## -6.7625 -1.7872 -0.4998  1.7184 10.1248
##
## Coefficients:
##                          Estimate Std. Error t value Pr(>|t|)
## (Intercept)            54.6032420  7.4303501   7.349 1.81e-10 ***
## PopulationDereham       0.6722633  1.1436010   0.588   0.5584
## PopulationFingal        0.9730437  1.1489767   0.847   0.3997
## PopulationLaFortune     0.8274877  1.5543721   0.532   0.5960
## PopulationLong Point   -0.0560264  1.7476709  -0.032   0.9745
## PopulationPond Mills    5.0505339  2.0049676   2.519   0.0138 *
## PopulationStarky Hill   0.8031279  1.2811133   0.627   0.5326
## DOY2                   -0.0005636  0.0004636  -1.215   0.2279
## PC1                     0.7541172  0.4326626   1.743   0.0853 .
## ---
## Signif. codes:  0 '***' 0.001 '**' 0.01 '*' 0.05 '.' 0.1 ' ' 1
##
## Residual standard error: 3.072 on 77 degrees of freedom
## Multiple R-squared:  0.1688, Adjusted R-squared:  0.08239
## F-statistic: 1.954 on 8 and 77 DF,  p-value: 0.06373
```

```
Anova(mod23a)
```

```
## Anova Table (Type II tests)
##
## Response: SpHeadPerim
##            Sum Sq Df F value  Pr(>F)
## Population  83.98  6  1.4832 0.19509
## DOY2        13.94  1  1.4774 0.22790
## PC1         28.67  1  3.0379 0.08533 .
## Residuals  726.64 77
## ---
## Signif. codes:  0 '***' 0.001 '**' 0.01 '*' 0.05 '.' 0.1 ' ' 1
```

```
plot(mod23a) #no departure from normality
```

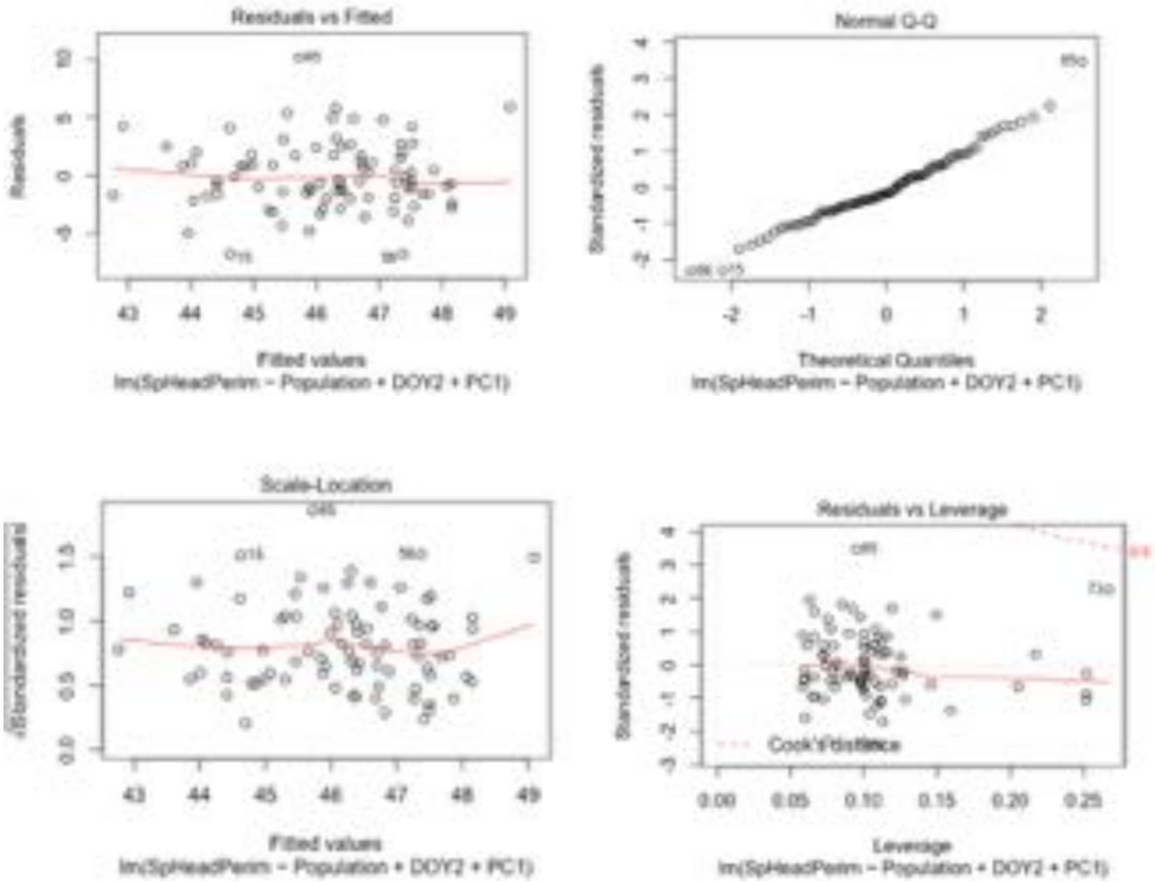


```
shapiro.test(residuals(mod23a)) #no sig departure from normality

##
##  Shapiro-Wilk normality test
##
## data:  residuals(mod23a)
## W = 0.97806, p-value = 0.1511

boxplot(SpHeadPerim~Population, data=dat1)
```

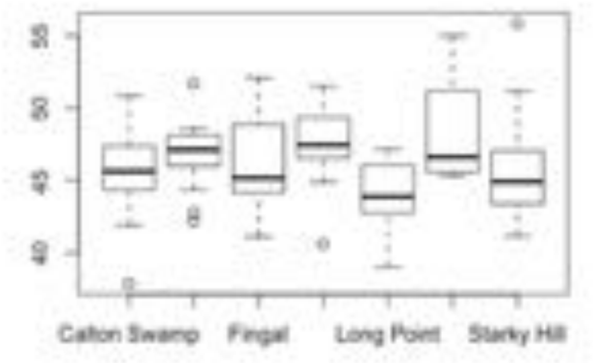


```
(HSD.test(mod23a, "Population")) #requires agricolae package

## $statistics
##       Mean       CV  MSerror      HSD r.harmonic
##   46.16023 6.654957 9.436839 4.238539    9.63028
##
## $parameters
##   Df ntr StudentizedRange alpha  test     name.t
##   77   7          4.28176  0.05 Tukey Population
##
## $means
##              SpHeadPerim      std  r   Min   Max
## Calton Swamp    45.64579 2.975454 19 37.86 50.85
## Dereham         46.71267 2.382995 15 42.18 51.70
## Fingal          46.31556 3.098883 18 41.13 52.08
## LaFortune       47.33400 3.048108 10 40.62 51.49
## Long Point      43.84333 2.620711  9 39.02 47.16
## Pond Mills      48.38500 4.499396  4 45.32 55.00
## Starky Hill     46.06091 4.250942 11 41.16 55.85
##
## $comparison
## NULL
##
## $groups
##            trt    means M
## 1 Pond Mills   48.38500 a
## 2 LaFortune    47.33400 a
## 3 Dereham      46.71267 a
## 4 Fingal       46.31556 a
## 5 Starky Hill  46.06091 a
```

```
## 6 Calton Swamp 45.64579 a
## 7 Long Point   43.84333 a

mod24<- lm(SpTailLen~Population+DOY+DOY2+SMI, data=dat1)
m24 <- dredge(mod24)

## Fixed term is "(Intercept)"

subset (m24,delta<2.5)

## Global model call: lm(formula = SpTailLen ~ Population + DOY + DOY2 + SMI, data =
dat1)
## ---
## Model selection table
##    (Intrc)    DOY    DOY2 Ppltn  SMI df   logLik  AICc delta weight
## 16   -6821 108.50 -0.4283     + -8.3 11 -306.724 639.0  0.00  0.559
## 8    -6282  99.66 -0.3931     +      10 -308.280 639.5  0.48  0.441
## Models ranked by AICc(x)

summary(mod24)

##
## Call:
## lm(formula = SpTailLen ~ Population + DOY + DOY2 + SMI, data = dat1)
##
## Residuals:
##      Min       1Q   Median       3Q      Max
## -19.7816  -4.6346   0.3833   5.8861  19.4683
##
## Coefficients:
##                          Estimate Std. Error t value Pr(>|t|)
## (Intercept)            -6820.9128  2077.3086  -3.284  0.00155 **
## PopulationDereham          6.0918     3.6052   1.690  0.09518 .
## PopulationFingal           8.3352     3.3915   2.458  0.01626 *
## PopulationLaFortune       23.9291    10.0032   2.392  0.01922 *
## PopulationLong Point      27.7708    10.2040   2.722  0.00805 **
## PopulationPond Mills      12.3811     8.1698   1.515  0.13380
## PopulationStarky Hill     -0.8443     3.7668  -0.224  0.82324
## DOY                      108.5407    32.6983   3.319  0.00139 **
## DOY2                      -0.4283     0.1284  -3.334  0.00132 **
## SMI                       -8.2996     4.9594  -1.673  0.09834 .
## ---
## Signif. codes:  0 '***' 0.001 '**' 0.01 '*' 0.05 '.' 0.1 ' ' 1
##
## Residual standard error: 9.111 on 76 degrees of freedom
## Multiple R-squared:  0.359,  Adjusted R-squared:  0.283
## F-statistic: 4.728 on 9 and 76 DF,  p-value: 5.33e-05
```

```
(m24 <- Anova(mod24))

## Anova Table (Type II tests)
##
## Response: SpTailLen
##             Sum Sq Df F value   Pr(>F)
## Population 1484.1  6  2.9801 0.011443 *
## DOY         914.6  1 11.0188 0.001387 **
## DOY2        922.9  1 11.1189 0.001323 **
## SMI         232.5  1  2.8006 0.098342 .
## Residuals  6308.1 76
## ---
## Signif. codes:  0 '***' 0.001 '**' 0.01 '*' 0.05 '.' 0.1 ' ' 1

m24[5,1]/sum(m24[,1]) #unexplained variation from this model

## [1] 0.6396277

plot(mod24) #no departure from normality
```

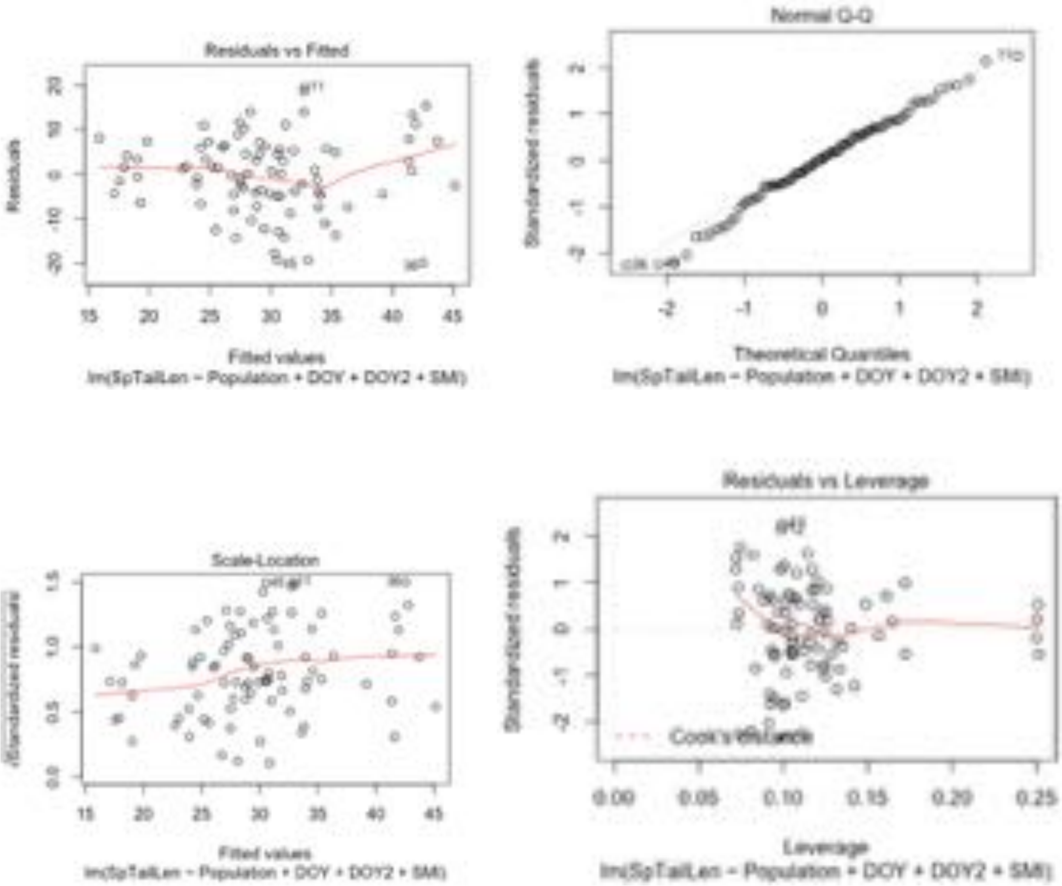


```
shapiro.test(residuals(mod24)) #no sig departure from normality

##
##  Shapiro-Wilk normality test
```

```
##
## data:  residuals(mod24)
## W = 0.98876, p-value = 0.6701
```

4.4.2 Sperm abnormalities compared across populations

Analyses for Fig 4 (right) with some results reported in the text; comparison of models mod31b and mod31c reported in text to determine effect of Population

```
#All abnormalities
mod31 <- glm(cbind(Abnormal,noAb)~Population,family=binomial, data=dat3)
Anova(mod31)

## Analysis of Deviance Table (Type II tests)
##
## Response: cbind(Abnormal, noAb)
##            LR Chisq Df Pr(>Chisq)
## Population    27.97  6  9.519e-05 ***
## ---
## Signif. codes:  0 '***' 0.001 '**' 0.01 '*' 0.05 '.' 0.1 ' ' 1

summary(mod31) #serious overdispersion indicated

##
## Call:
## glm(formula = cbind(Abnormal, noAb) ~ Population, family = binomial,
##     data = dat3)
##
## Deviance Residuals:
##     Min       1Q   Median       3Q      Max
## -6.1565  -1.8655  -0.1552   1.7570   7.9941
##
## Coefficients:
##                          Estimate Std. Error z value Pr(>|z|)
## (Intercept)              0.176857   0.050039   3.534 0.000409 ***
## PopulationDereham        0.234372   0.075550   3.102 0.001921 **
## PopulationFingal         0.037345   0.072009   0.519 0.604031
## PopulationLaFortune      0.346216   0.100261   3.453 0.000554 ***
## PopulationLong Point    -0.007059   0.092144  -0.077 0.938931
## PopulationPond Mills    -0.184237   0.131394  -1.402 0.160862
## PopulationStarky Hill   -0.011343   0.084395  -0.134 0.893087
## ---
## Signif. codes:  0 '***' 0.001 '**' 0.01 '*' 0.05 '.' 0.1 ' ' 1
##
## (Dispersion parameter for binomial family taken to be 1)
##
```

```
##     Null deviance: 725.89  on 84  degrees of freedom
## Residual deviance: 697.92  on 78  degrees of freedom
## AIC: 1104.2
##
## Number of Fisher Scoring iterations: 4
```

```r
plot(mod31)
```

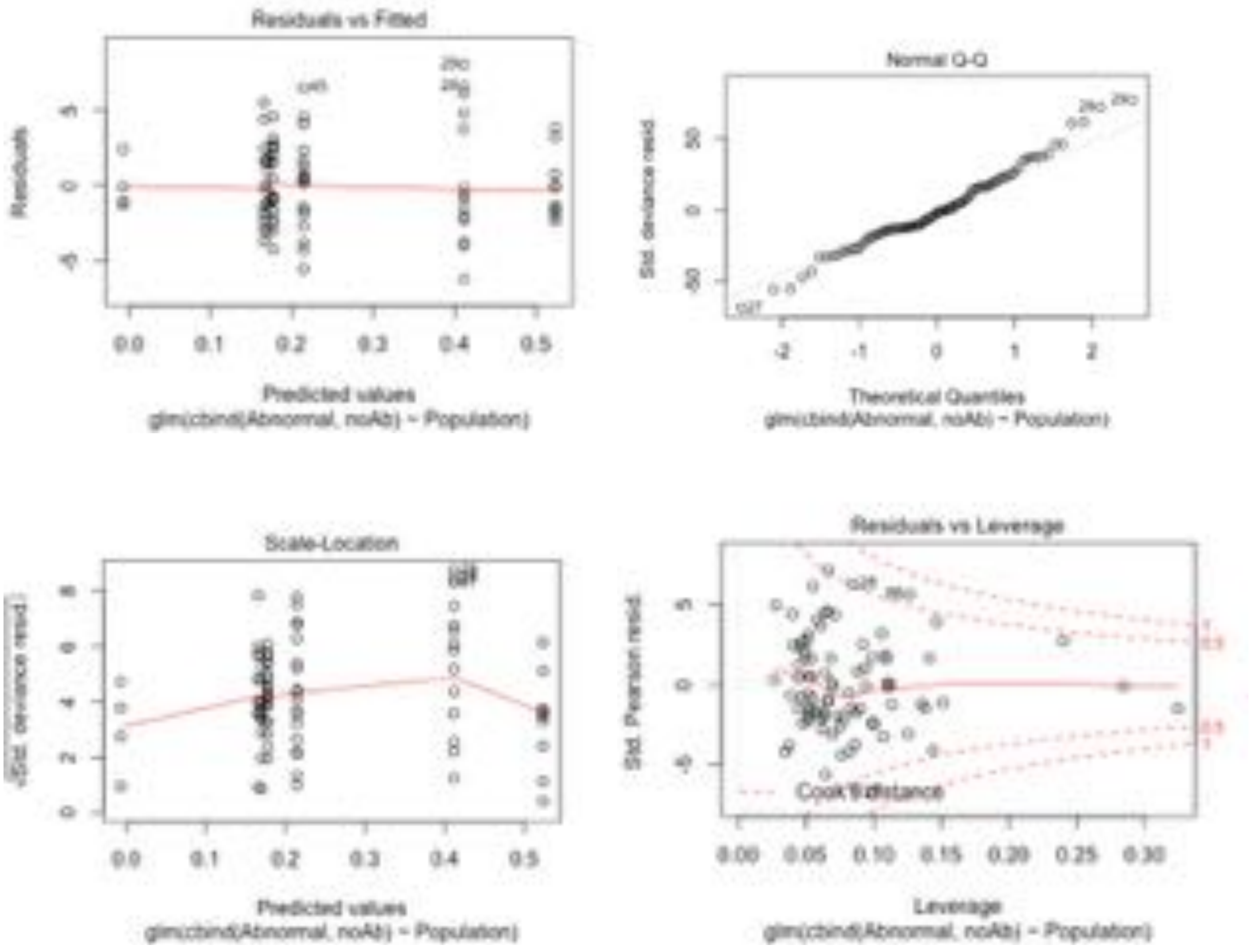


```r
shapiro.test(residuals(mod31))

##
##  Shapiro-Wilk normality test
##
## data:  residuals(mod31)
## W = 0.97809, p-value = 0.1571

mod31a <- glm.binomial.disp(mod31) #correct for overdispersion

##
## Binomial overdispersed logit model fitting...
## Iter.  1  phi: 0.09170989
## Iter.  2  phi: 0.0913252
## Iter.  3  phi: 0.0913242
## Iter.  4  phi: 0.0913242
## Converged after 4 iterations.
## Estimated dispersion parameter: 0.0913242
##
```

```
## Call:
## glm(formula = cbind(Abnormal, noAb) ~ Population, family = binomial,
##     data = dat3, weights = disp.weights)
##
## Deviance Residuals:
##     Min       1Q   Median       3Q      Max
## -2.0244  -0.6942  -0.1976   0.6438   2.8123
##
## Coefficients:
##                         Estimate Std. Error z value Pr(>|z|)
## (Intercept)             0.212054   0.147695   1.436    0.151
## PopulationDereham       0.323110   0.230671   1.401    0.161
## PopulationFingal        0.008686   0.211968   0.041    0.967
## PopulationLaFortune     0.229263   0.259601   0.883    0.377
## PopulationLong Point  -0.045806   0.261228  -0.175    0.861
## PopulationPond Mills  -0.222413   0.357096  -0.623    0.533
## PopulationStarky Hill -0.073968   0.244114  -0.303    0.762
##
## (Dispersion parameter for binomial family taken to be 1)
##
##     Null deviance: 89.965  on 84  degrees of freedom
## Residual deviance: 85.340  on 78  degrees of freedom
## AIC: 149.23
##
## Number of Fisher Scoring iterations: 4
```

```
mod31b <- glm(cbind(Abnormal, noAb)~1, family=binomial, data=dat3)
mod31c <- glm.binomial.disp(mod31b)
```

```
##
## Binomial overdispersed logit model fitting...
## Iter.  1  phi: 0.08751271
## Iter.  2  phi: 0.08813602
## Iter.  3  phi: 0.0881383
## Iter.  4  phi: 0.08813831
## Converged after 4 iterations.
## Estimated dispersion parameter: 0.08813831
##
## Call:
## glm(formula = cbind(Abnormal, noAb) ~ 1, family = binomial, data = dat3,
##     weights = disp.weights)
##
## Deviance Residuals:
##     Min       1Q   Median       3Q      Max
```

```
## -1.8717  -0.6843  -0.1026   0.5085   3.1655
##
## Coefficients:
##             Estimate Std. Error z value Pr(>|z|)
## (Intercept)  0.26705    0.06937    3.85 0.000118 ***
## ---
## Signif. codes:  0 '***' 0.001 '**' 0.01 '*' 0.05 '.' 0.1 ' ' 1
##
## (Dispersion parameter for binomial family taken to be 1)
##
##     Null deviance: 92.76  on 84  degrees of freedom
## Residual deviance: 92.76  on 84  degrees of freedom
## AIC: 146.2
##
## Number of Fisher Scoring iterations: 4
```

```
anova(mod31a, mod31c, test="Chisq")
```

```
## Analysis of Deviance Table
##
## Model 1: cbind(Abnormal, noAb) ~ Population
## Model 2: cbind(Abnormal, noAb) ~ 1
##   Resid. Df Resid. Dev Df Deviance Pr(>Chi)
## 1        78      85.34
## 2        84      92.76 -6  -7.4208   0.2837
```

```
mod32 <- glm(cbind(CytoDrop, noCD)~Population, family=binomial, data=dat3)
summary(mod32) #serious overdispersion
```

```
##
## Call:
## glm(formula = cbind(CytoDrop, noCD) ~ Population, family = binomial,
##     data = dat3)
##
## Deviance Residuals:
##     Min       1Q   Median       3Q      Max
## -8.0111  -2.0671  -0.2672   1.9246   9.9091
##
## Coefficients:
##                       Estimate Std. Error z value Pr(>|z|)
## (Intercept)           -0.29529    0.05039  -5.860 4.62e-09 ***
## PopulationDereham      0.23383    0.07493   3.121  0.00180 **
## PopulationFingal      -0.02416    0.07251  -0.333  0.73899
## PopulationLaFortune    0.39768    0.09804   4.056 4.99e-05 ***
## PopulationLong Point  -0.06210    0.09314  -0.667  0.50491
```

```
## PopulationPond Mills  -0.43685    0.13917  -3.139  0.00169 **
## PopulationStarky Hill  0.17128    0.08452   2.027  0.04271 *
## ---
## Signif. codes:  0 '***' 0.001 '**' 0.01 '*' 0.05 '.' 0.1 ' ' 1
##
## (Dispersion parameter for binomial family taken to be 1)
##
##     Null deviance: 814.73  on 84  degrees of freedom
## Residual deviance: 764.49  on 78  degrees of freedom
## AIC: 1173.2
##
## Number of Fisher Scoring iterations: 4
```

```
mod32a <- glm.binomial.disp(mod32) #correct for overdispersion
```

```
##
## Binomial overdispersed logit model fitting...
## Iter.  1  phi: 0.1037045
## Iter.  2  phi: 0.1013044
## Iter.  3  phi: 0.1013134
## Iter.  4  phi: 0.1013134
## Converged after 4 iterations.
## Estimated dispersion parameter: 0.1013134
##
## Call:
## glm(formula = cbind(CytoDrop, noCD) ~ Population, family = binomial,
##     data = dat3, weights = disp.weights)
##
## Deviance Residuals:
##      Min        1Q    Median        3Q       Max
## -2.73342  -0.71226  -0.06861   0.64041   3.13376
##
## Coefficients:
##                         Estimate Std. Error z value Pr(>|z|)
## (Intercept)             -0.25290    0.15501  -1.631    0.103
## PopulationDereham        0.23799    0.23687   1.005    0.315
## PopulationFingal        -0.07504    0.22302  -0.336    0.737
## PopulationLaFortune      0.28059    0.26720   1.050    0.294
## PopulationLong Point    -0.12007    0.27621  -0.435    0.664
## PopulationPond Mills    -0.49574    0.39565  -1.253    0.210
## PopulationStarky Hill    0.10517    0.25581   0.411    0.681
##
## (Dispersion parameter for binomial family taken to be 1)
##
```

```
##     Null deviance: 89.198  on 84  degrees of freedom
## Residual deviance: 83.296  on 78  degrees of freedom
## AIC: 143.52
##
## Number of Fisher Scoring iterations: 3
```

```
mod32b <- glm(cbind(CytoDrop, noCD)~1, family=binomial, data=dat3)
mod32c <- glm.binomial.disp(mod32b)
```

```
##
## Binomial overdispersed logit model fitting...
## Iter.  1  phi: 0.1027672
## Iter.  2  phi: 0.1011135
## Iter.  3  phi: 0.1011193
## Iter.  4  phi: 0.1011193
## Converged after 4 iterations.
## Estimated dispersion parameter: 0.1011193
##
## Call:
## glm(formula = cbind(CytoDrop, noCD) ~ 1, family = binomial, data = dat3,
##     weights = disp.weights)
##
## Deviance Residuals:
##     Min       1Q   Median       3Q      Max
## -2.4609  -0.7654  -0.1050   0.5418   3.4106
##
## Coefficients:
##             Estimate Std. Error z value Pr(>|z|)
## (Intercept) -0.21754    0.07345  -2.962  0.00306 **
## ---
## Signif. codes:  0 '***' 0.001 '**' 0.01 '*' 0.05 '.' 0.1 ' ' 1
##
## (Dispersion parameter for binomial family taken to be 1)
##
##     Null deviance: 89.35  on 84  degrees of freedom
## Residual deviance: 89.35  on 84  degrees of freedom
## AIC: 137.65
##
## Number of Fisher Scoring iterations: 3
```

```
anova(mod32a, mod32c, test="Chisq")
```

```
## Analysis of Deviance Table
##
## Model 1: cbind(CytoDrop, noCD) ~ Population
```

```
## Model 2: cbind(CytoDrop, noCD) ~ 1
##   Resid. Df Resid. Dev Df Deviance Pr(>Chi)
## 1        78     83.296
## 2        84     89.350 -6   -6.054   0.4172

#plot by population
boxplot(Pcd~Population, data=dat3)
```

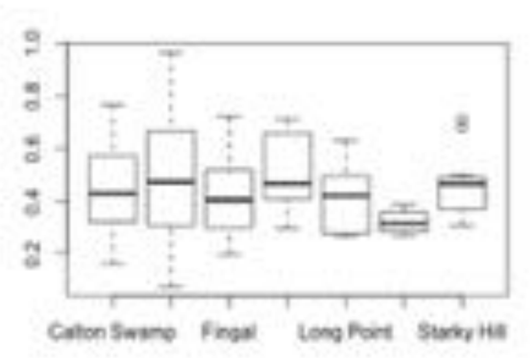


```
mod33 <- glm(cbind(NoTail, no0t)~Population, family=binomial, data=dat3)
summary(mod33) #serious overdispersion

##
## Call:
## glm(formula = cbind(NoTail, no0t) ~ Population, family = binomial,
##     data = dat3)
##
## Deviance Residuals:
##     Min       1Q   Median       3Q      Max
## -4.2272  -1.8532  -0.6853   0.9956   8.3421
##
## Coefficients:
##                          Estimate Std. Error z value Pr(>|z|)
## (Intercept)              -1.67029    0.06826 -24.469  < 2e-16 ***
## PopulationDereham        -0.90629    0.12789  -7.087 1.37e-12 ***
## PopulationFingal         -0.50431    0.10905  -4.625 3.75e-06 ***
## PopulationLaFortune      -0.13594    0.13861  -0.981    0.327
## PopulationLong Point     -0.78913    0.15856  -4.977 6.46e-07 ***
## PopulationPond Mills     -0.49056    0.21092  -2.326    0.020 *
## PopulationStarky Hill -0.56839    0.13354  -4.256 2.08e-05 ***
## ---
## Signif. codes:  0 '***' 0.001 '**' 0.01 '*' 0.05 '.' 0.1 ' ' 1
##
## (Dispersion parameter for binomial family taken to be 1)
```

```
##
##     Null deviance: 532.71  on 84  degrees of freedom
## Residual deviance: 459.64  on 78  degrees of freedom
## AIC: 769.96
##
## Number of Fisher Scoring iterations: 5
```

```
mod33a <- glm.binomial.disp(mod33) #correct for overdispersion
```

```
##
## Binomial overdispersed logit model fitting...
## Iter.  1  phi: 0.06918576
## Iter.  2  phi: 0.07396876
## Iter.  3  phi: 0.07402569
## Iter.  4  phi: 0.07402633
## Iter.  5  phi: 0.07402634
## Converged after 5 iterations.
## Estimated dispersion parameter: 0.07402634
##
## Call:
## glm(formula = cbind(NoTail, no0t) ~ Population, family = binomial,
##     data = dat3, weights = disp.weights)
##
## Deviance Residuals:
##     Min       1Q   Median       3Q      Max
## -1.7133  -0.7341  -0.2768   0.2517   3.1299
##
## Coefficients:
##                         Estimate Std. Error z value Pr(>|z|)
## (Intercept)              -1.6202     0.1805  -8.976   <2e-16 ***
## PopulationDereham        -0.8368     0.3411  -2.453   0.0141 *
## PopulationFingal         -0.5361     0.2894  -1.852   0.0640 .
## PopulationLaFortune      -0.2787     0.3367  -0.828   0.4079
## PopulationLong Point     -0.8212     0.4041  -2.032   0.0421 *
## PopulationPond Mills     -0.4653     0.5079  -0.916   0.3596
## PopulationStarky Hill    -0.6612     0.3548  -1.864   0.0624 .
## ---
## Signif. codes:  0 '***' 0.001 '**' 0.01 '*' 0.05 '.' 0.1 ' ' 1
##
## (Dispersion parameter for binomial family taken to be 1)
##
##     Null deviance: 81.141  on 84  degrees of freedom
## Residual deviance: 71.368  on 78  degrees of freedom
## AIC: 130.56
```

```
##
## Number of Fisher Scoring iterations: 5
```

```
summary(mod33a)
```

```
##
## Call:
## glm(formula = cbind(NoTail, no0t) ~ Population, family = binomial,
##     data = dat3, weights = disp.weights)
##
## Deviance Residuals:
##     Min       1Q   Median       3Q      Max
## -1.7133  -0.7341  -0.2768   0.2517   3.1299
##
## Coefficients:
##                         Estimate Std. Error z value Pr(>|z|)
## (Intercept)              -1.6202     0.1805  -8.976   <2e-16 ***
## PopulationDereham        -0.8368     0.3411  -2.453   0.0141 *
## PopulationFingal         -0.5361     0.2894  -1.852   0.0640 .
## PopulationLaFortune      -0.2787     0.3367  -0.828   0.4079
## PopulationLong Point     -0.8212     0.4041  -2.032   0.0421 *
## PopulationPond Mills     -0.4653     0.5079  -0.916   0.3596
## PopulationStarky Hill    -0.6612     0.3548  -1.864   0.0624 .
## ---
## Signif. codes:  0 '***' 0.001 '**' 0.01 '*' 0.05 '.' 0.1 ' ' 1
##
## (Dispersion parameter for binomial family taken to be 1)
##
##     Null deviance: 81.141  on 84  degrees of freedom
## Residual deviance: 71.368  on 78  degrees of freedom
## AIC: 130.56
##
## Number of Fisher Scoring iterations: 5
```

```
mod33b <- glm(cbind(NoTail, no0t)~1, family=binomial, data=dat3)
mod33c <- glm.binomial.disp(mod33b)
```

```
##
## Binomial overdispersed logit model fitting...
## Iter.  1  phi: 0.07975372
## Iter.  2  phi: 0.08211482
## Iter.  3  phi: 0.08211891
## Iter.  4  phi: 0.08211892
## Converged after 4 iterations.
## Estimated dispersion parameter: 0.08211892
```

```
##
## Call:
## glm(formula = cbind(NoTail, no0t) ~ 1, family = binomial, data = dat3,
##     weights = disp.weights)
##
## Deviance Residuals:
##     Min       1Q   Median       3Q      Max
## -1.6067  -0.6999  -0.3011   0.4175   3.6099
##
## Coefficients:
##             Estimate Std. Error z value Pr(>|z|)
## (Intercept)  -2.0617     0.1054  -19.57   <2e-16 ***
## ---
## Signif. codes:  0 '***' 0.001 '**' 0.01 '*' 0.05 '.' 0.1 ' ' 1
##
## (Dispersion parameter for binomial family taken to be 1)
##
##     Null deviance: 74.318  on 84  degrees of freedom
## Residual deviance: 74.318  on 84  degrees of freedom
## AIC: 117.72
##
## Number of Fisher Scoring iterations: 5
```

```
anova(mod33a, mod33c, test="Chisq")
```

```
## Analysis of Deviance Table
##
## Model 1: cbind(NoTail, no0t) ~ Population
## Model 2: cbind(NoTail, no0t) ~ 1
##   Resid. Df Resid. Dev Df Deviance Pr(>Chi)
## 1        78     71.368
## 2        84     74.318 -6  -2.9508    0.815
```

```
#plot by population
boxplot(P0tails~Population, data=dat3)
```

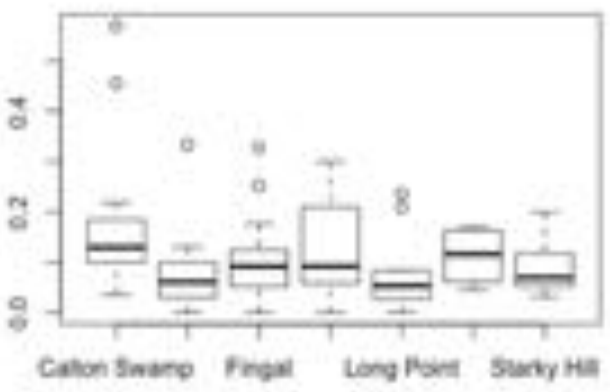


```
#no head
mod34 <- glm(cbind(NoHead, no0H)~Population,family=binomial, weights= TotalSperm,
data=dat3)
summary(mod34) #serious overdispersion
```

```
##
## Call:
## glm(formula = cbind(NoHead, no0H) ~ Population, family = binomial,
##     data = dat3, weights = TotalSperm)
##
## Deviance Residuals:
##     Min       1Q   Median       3Q      Max
## -18.694  -10.053   -5.420    5.346   25.228
##
## Coefficients:
##                         Estimate Std. Error  z value Pr(>|z|)
## (Intercept)             -4.26675    0.02271 -187.921  < 2e-16 ***
## PopulationDereham       -0.98350    0.04370  -22.505  < 2e-16 ***
## PopulationFingal         0.20993    0.03109    6.753 1.45e-11 ***
## PopulationLaFortune     -0.29255    0.05778   -5.064 4.12e-07 ***
## PopulationLong Point    -0.35906    0.05035   -7.131 9.94e-13 ***
## PopulationPond Mills    -0.50105    0.08141   -6.155 7.52e-10 ***
## PopulationStarky Hill   -0.53426    0.04704  -11.357  < 2e-16 ***
## ---
## Signif. codes:  0 '***' 0.001 '**' 0.01 '*' 0.05 '.' 0.1 ' ' 1
##
## (Dispersion parameter for binomial family taken to be 1)
##
##     Null deviance: 10431.7  on 84  degrees of freedom
```

```
## Residual deviance:  9348.7  on 78  degrees of freedom
## AIC: 17994
##
## Number of Fisher Scoring iterations: 5
```

```
mod34a <- glm.binomial.disp(mod34) #correct for overdispersion
```

```
##
## Binomial overdispersed logit model fitting...
## Iter.  1  phi: 1.583222
## Iter.  2  phi: 0.01037556
## Iter.  3  phi: 0.009497971
## Iter.  4  phi: 0.009461119
## Iter.  5  phi: 0.009459504
## Iter.  6  phi: 0.009459433
## Iter.  7  phi: 0.00945943
## Converged after 7 iterations.
## Estimated dispersion parameter: 0.00945943
##
## Call:
## glm(formula = cbind(NoHead, no0H) ~ Population, family = binomial,
##     data = dat3, weights = disp.weights)
##
## Deviance Residuals:
##     Min       1Q   Median       3Q      Max
## -1.4083  -0.8706  -0.6428   0.4364   2.1604
##
## Coefficients:
##                         Estimate Std. Error z value Pr(>|z|)
## (Intercept)              -4.2862     0.2899 -14.787   <2e-16 ***
## PopulationDereham        -0.8729     0.5876  -1.485    0.137
## PopulationFingal          0.3591     0.3837   0.936    0.349
## PopulationLaFortune      -0.2488     0.5874  -0.424    0.672
## PopulationLong Point     -0.2432     0.5696  -0.427    0.669
## PopulationPond Mills     -0.6799     0.9874  -0.689    0.491
## PopulationStarky Hill    -0.5444     0.5844  -0.932    0.352
## ---
## Signif. codes:  0 '***' 0.001 '**' 0.01 '*' 0.05 '.' 0.1 ' ' 1
##
## (Dispersion parameter for binomial family taken to be 1)
##
##     Null deviance: 78.418  on 84  degrees of freedom
## Residual deviance: 70.948  on 78  degrees of freedom
## AIC: 143.48
```

```
##
## Number of Fisher Scoring iterations: 5
```

```
summary(mod34a)
```

```
##
## Call:
## glm(formula = cbind(NoHead, no0H) ~ Population, family = binomial,
##     data = dat3, weights = disp.weights)
##
## Deviance Residuals:
##     Min       1Q   Median       3Q      Max
## -1.4083  -0.8706  -0.6428   0.4364   2.1604
##
## Coefficients:
##                        Estimate Std. Error z value Pr(>|z|)
## (Intercept)             -4.2862     0.2899 -14.787   <2e-16 ***
## PopulationDereham       -0.8729     0.5876  -1.485    0.137
## PopulationFingal         0.3591     0.3837   0.936    0.349
## PopulationLaFortune     -0.2488     0.5874  -0.424    0.672
## PopulationLong Point    -0.2432     0.5696  -0.427    0.669
## PopulationPond Mills    -0.6799     0.9874  -0.689    0.491
## PopulationStarky Hill   -0.5444     0.5844  -0.932    0.352
## ---
## Signif. codes:  0 '***' 0.001 '**' 0.01 '*' 0.05 '.' 0.1 ' ' 1
##
## (Dispersion parameter for binomial family taken to be 1)
##
##     Null deviance: 78.418  on 84  degrees of freedom
## Residual deviance: 70.948  on 78  degrees of freedom
## AIC: 143.48
##
## Number of Fisher Scoring iterations: 5
```

```
mod34b <- glm(cbind(NoHead, no0H)~1,family=binomial, weights= TotalSperm, data=dat3)
mod34c <- glm.binomial.disp(mod34b) #correct for overdispersion
```

```
##
## Binomial overdispersed logit model fitting...
## Iter.  1  phi: 1.669103
## Iter.  2  phi: 0.00982149
## Iter.  3  phi: 0.009298202
## Iter.  4  phi: 0.009285785
## Iter.  5  phi: 0.009285483
## Iter.  6  phi: 0.009285476
```

```
## Converged after 6 iterations.
## Estimated dispersion parameter: 0.009285476
##
## Call:
## glm(formula = cbind(NoHead, no0H) ~ 1, family = binomial, data = dat3,
##     weights = disp.weights)
##
## Deviance Residuals:
##     Min       1Q   Median       3Q      Max
## -1.1849  -1.0209  -0.2280   0.2365   2.5235
##
## Coefficients:
##             Estimate Std. Error z value Pr(>|z|)
## (Intercept)  -4.4244     0.1491  -29.67   <2e-16 ***
## ---
## Signif. codes:  0 '***' 0.001 '**' 0.01 '*' 0.05 '.' 0.1 ' ' 1
##
## (Dispersion parameter for binomial family taken to be 1)
##
##     Null deviance: 79.01  on 84  degrees of freedom
## Residual deviance: 79.01  on 84  degrees of freedom
## AIC: 140
##
## Number of Fisher Scoring iterations: 5
```

```
anova(mod34a, mod34c, test="Chisq")
```

```
## Analysis of Deviance Table
##
## Model 1: cbind(NoHead, no0H) ~ Population
## Model 2: cbind(NoHead, no0H) ~ 1
##   Resid. Df Resid. Dev Df Deviance Pr(>Chi)
## 1        78     70.948
## 2        84     79.010 -6  -8.0621   0.2336
```

```
#plot by population
boxplot(P0heads~Population, data=dat3)
```

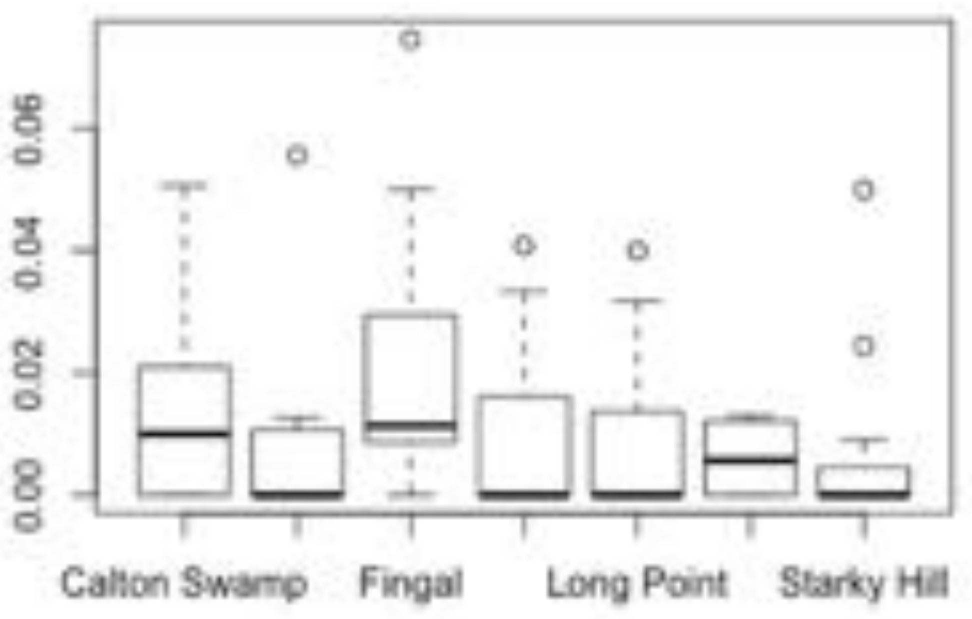


```
#two tails
mod35 <- glm(cbind(TwoTails, no2t)~Population, family=binomial, data=dat3)
summary(mod35) #somewhat overdispersed

##
## Call:
## glm(formula = cbind(TwoTails, no2t) ~ Population, family = binomial,
##     data = dat3)
##
## Deviance Residuals:
##     Min       1Q   Median       3Q      Max
## -2.0711  -0.9667  -0.6183   0.4936   4.5060
##
## Coefficients:
##                          Estimate Std. Error z value Pr(>|z|)
## (Intercept)               -5.0752     0.3172 -15.999  < 2e-16 ***
## PopulationDereham         -0.7071     0.5928  -1.193 0.232924
## PopulationFingal           1.2746     0.3628   3.513 0.000442 ***
## PopulationLaFortune        0.1282     0.5936   0.216 0.829036
## PopulationLong Point       1.2235     0.4166   2.937 0.003317 **
## PopulationPond Mills       2.0872     0.4259   4.900 9.58e-07 ***
```

```
## PopulationStarky Hill  -1.6945     1.0497  -1.614 0.106459
## ---
## Signif. codes:  0 '***' 0.001 '**' 0.01 '*' 0.05 '.' 0.1 ' ' 1
##
## (Dispersion parameter for binomial family taken to be 1)
##
##     Null deviance: 203.42  on 84  degrees of freedom
## Residual deviance: 139.35  on 78  degrees of freedom
## AIC: 240.21
##
## Number of Fisher Scoring iterations: 6
```

```
mod35a <- glm.binomial.disp(mod35) #correct for overdispersion
```

```
##
## Binomial overdispersed logit model fitting...
## Iter.  1  phi: 0.01574329
## Iter.  2  phi: 0.01652476
## Iter.  3  phi: 0.01653814
## Iter.  4  phi: 0.01653836
## Iter.  5  phi: 0.01653837
## Converged after 5 iterations.
## Estimated dispersion parameter: 0.01653837
##
## Call:
## glm(formula = cbind(TwoTails, no2t) ~ Population, family = binomial,
##     data = dat3, weights = disp.weights)
##
## Deviance Residuals:
##     Min       1Q   Median       3Q      Max
## -1.5410  -0.6771  -0.4399   0.2951   3.0647
##
## Coefficients:
##                         Estimate Std. Error z value Pr(>|z|)
## (Intercept)             -4.99887    0.47418 -10.542   <2e-16 ***
## PopulationDereham       -0.80181    0.94316  -0.850   0.3953
## PopulationFingal         1.22958    0.54504   2.256   0.0241 *
## PopulationLaFortune      0.09985    0.83396   0.120   0.9047
## PopulationLong Point     1.23111    0.61254   2.010   0.0444 *
## PopulationPond Mills     1.98893    0.63362   3.139   0.0017 **
## PopulationStarky Hill -1.77653    1.60222  -1.109   0.2675
## ---
## Signif. codes:  0 '***' 0.001 '**' 0.01 '*' 0.05 '.' 0.1 ' ' 1
##
```

```
## (Dispersion parameter for binomial family taken to be 1)
##
##     Null deviance: 91.181  on 84  degrees of freedom
## Residual deviance: 63.206  on 78  degrees of freedom
## AIC: 115.65
##
## Number of Fisher Scoring iterations: 6
```

```
mod35b <- glm(cbind(TwoTails, no2t)~1, family=binomial, data=dat3)
mod35c <- glm.binomial.disp(mod35b)
```

```
##
## Binomial overdispersed logit model fitting...
## Iter.  1  phi: 0.03622566
## Iter.  2  phi: 0.03720586
## Iter.  3  phi: 0.03721006
## Iter.  4  phi: 0.03721008
## Converged after 4 iterations.
## Estimated dispersion parameter: 0.03721008
##
## Call:
## glm(formula = cbind(TwoTails, no2t) ~ 1, family = binomial, data = dat3,
##     weights = disp.weights)
##
## Deviance Residuals:
##      Min        1Q    Median        3Q       Max
## -0.77026  -0.71327  -0.68694  -0.01799   2.93964
##
## Coefficients:
##             Estimate Std. Error z value Pr(>|z|)
## (Intercept)  -4.3705     0.2185     -20   <2e-16 ***
## ---
## Signif. codes:  0 '***' 0.001 '**' 0.01 '*' 0.05 '.' 0.1 ' ' 1
##
## (Dispersion parameter for binomial family taken to be 1)
##
##     Null deviance: 54.537  on 84  degrees of freedom
## Residual deviance: 54.537  on 84  degrees of freedom
## AIC: 79.432
##
## Number of Fisher Scoring iterations: 5
```

```
anova(mod35a, mod35c, test="Chisq")
```

```
## Analysis of Deviance Table
##
## Model 1: cbind(TwoTails, no2t) ~ Population
## Model 2: cbind(TwoTails, no2t) ~ 1
##   Resid. Df Resid. Dev Df Deviance Pr(>Chi)
## 1        78     63.206
## 2        84     54.537 -6   8.6692

#plot by population
boxplot(P2tails~Population, data=dat3)
```

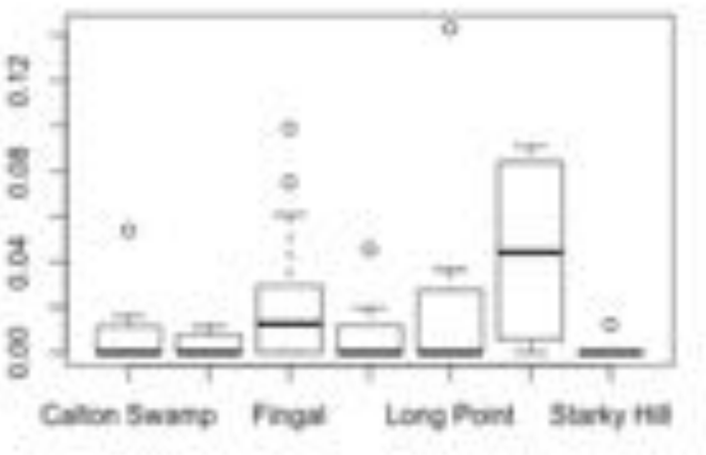


```
#two heads
mod36 <- glm(cbind(TwoHeads, no2H)~Population, family=binomial, data=dat3)
summary(mod36) #underdispersed but OK

##
## Call:
## glm(formula = cbind(TwoHeads, no2H) ~ Population, family = binomial,
##     data = dat3)
##
## Deviance Residuals:
##      Min         1Q     Median         3Q        Max
## -0.80663  -0.00003  -0.00003  -0.00003   1.99198
##
## Coefficients:
##                          Estimate Std. Error z value Pr(>|z|)
## (Intercept)            -2.613e+01  7.130e+03  -0.004    0.997
## PopulationDereham      -4.783e-02  1.081e+04   0.000    1.000
## PopulationFingal        1.881e+01  7.130e+03   0.003    0.998
```

```
## PopulationLaFortune    4.121e-01  1.210e+04   0.000    1.000
## PopulationLong Point   1.146e-01  1.262e+04   0.000    1.000
## PopulationPond Mills   2.053e+01  7.130e+03   0.003    0.998
## PopulationStarky Hill  7.233e-02  1.175e+04   0.000    1.000
##
## (Dispersion parameter for binomial family taken to be 1)
##
##     Null deviance: 15.0768  on 84  degrees of freedom
## Residual deviance:  8.3872  on 78  degrees of freedom
## AIC: 26.362
##
## Number of Fisher Scoring iterations: 21
```

```
mod36b <- glm(cbind(TwoHeads, no2H)~1, family=binomial, data=dat3)
anova(mod36, mod36b, test="Chisq")
```

```
## Analysis of Deviance Table
##
## Model 1: cbind(TwoHeads, no2H) ~ Population
## Model 2: cbind(TwoHeads, no2H) ~ 1
##   Resid. Df Resid. Dev Df Deviance Pr(>Chi)
## 1        78     8.3872
## 2        84    15.0768 -6  -6.6896   0.3505
```

```
#plot by population
boxplot(P2heads~Population, data=dat3)
```

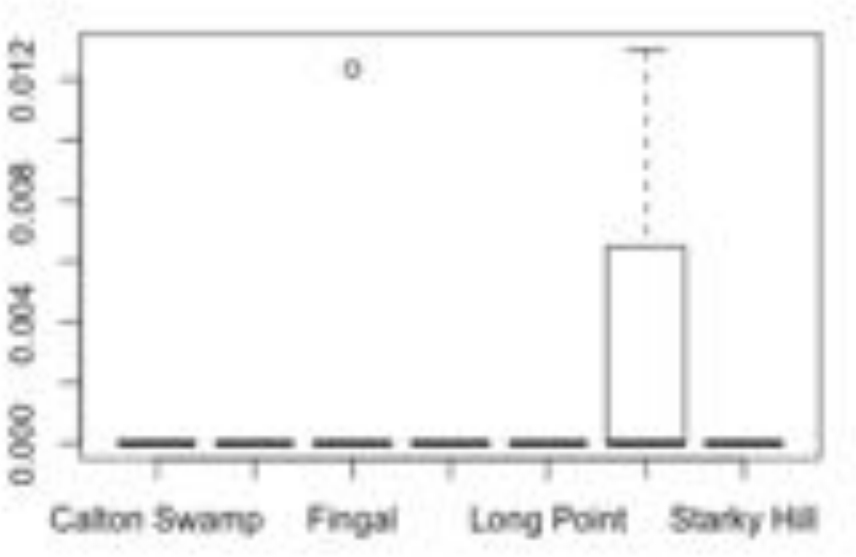


```
#other abnormalities
mod37 <- glm(cbind(Other, noOTH)~Population, family=binomial, data=dat3)
summary(mod37) #underdispersed but OK

##
## Call:
## glm(formula = cbind(Other, noOTH) ~ Population, family = binomial,
##     data = dat3)
##
## Deviance Residuals:
##      Min        1Q    Median        3Q       Max
## -0.77588  -0.54214  -0.00005  -0.00004   2.15222
##
## Coefficients:
##                           Estimate Std. Error z value Pr(>|z|)
## (Intercept)               -5.99521    0.50062 -11.976   <2e-16 ***
## PopulationDereham        -19.18069 4925.21201  -0.004    0.997
## PopulationFingal         -19.11561 4428.46265  -0.004    0.997
## PopulationLaFortune       -0.34339    1.11910  -0.307    0.759
## PopulationLong Point     -19.01828 6316.19271  -0.003    0.998
## PopulationPond Mills     -18.89760 9370.71435  -0.002    0.998
## PopulationStarky Hill     -0.08014    0.86705  -0.092    0.926
```

```
## ---
## Signif. codes:  0 '***' 0.001 '**' 0.01 '*' 0.05 '.' 0.1 ' ' 1
##
## (Dispersion parameter for binomial family taken to be 1)
##
##     Null deviance: 35.815  on 84  degrees of freedom
## Residual deviance: 24.467  on 78  degrees of freedom
## AIC: 51.003
##
## Number of Fisher Scoring iterations: 20
```

```
mod37b<- glm(cbind(Other, noOTH)~1, family=binomial, data=dat3)
anova(mod37,mod37b, test="Chisq")
```

```
## Analysis of Deviance Table
##
## Model 1: cbind(Other, noOTH) ~ Population
## Model 2: cbind(Other, noOTH) ~ 1
##   Resid. Df Resid. Dev Df Deviance Pr(>Chi)
## 1        78     24.467
## 2        84     35.815 -6  -11.347  0.07822 .
## ---
## Signif. codes:  0 '***' 0.001 '**' 0.01 '*' 0.05 '.' 0.1 ' ' 1
```

```
#plot by population
boxplot(Pother~Population, data=dat3)
```

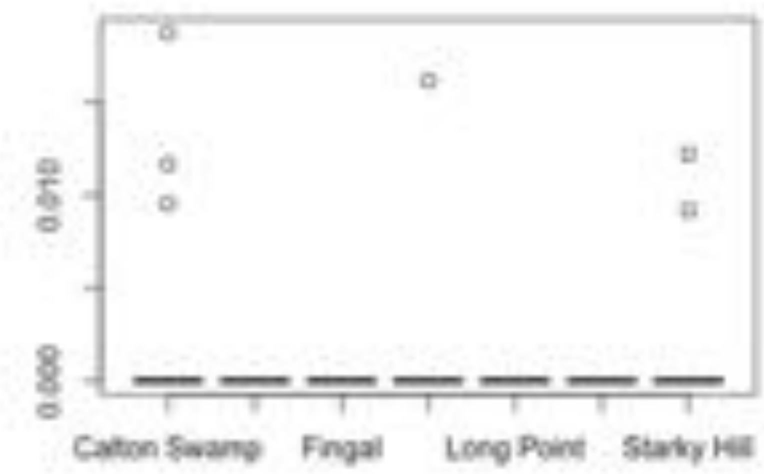

```
#test effect of body condition on sperm abnormalities
mod38 <- glm(cbind(Abnormal, noAb)~Population+SMI, family=binomial, data=dat3)
summary(mod38) #seriously overdispersed

##
## Call:
## glm(formula = cbind(Abnormal, noAb) ~ Population + SMI, family = binomial,
##     data = dat3)
##
## Deviance Residuals:
##     Min       1Q   Median       3Q      Max
## -6.3584  -1.8432  -0.0592   1.7257   8.0531
##
## Coefficients:
##                         Estimate Std. Error z value Pr(>|z|)
## (Intercept)           -2.898e-01  2.936e-01  -0.987 0.323608
## PopulationDereham      1.856e-01  8.138e-02   2.280 0.022599 *
## PopulationFingal       4.077e-02  7.205e-02   0.566 0.571526
## PopulationLaFortune    3.349e-01  1.005e-01   3.331 0.000864 ***
## PopulationLong Point   3.539e-05  9.227e-02   0.000 0.999694
## PopulationPond Mills  -2.177e-01  1.330e-01  -1.636 0.101763
## PopulationStarky Hill -6.611e-02  9.099e-02  -0.727 0.467514
## SMI                    1.917e-01  1.188e-01   1.613 0.106808
## ---
## Signif. codes:  0 '***' 0.001 '**' 0.01 '*' 0.05 '.' 0.1 ' ' 1
##
## (Dispersion parameter for binomial family taken to be 1)
##
##     Null deviance: 725.89  on 84  degrees of freedom
## Residual deviance: 695.32  on 77  degrees of freedom
## AIC: 1103.6
##
## Number of Fisher Scoring iterations: 4

mod38x <- glm.binomial.disp(mod38) #correct for overdispersion

##
## Binomial overdispersed logit model fitting...
## Iter.  1  phi: 0.09296549
## Iter.  2  phi: 0.09186404
## Iter.  3  phi: 0.09186226
## Iter.  4  phi: 0.09186226
## Converged after 4 iterations.
## Estimated dispersion parameter: 0.09186226
##
```

```
## Call:
## glm(formula = cbind(Abnormal, noAb) ~ Population + SMI, family = binomial,
##     data = dat3, weights = disp.weights)
##
## Deviance Residuals:
##     Min       1Q   Median       3Q      Max
## -2.1256  -0.6747  -0.1438   0.6431   2.6392
##
## Coefficients:
##                        Estimate Std. Error z value Pr(>|z|)
## (Intercept)            -0.61085    0.86832  -0.703    0.482
## PopulationDereham       0.24226    0.24615   0.984    0.325
## PopulationFingal        0.01567    0.21277   0.074    0.941
## PopulationLaFortune     0.21435    0.26083   0.822    0.411
## PopulationLong Point   -0.03553    0.26228  -0.135    0.892
## PopulationPond Mills   -0.28069    0.36320  -0.773    0.440
## PopulationStarky Hill  -0.16222    0.26160  -0.620    0.535
## SMI                     0.33650    0.35001   0.961    0.336
##
## (Dispersion parameter for binomial family taken to be 1)
##
##     Null deviance: 89.509  on 84  degrees of freedom
## Residual deviance: 83.980  on 77  degrees of freedom
## AIC: 149.62
##
## Number of Fisher Scoring iterations: 4
```

```
anova(mod38x,mod31a, test="Chisq")
```

```
## Analysis of Deviance Table
##
## Model 1: cbind(Abnormal, noAb) ~ Population + SMI
## Model 2: cbind(Abnormal, noAb) ~ Population
##   Resid. Df Resid. Dev Df Deviance Pr(>Chi)
## 1        77      83.98
## 2        78      85.34 -1  -1.3598   0.2436
```

```
mod38b <- glm(cbind(NoTail, no0t)~Population+SMI, family=binomial, data=dat3)
summary(mod38b) #seriously overdispersed
```

```
##
## Call:
## glm(formula = cbind(NoTail, no0t) ~ Population + SMI, family = binomial,
##     data = dat3)
##
```

```
## Deviance Residuals:
##     Min       1Q   Median       3Q      Max
## -4.7965  -1.7091  -0.5595   0.8849   7.0005
##
## Coefficients:
##                       Estimate Std. Error z value Pr(>|z|)
## (Intercept)            -4.3245     0.4667  -9.265  < 2e-16 ***
## PopulationDereham      -1.1833     0.1369  -8.643  < 2e-16 ***
## PopulationFingal       -0.4829     0.1094  -4.413 1.02e-05 ***
## PopulationLaFortune    -0.1942     0.1393  -1.394  0.16322
## PopulationLong Point   -0.7510     0.1591  -4.721 2.34e-06 ***
## PopulationPond Mills   -0.6599     0.2126  -3.103  0.00191 **
## PopulationStarky Hill  -0.8836     0.1449  -6.096 1.09e-09 ***
## SMI                     1.0817     0.1868   5.792 6.94e-09 ***
## ---
## Signif. codes:  0 '***' 0.001 '**' 0.01 '*' 0.05 '.' 0.1 ' ' 1
##
## (Dispersion parameter for binomial family taken to be 1)
##
##     Null deviance: 532.71  on 84  degrees of freedom
## Residual deviance: 426.15  on 77  degrees of freedom
## AIC: 738.47
##
## Number of Fisher Scoring iterations: 5
```

```
mod38bx  <- glm.binomial.disp(mod38b)
```

```
##
## Binomial overdispersed logit model fitting...
## Iter.  1  phi: 0.06075788
## Iter.  2  phi: 0.06369414
## Iter.  3  phi: 0.06371914
## Iter.  4  phi: 0.06371935
## Iter.  5  phi: 0.06371935
## Converged after 5 iterations.
## Estimated dispersion parameter: 0.06371935
##
## Call:
## glm(formula = cbind(NoTail, no0t) ~ Population + SMI, family = binomial,
##     data = dat3, weights = disp.weights)
##
## Deviance Residuals:
##     Min       1Q   Median       3Q      Max
## -1.9549  -0.7580  -0.2967   0.3286   2.7485
```

```
##
## Coefficients:
##                         Estimate Std. Error z value Pr(>|z|)
## (Intercept)              -4.5748     1.1566  -3.955 7.64e-05 ***
## PopulationDereham        -1.1287     0.3401  -3.319 0.000902 ***
## PopulationFingal         -0.5071     0.2730  -1.858 0.063236 .
## PopulationLaFortune      -0.3200     0.3182  -1.006 0.314619
## PopulationLong Point     -0.7865     0.3811  -2.064 0.039062 *
## PopulationPond Mills     -0.6490     0.4824  -1.345 0.178509
## PopulationStarky Hill    -0.9765     0.3561  -2.743 0.006097 **
## SMI                       1.1974     0.4599   2.604 0.009219 **
## ---
## Signif. codes:  0 '***' 0.001 '**' 0.01 '*' 0.05 '.' 0.1 ' ' 1
##
## (Dispersion parameter for binomial family taken to be 1)
##
##     Null deviance: 91.891  on 84  degrees of freedom
## Residual deviance: 73.994  on 77  degrees of freedom
## AIC: 141.15
##
## Number of Fisher Scoring iterations: 5
```

```
anova(mod38bx,mod33a, test="Chisq")
```

```
## Analysis of Deviance Table
##
## Model 1: cbind(NoTail, no0t) ~ Population + SMI
## Model 2: cbind(NoTail, no0t) ~ Population
##   Resid. Df Resid. Dev Df Deviance Pr(>Chi)
## 1        77     73.994
## 2        78     71.368 -1   2.6261
```

```
mod38c <- glm(cbind(NoHead, no0H)~Population+SMI, family=binomial, data=dat3)
summary(mod38c) #no overdispersion
```

```
##
## Call:
## glm(formula = cbind(NoHead, no0H) ~ Population + SMI, family = binomial,
##     data = dat3)
##
## Deviance Residuals:
##     Min       1Q   Median       3Q      Max
## -1.8186  -1.0966  -0.8020   0.5961   3.0159
##
## Coefficients:
```

```
##                        Estimate Std. Error z value Pr(>|z|)    
## (Intercept)             -6.7374     1.3649  -4.936 7.96e-07 ***
## PopulationDereham       -1.1919     0.4557  -2.616  0.00891 ** 
## PopulationFingal         0.3353     0.2878   1.165  0.24396    
## PopulationLaFortune     -0.3092     0.4638  -0.667  0.50495    
## PopulationLong Point    -0.2362     0.4371  -0.540  0.58889    
## PopulationPond Mills    -0.7723     0.7446  -1.037  0.29964    
## PopulationStarky Hill   -0.8252     0.4633  -1.781  0.07489 .  
## SMI                      0.9992     0.5422   1.843  0.06534 .  
## ---
## Signif. codes:  0 '***' 0.001 '**' 0.01 '*' 0.05 '.' 0.1 ' ' 1
## 
## (Dispersion parameter for binomial family taken to be 1)
## 
##     Null deviance: 133.93  on 84  degrees of freedom
## Residual deviance: 117.39  on 77  degrees of freedom
## AIC: 236.22
## 
## Number of Fisher Scoring iterations: 5
```

```
anova(mod38c, mod34a, test="Chisq")
```

```
## Analysis of Deviance Table
## 
## Model 1: cbind(NoHead, no0H) ~ Population + SMI
## Model 2: cbind(NoHead, no0H) ~ Population
##   Resid. Df Resid. Dev Df Deviance Pr(>Chi)
## 1        77    117.395                     
## 2        78     70.948 -1   46.447
```

```
mod38d <- glm(cbind(TwoHeads, no2H)~Population+SMI, family=binomial, data=dat3)
summary(mod38d) #underdispersed
```

```
## 
## Call:
## glm(formula = cbind(TwoHeads, no2H) ~ Population + SMI, family = binomial, 
##     data = dat3)
## 
## Deviance Residuals: 
##      Min        1Q    Median        3Q       Max  
## -0.79523  -0.00003  -0.00002  -0.00001   1.93654  
## 
## Coefficients:
##                          Estimate Std. Error z value Pr(>|z|)
## (Intercept)              -30.8765 11709.9621  -0.003    0.998
```

```
## PopulationDereham        -0.4223 17741.2954   0.000    1.000
## PopulationFingal         19.8459 11709.9549   0.002    0.999
## PopulationLaFortune       0.3454 19872.1735   0.000    1.000
## PopulationLong Point      0.1747 20751.6338   0.000    1.000
## PopulationPond Mills     21.3160 11709.9549   0.002    0.999
## PopulationStarky Hill    -0.3559 19258.9721   0.000    1.000
## SMI                       1.5176     5.1827   0.293    0.770
##
## (Dispersion parameter for binomial family taken to be 1)
##
##     Null deviance: 15.0768  on 84  degrees of freedom
## Residual deviance:  8.2985  on 77  degrees of freedom
## AIC: 28.273
##
## Number of Fisher Scoring iterations: 22
```

```
anova(mod38d,mod36, test="Chisq")
```

```
## Analysis of Deviance Table
##
## Model 1: cbind(TwoHeads, no2H) ~ Population + SMI
## Model 2: cbind(TwoHeads, no2H) ~ Population
##   Resid. Df Resid. Dev Df Deviance Pr(>Chi)
## 1        77     8.2985
## 2        78     8.3872 -1 -0.08867   0.7659
```

```
mod38e <- glm(cbind(Other, noOTH)~Population+SMI, family=binomial, data=dat3)
summary(mod38e) #underdispersed
```

```
##
## Call:
## glm(formula = cbind(Other, noOTH) ~ Population + SMI, family = binomial,
##     data = dat3)
##
## Deviance Residuals:
##      Min        1Q    Median        3Q       Max
## -0.97015  -0.47366  -0.00005  -0.00004   1.62022
##
## Coefficients:
##                         Estimate Std. Error z value Pr(>|z|)
## (Intercept)              -1.1663     4.3635  -0.267    0.789
## PopulationDereham       -18.6341  4879.0592  -0.004    0.997
## PopulationFingal        -19.1174  4374.4120  -0.004    0.997
## PopulationLaFortune      -0.2003     1.1284  -0.178    0.859
## PopulationLong Point    -19.0526  6226.1383  -0.003    0.998
```

```
## PopulationPond Mills   -18.4524  9341.0785  -0.002    0.998
## PopulationStarky Hill    0.4701     0.9848   0.477    0.633
## SMI                     -2.0202     1.8444  -1.095    0.273
##
## (Dispersion parameter for binomial family taken to be 1)
##
##     Null deviance: 35.815  on 84  degrees of freedom
## Residual deviance: 23.197  on 77  degrees of freedom
## AIC: 51.733
##
## Number of Fisher Scoring iterations: 20
```

```
anova(mod38e,mod37, test="Chisq")
```

```
## Analysis of Deviance Table
##
## Model 1: cbind(Other, noOTH) ~ Population + SMI
## Model 2: cbind(Other, noOTH) ~ Population
##   Resid. Df Resid. Dev Df Deviance Pr(>Chi)
## 1        77     23.197
## 2        78     24.467 -1    -1.27   0.2598
```

4.5 Comparing mitochondrial lineages

Compare lineages with respect to body size, body condition and sperm traits by fitting GLMM with Population as random variable; model mod44k reported in text

```
#compare body size
mod41 <- lmer(PC1~Lineage+scale(DOY)+scale(DOY2)+(1|Population), data=dat1)
summary(mod41)
```

```
## Linear mixed model fit by REML ['lmerMod']
## Formula: PC1 ~ Lineage + scale(DOY) + scale(DOY2) + (1 | Population)
##    Data: dat1
##
## REML criterion at convergence: 213.6
##
## Scaled residuals:
##     Min      1Q  Median      3Q     Max
## -3.4724 -0.4465 -0.0022  0.6513  2.2147
##
## Random effects:
##  Groups     Name        Variance Std.Dev.
##  Population (Intercept) 0.8954   0.9462
##  Residual               0.6534   0.8083
## Number of obs: 86, groups:  Population, 7
```

```
##
## Fixed effects:
##                  Estimate Std. Error t value
## (Intercept)      -0.09770    0.58563  -0.167
## LineageEastern   -0.19639    1.14330  -0.172
## LineageInterior  -0.02511    0.93142  -0.027
## scale(DOY)        0.23646   15.54508   0.015
## scale(DOY2)      -0.56248   15.54252  -0.036
##
## Correlation of Fixed Effects:
##              (Intr) LngEst LngInt s(DOY)
## LineagEstrn -0.647
## LineagIntrr -0.638  0.407
## scale(DOY)  -0.281  0.624  0.168
## scale(DOY2)  0.282 -0.625 -0.171 -1.000
```

```
qqnorm(residuals(mod41))
```

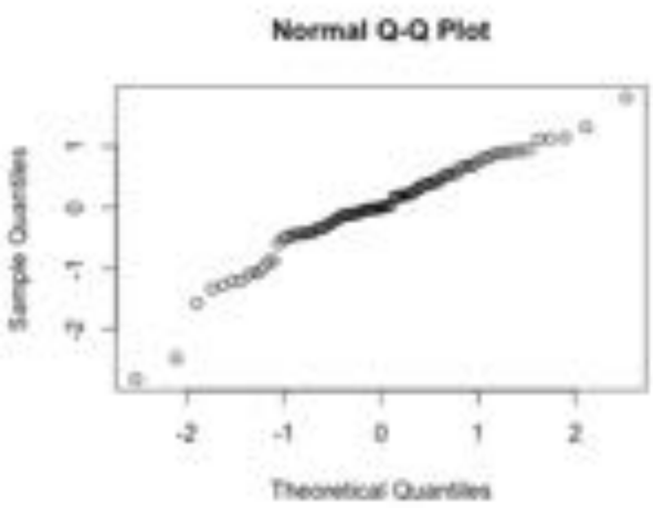


```
shapiro.test(residuals(mod41))
```

```
##
##  Shapiro-Wilk normality test
##
## data:  residuals(mod41)
## W = 0.95179, p-value = 0.00286
```

```
mod41a <- lmer(PC1~scale(DOY)+scale(DOY2)+(1|Population), data=dat1)
anova(mod41,mod41a) #no differences among lineages wrt body size
```

```
## refitting model(s) with ML (instead of REML)
```

```
## Data: dat1
## Models:
```

```
## mod41a: PC1 ~ scale(DOY) + scale(DOY2) + (1 | Population)
## mod41: PC1 ~ Lineage + scale(DOY) + scale(DOY2) + (1 | Population)
##        Df    AIC   BIC  logLik deviance Chisq Chi Df Pr(>Chisq)
## mod41a  5 230.73 243.0 -110.37   220.73
## mod41   7 234.72 251.9 -110.36   220.72 0.008      2      0.996

#rerun model without outliers
dat2 <- subset(dat1, Individual != "11-076" & Individual != "11-106")

mod41b <- lmer(PC1~Lineage+DOY+(1|Population), data=dat2) #removed the two outliers
mod41c <- lmer(PC1~DOY+(1|Population), data=dat2)
shapiro.test(residuals(mod41b))

##
##  Shapiro-Wilk normality test
##
## data:  residuals(mod41b)
## W = 0.98413, p-value = 0.391

anova(mod41b, mod41c) #same conclusion, no differences among lineages wrt body size

## refitting model(s) with ML (instead of REML)

## Data: dat2
## Models:
## mod41c: PC1 ~ DOY + (1 | Population)
## mod41b: PC1 ~ Lineage + DOY + (1 | Population)
##         Df    AIC    BIC  logLik deviance  Chisq Chi Df Pr(>Chisq)
## mod41c  4 198.74 208.46 -95.371   190.74
## mod41b  6 202.47 217.05 -95.234   190.47 0.2734      2     0.8722

#body condition
mod43 <- lmer(SMI~Lineage+(1|Population), data=dat1)
summary(mod43)

## Linear mixed model fit by REML ['lmerMod']
## Formula: SMI ~ Lineage + (1 | Population)
##    Data: dat1
##
## REML criterion at convergence: -7.1
##
## Scaled residuals:
##      Min       1Q   Median       3Q      Max
## -2.10006 -0.70559 -0.07999  0.50068  2.23502
##
## Random effects:
##  Groups     Name        Variance Std.Dev.
```

```
##  Population (Intercept) 0.01201  0.1096
##  Residual               0.04472  0.2115
## Number of obs: 86, groups:  Population, 7
##
## Fixed effects:
##                 Estimate Std. Error t value
## (Intercept)      2.60584    0.07098   36.71
## LineageEastern  -0.15118    0.11577   -1.31
## LineageInterior -0.10472    0.11815   -0.89
##
## Correlation of Fixed Effects:
##             (Intr) LngEst
## LineagEstrn -0.613
## LineagIntrr -0.601  0.368
```

```
qqnorm(residuals(mod43))
```

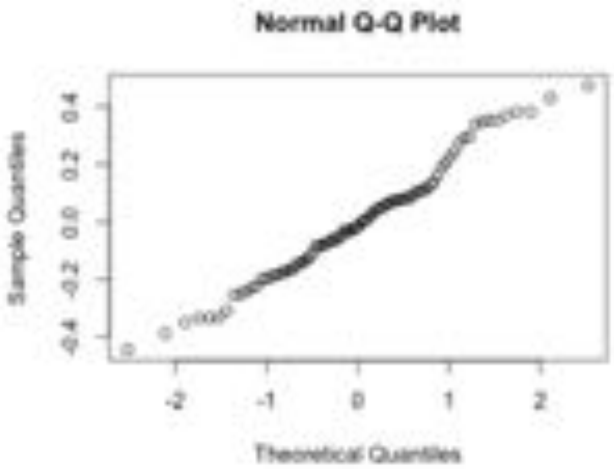


```
shapiro.test(residuals(mod43))
```

```
##
##  Shapiro-Wilk normality test
##
## data:  residuals(mod43)
## W = 0.98228, p-value = 0.2872
```

```
mod43a<- lmer(SMI~(1|Population), data=dat1)
anova(mod43,mod43a) #lineages not different wrt body condition
```

```
## refitting model(s) with ML (instead of REML)
```

```
## Data: dat1
## Models:
## mod43a: SMI ~ (1 | Population)
```

```
## mod43: SMI ~ Lineage + (1 | Population)
##        Df     AIC     BIC logLik deviance  Chisq Chi Df Pr(>Chisq)
## mod43a  3 -8.5344 -1.1714 7.2672  -14.534
## mod43   5 -7.1989  5.0728 8.5995  -17.199 2.6645      2     0.2639

#sperm length
mod44 <- lmer(TOTspermlen~Lineage+(1|Population)+scale(DOY2)+scale(DOY), data=dat1)
#main effects only, no interactions
summary(mod44)

## Linear mixed model fit by REML ['lmerMod']
## Formula:
## TOTspermlen ~ Lineage + (1 | Population) + scale(DOY2) + scale(DOY)
##    Data: dat1
##
## REML criterion at convergence: 602.3
##
## Scaled residuals:
##      Min       1Q   Median       3Q      Max
## -2.62434 -0.57468 -0.02078  0.56754  2.17751
##
## Random effects:
##  Groups     Name        Variance Std.Dev.
##  Population (Intercept)  1.419   1.191
##  Residual               88.234   9.393
## Number of obs: 86, groups:  Population, 7
##
## Fixed effects:
##                  Estimate Std. Error t value
## (Intercept)        45.819      2.025  22.631
## LineageEastern     18.368      6.310   2.911
## LineageInterior     7.688      2.914   2.638
## scale(DOY2)      -495.829    124.080  -3.996
## scale(DOY)        493.446    124.228   3.972
##
## Correlation of Fixed Effects:
##             (Intr) LngEst LngInt s(DOY2
## LineagEstrn -0.749
## LineagIntrr -0.488  0.175
## scale(DOY2)  0.618 -0.896 -0.037
## scale(DOY)  -0.617  0.896  0.034 -1.000

qqnorm(residuals(mod44))
```

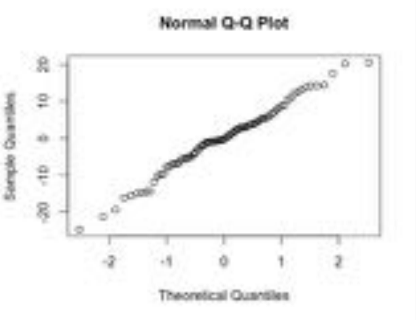


```
shapiro.test(residuals(mod44))

##
##  Shapiro-Wilk normality test
##
## data:  residuals(mod44)
## W = 0.98766, p-value = 0.5928

summary(glht(mod44, linfct=mcp(Lineage="Tukey")))

##
##   Simultaneous Tests for General Linear Hypotheses
##
## Multiple Comparisons of Means: Tukey Contrasts
##
##
## Fit: lmer(formula = TOTspermlen ~ Lineage + (1 | Population) + scale(DOY2) +
##     scale(DOY), data = dat1)
##
## Linear Hypotheses:
##                           Estimate Std. Error z value Pr(>|z|)
## Eastern - Contact == 0    18.368      6.310   2.911  0.00911 **
## Interior - Contact == 0    7.688      2.914   2.638  0.02066 *
## Interior - Eastern == 0  -10.680      6.471  -1.650  0.21171
## ---
## Signif. codes:  0 '***' 0.001 '**' 0.01 '*' 0.05 '.' 0.1 ' ' 1
## (Adjusted p values reported -- single-step method)

mod44a<- lmer(TOTspermlen~(1|Population)+scale(DOY2)+scale(DOY), data=dat1)
anova(mod44,mod44a) #no diff wrt sperm length

## refitting model(s) with ML (instead of REML)

## Data: dat1
## Models:
## mod44a: TOTspermlen ~ (1 | Population) + scale(DOY2) + scale(DOY)
```

```
## mod44: TOTspermlen ~ Lineage + (1 | Population) + scale(DOY2) + scale(DOY)
##         Df    AIC    BIC  logLik deviance Chisq Chi Df Pr(>Chisq)
## mod44a  5 647.10 659.37 -318.55   637.10
## mod44   7 638.76 655.94 -312.38   624.76 12.34      2   0.002091 **
## ---
## Signif. codes:  0 '***' 0.001 '**' 0.01 '*' 0.05 '.' 0.1 ' ' 1
```

```
#check for interactions
mod44i <- lmer(TOTspermlen~Lineage*scale(DOY)+Lineage*scale(DOY2)+(1|Population),
data=dat1)
mod44iRM <- lmer(TOTspermlen~Lineage*scale(DOY)+Lineage*scale(DOY2)+(1|Population),
REML=FALSE,data=dat1)
m441RM <- dredge(mod44iRM)
```

```
## Fixed term is "(Intercept)"
```

```
subset (m441RM,delta<2.5)
```

```
## Global model call: lmer(formula = TOTspermlen ~ Lineage * scale(DOY) + Lineage *
##     scale(DOY2) + (1 | Population), data = dat1, REML = FALSE)
## ---
## Model selection table
##    (Int) Lng scl(DOY) scl(DOY2) Lng:scl(DOY) Lng:scl(DOY2) df   logLik
## 12 51.20   +   -1.406                         +                  8 -308.997
## 22 51.18   +             -1.417                           +      8 -309.035
## 16 50.12   +  103.100  -104.500              +                  9 -308.830
## 24 50.11   +  103.700  -105.100                           +      9 -308.871
##     AICc delta weight
## 12 635.9  0.00  0.381
## 22 635.9  0.08  0.366
## 16 638.0  2.16  0.129
## 24 638.1  2.25  0.124
## Models ranked by AICc(x)
## Random terms (all models):
## '1 | Population'
```

```
mod44j <- lmer(TOTspermlen~Lineage*scale(DOY)+scale(DOY2)+(1|Population), data=dat1)
summary(mod44j)
```

```
## Linear mixed model fit by REML ['lmerMod']
## Formula:
## TOTspermlen ~ Lineage * scale(DOY) + scale(DOY2) + (1 | Population)
##    Data: dat1
##
## REML criterion at convergence: 586
##
## Scaled residuals:
```

```
##      Min       1Q   Median       3Q      Max
## -2.47526 -0.51042  0.02827  0.58270  2.46891
##
## Random effects:
##  Groups     Name        Variance Std.Dev.
##  Population (Intercept)  0.00    0.000
##  Residual               83.86    9.157
## Number of obs: 86, groups:  Population, 7
##
## Fixed effects:
##                              Estimate Std. Error t value
## (Intercept)                   50.1168     2.4271  20.649
## LineageEastern                 0.2830     8.9733   0.032
## LineageInterior               20.0909     5.5038   3.650
## scale(DOY)                   103.1054   188.4182   0.547
## scale(DOY2)                 -104.5241   188.4316  -0.555
## LineageEastern:scale(DOY)     -0.5804     2.3455  -0.247
## LineageInterior:scale(DOY)  -22.0255     8.4676  -2.601
##
## Correlation of Fixed Effects:
##             (Intr) LngEst LngInt s(DOY) s(DOY2 LE:(DO
## LineagEstrn -0.866
## LineagIntrr  0.402 -0.624
## scale(DOY)  -0.801  0.958 -0.696
## scale(DOY2)  0.804 -0.959  0.695 -1.000
## LngEs:(DOY) -0.139  0.029  0.090 -0.036  0.027
## LngIn:(DOY) -0.662  0.748 -0.854  0.766 -0.768  0.168
```

```
Anova(mod44j)
```

```
## Analysis of Deviance Table (Type II Wald chisquare tests)
##
## Response: TOTspermlen
##                      Chisq Df Pr(>Chisq)
## Lineage            16.1371  2  0.0003132 ***
## scale(DOY)         17.1104  1  3.527e-05 ***
## scale(DOY2)         0.3077  1  0.5790960
## Lineage:scale(DOY)  6.8027  2  0.0333284 *
## ---
## Signif. codes:  0 '***' 0.001 '**' 0.01 '*' 0.05 '.' 0.1 ' ' 1
```

```
mod44k <- lmer(TOTspermlen~Lineage*scale(DOY)+(1|Population), data=dat1) #this is the best-fitting model
anova(mod44j, mod44k) #removing 2nd order date term had no sig effect on model
```

```
## refitting model(s) with ML (instead of REML)
```

```
## Data: dat1
## Models:
## mod44k: TOTspermlen ~ Lineage * scale(DOY) + (1 | Population)
## mod44j: TOTspermlen ~ Lineage * scale(DOY) + scale(DOY2) + (1 | Population)
##        Df    AIC    BIC  logLik deviance  Chisq Chi Df Pr(>Chisq)
## mod44k  8 633.99 653.63 -309.00   617.99
## mod44j  9 635.66 657.75 -308.83   617.66 0.3343      1     0.5631
```

```
Anova(mod44k)
```

```
## Analysis of Deviance Table (Type II Wald chisquare tests)
##
## Response: TOTspermlen
##                        Chisq Df Pr(>Chisq)
## Lineage              16.7893  2  0.0002261 ***
## scale(DOY)            7.4383  1  0.0063852 **
## Lineage:scale(DOY) 24.0202  2  6.083e-06 ***
## ---
## Signif. codes:  0 '***' 0.001 '**' 0.01 '*' 0.05 '.' 0.1 ' ' 1
```

```
summary(glht(mod44k, linfct=mcp(Lineage="Tukey"))) #interaction term makes this
difficult to interpret
```

```
##
##   Simultaneous Tests for General Linear Hypotheses
##
## Multiple Comparisons of Means: Tukey Contrasts
##
##
## Fit: lmer(formula = TOTspermlen ~ Lineage * scale(DOY) + (1 | Population),
##     data = dat1)
##
## Linear Hypotheses:
##                          Estimate Std. Error z value Pr(>|z|)
## Eastern - Contact == 0    -4.488      2.545  -1.764    0.175
## Interior - Contact == 0   22.212      3.941   5.636   <0.001 ***
## Interior - Eastern == 0   26.701      4.227   6.316   <0.001 ***
## ---
## Signif. codes:  0 '***' 0.001 '**' 0.01 '*' 0.05 '.' 0.1 ' ' 1
## (Adjusted p values reported -- single-step method)
```

```
lsmeans(mod44k, pairwise~Lineage | DOY)
```

```
## $lsmeans
## DOY = 127.5814:
##  Lineage    lsmean       SE   df lower.CL upper.CL
##  Contact  51.19865 1.533008 1.95 44.45204 57.94527
```

```
## Eastern 46.71031 2.099343 4.59 41.16559 52.25503
## Interior 73.41081 4.005613 3.25 61.20843 85.61319
##
## Confidence level used: 0.95
##
## $contrasts
## DOY = 127.5814:
## contrast estimate SE df t.ratio p.value
## Contact - Eastern 4.488345 2.599491 3.36 1.727 0.3192
## Contact - Interior -22.212155 4.288945 3.02 -5.179 0.0279
## Eastern - Interior -26.700500 4.522408 3.52 -5.904 0.0129
##
## P value adjustment: tukey method for comparing a family of 3 estimates
```

```
boxplot(TOTspermlen~Lineage, data=dat1)
```

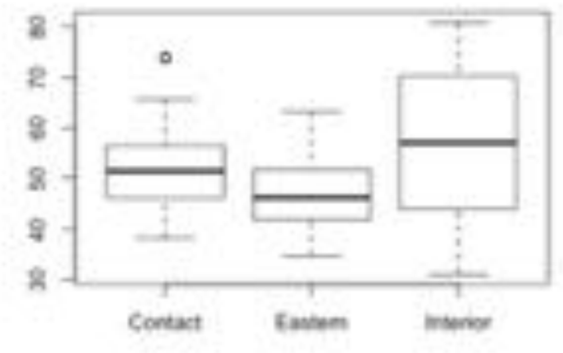


```
(cv44k <-rmse(residuals(mod44k)/mean(dat1$TOTspermlen) *100)) #coeff of variation, controlling for date and population
```

```
## [1] 16.95677
```

4.5.1 Compare lineages with respect to sperm abnormalities

This analysis fits a GLMM wth Population as random effect; analyses reported in Table 1

```
dispersion = 1:length(dat3$TotalSperm)

mod51 <- glmer(cbind(Abnormal,noAb)~Lineage+(1|Population)+(1|dispersion),
family=binomial,data=dat3) #seriously overdispersed
overdisp.glmer(mod51) #underdispersed but OK
```

```
## Residual deviance: 19.92 on 80 degrees of freedom (ratio: 0.249)
```

```
mod51a <- glmer(cbind(Abnormal,noAb)~(1|Population)+(1|dispersion),
family=binomial,data=dat3)
anova(mod51a, mod51)
```

```
## Data: dat3
## Models:
## mod51a: cbind(Abnormal, noAb) ~ (1 | Population) + (1 | dispersion)
## mod51: cbind(Abnormal, noAb) ~ Lineage + (1 | Population) + (1 | dispersion)
##        Df    AIC    BIC  logLik deviance  Chisq Chi Df Pr(>Chisq)
## mod51a  3 679.55 686.88 -336.78   673.55
## mod51   5 682.90 695.11 -336.45   672.90 0.6515      2      0.722
```

```
mod52 <- glmer(cbind(CytoDrop,noCD)~Lineage+(1|Population)+(1|dispersion),
family=binomial,data=dat3)
overdisp.glmer(mod52) #underdispersed but OK
```

```
## Residual deviance: 13.277 on 80 degrees of freedom (ratio: 0.166)
```

```
mod52a <- glmer(cbind(CytoDrop, noCD)~(1|Population)+(1|dispersion),
family=binomial,data=dat3)
anova(mod52a, mod52)
```

```
## Data: dat3
## Models:
## mod52a: cbind(CytoDrop, noCD) ~ (1 | Population) + (1 | dispersion)
## mod52: cbind(CytoDrop, noCD) ~ Lineage + (1 | Population) + (1 | dispersion)
##        Df    AIC    BIC  logLik deviance  Chisq Chi Df Pr(>Chisq)
## mod52a  3 682.81 690.14 -338.40   676.81
## mod52   5 684.76 696.98 -337.38   674.76 2.0462      2     0.3595
```

```
mod53 <- glmer(cbind(NoTail,no0t)~Lineage+(1|Population)+(1|dispersion),
family=binomial,data=dat3)
overdisp.glmer(mod53) #underdispersed but OK
```

```
## Residual deviance: 28.577 on 80 degrees of freedom (ratio: 0.357)
```

```
mod53a <- glmer(cbind(NoTail,no0t)~(1|Population)+(1|dispersion),
family=binomial,data=dat3)
anova(mod53a, mod53)
```

```
## Data: dat3
## Models:
## mod53a: cbind(NoTail, no0t) ~ (1 | Population) + (1 | dispersion)
## mod53: cbind(NoTail, no0t) ~ Lineage + (1 | Population) + (1 | dispersion)
##        Df    AIC    BIC  logLik deviance  Chisq Chi Df Pr(>Chisq)
## mod53a  3 539.38 546.71 -266.69   533.38
## mod53   5 543.20 555.42 -266.60   533.20 0.1792      2     0.9143
```

```
mod54 <- glmer(cbind(NoHead, no0H)~Lineage+(1|Population), family=binomial, data=dat3)
overdisp.glmer(mod54) #slightly overdispersed
```

```
## Residual deviance: 126.644 on 81 degrees of freedom (ratio: 1.564)
```

```
mod54a <- glmer(cbind(NoHead, no0H)~(1|Population), family=binomial, data=dat3)
anova(mod54, mod54a, test="Chisq")
```

```
## Data: dat3
## Models:
## mod54a: cbind(NoHead, no0H) ~ (1 | Population)
## mod54: cbind(NoHead, no0H) ~ Lineage + (1 | Population)
##        Df    AIC    BIC  logLik deviance Chisq Chi Df Pr(>Chisq)
## mod54a  2 237.85 242.73 -116.92   233.85
## mod54   4 239.26 249.03 -115.63   231.26 2.583      2     0.2749

mod55 <- glmer(cbind(TwoTails, no2t)~Lineage+(1|Population), family=binomial,
data=dat3)
overdisp.glmer(mod55) #slightly overdispersed

## Residual deviance: 143.54 on 81 degrees of freedom (ratio: 1.772)

mod55a <- glmer(cbind(TwoTails, no2t)~(1|Population), family=binomial, data=dat3)
anova(mod55, mod55a)

## Data: dat3
## Models:
## mod55a: cbind(TwoTails, no2t) ~ (1 | Population)
## mod55: cbind(TwoTails, no2t) ~ Lineage + (1 | Population)
##        Df    AIC    BIC  logLik deviance  Chisq Chi Df Pr(>Chisq)
## mod55a  2 253.53 258.41 -124.76   249.53
## mod55   4 246.47 256.24 -119.23   238.47 11.058      2    0.00397 **
## ---
## Signif. codes:  0 '***' 0.001 '**' 0.01 '*' 0.05 '.' 0.1 ' ' 1

#mod56 <- glmer(cbind(TwoHeads, no2H)~Lineage+(1|Population), family=binomial,
data=dat3) #model does not converge, possibly because sample sizes too small

mod56 <- glm(cbind(TwoHeads, no2H)~Lineage/Population, family=binomial, data=dat3)
#set Population as fixed effect nested within Lineage
Anova(mod56)

## Analysis of Deviance Table (Type II tests)
##
## Response: cbind(TwoHeads, no2H)
##                      LR Chisq Df Pr(>Chisq)
## Lineage                5.3652  2    0.06838 .
## Lineage:Population     1.3244  4    0.85723
## ---
## Signif. codes:  0 '***' 0.001 '**' 0.01 '*' 0.05 '.' 0.1 ' ' 1

#mod57 <- glmer(cbind(Other, noOTH)~Lineage+(1|Population), family=binomial,
data=dat3) #model does not converge, possibly because sample sizes too small
mod57 <- glm(cbind(Other, noOTH)~Lineage/Population, family=binomial, data=dat3) #set
Population as fixed effect nested within Lineage
Anova(mod57)
```

```
## Analysis of Deviance Table (Type II tests)
##
## Response: cbind(Other, noOTH)
##                     LR Chisq Df Pr(>Chisq)
## Lineage               4.7070  2    0.09503 .
## Lineage:Population   6.6402  4    0.15617
## ---
## Signif. codes:  0 '***' 0.001 '**' 0.01 '*' 0.05 '.' 0.1 ' ' 1
```
