# Supplementary material for: Extensive variation in sperm morphology in a frog with no sperm competition
Source: BMC Evol Biol. 2016 Feb 1;16:29. doi: 10.1186/s12862-016-0601-8 (PMC4735968; doi:10.1186/s12862-016-0601-8)
Supplement: Additional file 2: Table S1. — Top models (ΔAICc <2.0) for each of the analyses summarized in Table 1. (DOCX 13 kb) [file 12862_2016_601_MOESM2_ESM.docx]

**S1 Table.** Top models (**∆**AICc ≤2) to explain variation in sperm size traits. See Table 1 for a summary of the best-fitting model in each set. The sign of each of the predictors is indicated.

| **Response** | **Predictors** | **∆AICc** | **weight** |
| --- | --- | --- | --- |
| total sperm length | +date, –date^2^, –SMI | 0 | 0.36 |
|  | +date, –date^2^, –SMI, +PC1 | 0.28 | 0.32 |
|  | +date, –date^2^, +PC1 | 1.26 | 0.19 |
| sperm head length | –date | 0 | 0.23 |
|  | –date^2^ | 0.03 | 0.23 |
|  | –date, +PC1 | 0.78 | 0.15 |
|  | –date^2^, +PC1 | 0.83 | 0.15 |
| sperm head perimeter | –date^2^, +PC1 | 0 | 0.12 |
|  | –date, +PC1 | 0.02 | 0.12 |
|  | –date^2^ | 0.09 | 0.11 |
|  | –date | 0.13 | 0.11 |
|  | –date^2^, –SMI | 0.48 | 0.09 |
|  | +date, –SMI | 0.54 | 0.09 |
|  | –date^2^, –SMI, +PC1 | 0.97 | 0.07 |
|  | –date, –SMI, +PC1 | 1.01 | 0.07 |
|  | +PC1 | 1.84 | 0.05 |
