# Supplementary material for: Extensive variation in sperm morphology in a frog with no sperm competition
Source: BMC Evol Biol. 2016 Feb 1;16:29. doi: 10.1186/s12862-016-0601-8 (PMC4735968; doi:10.1186/s12862-016-0601-8)
Supplement: Additional file 3: Table S2. — Top models (AICc ≤2) testing for differences among populations in sperm size traits. (DOCX 14 kb) [file 12862_2016_601_MOESM3_ESM.docx]

**S2 Table.** Top models (**∆**AICc ≤2) testing for differences among populations in sperm size traits. The sign of each of the predictors is shown and the significant predictors in the best-fitting model in each set are indicated in bold.

| **Response** | **Predictors** | **∆AICc** | **weight** |
| --- | --- | --- | --- |
| total sperm length | **+date, –date^2^, Population**, –SMI | 0 | 0.29 |
|  | +date, –date^2^, Population | 1.25 | 0.17 |
|  |  |  |  |
| sperm head length | **–date^2^, Population** | 0 | 0.18 |
|  | +date, Population | 0.06 | 0.18 |
|  | –date^2^, –SMI, Population | 0.75 | 0.13 |
|  | –date, –SMI, Population | 0.83 | 0.012 |
|  | –date^2^, +PC1, Population | 1.17 | 0.10 |
|  | –date, +PC1, Population | 1.23 | 0.10 |
|  | +date, –date^2^, Population | 1.80 | 0.07 |
|  |  |  |  |
| sperm head perimeter | –date^2^, +PC1 | 0 | 0.12 |
|  | –date, +PC1 | 0.02 | 0.12 |
|  | –date^2^ | 0.09 | 0.11 |
|  | –date | 0.13 | 0.11 |
|  | –date^2^, –SMI | 0.48 | 0.09 |
|  | –date, –SMI | 0.54 | 0.09 |
|  | –date^2^, –SMI, +PC1 | 0.97 | 0.07 |
|  | –date, –SMI, +PC1 | 1.01 | 0.07 |
|  | +PC1 | 1.84 | 0.05 |
